# Strong Hydrogen Bond Donating Solvents Accelerate the Passerini Three-Component Reaction

**DOI:** 10.1021/acs.joc.5c00236

**Published:** 2025-04-03

**Authors:** Claudio Ferdeghini, Minghui Wu, Prabhat Ranjan, Martien A. Würdemann, Jan Pyschik, Alexander Mitsos, Eelco Ruijter, Romano V.A. Orru, Thomas Hansen, Jordy M. Saya

**Affiliations:** a Biobased Organic Chemistry, Aachen-Maastricht Institute for Biobased Materials (AMIBM), Maastricht University, Urmonderbaan 22, Geleen 6167RD, The Netherlands; b Process Systems Engineering, RWTH Aachen University, Schinkelstrasse 8, Aachen 52062, Germany; c Department of Chemistry & Pharmaceutical Sciences and Amsterdam Institute for Molecular & Life Science (AIMMS), Vrije Universiteit Amsterdam, De Boelelaan 1108, Amsterdam 1081 HZ, The Netherlands

## Abstract

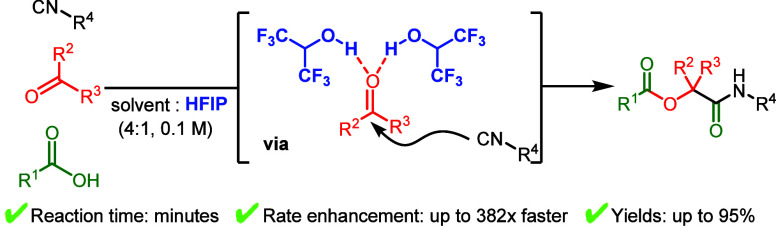

We report enhanced reaction rates of the Passerini reaction (P-3CR) using 1,1,1,3,3,3-hexafluoroisopropanol (HFIP) as a cosolvent. Although alcoholic solvents typically increase the energy barrier of the rate-determining step for the P-3CR, we observed significant rate enhancements even when employing strong hydrogen bond donating (HBD) alcohols as cosolvents. This rate enhancement was observed for most aprotic organic solvents, with the exception of strong hydrogen bond accepting (HBA) solvents such as DMF. Experimental kinetic studies and DFT calculations provided a mechanistic rationale for our observations. An investigation of the substrate scope showed that this rate enhancement generally resulted in a (slight) increase of the overall yield in the P-3CR.

## Introduction

Multicomponent reactions (MCRs) efficiently generate structural diversity and complexity, enabling the rapid synthesis of large compound libraries from a minimal number of components.^1^ MCRs also offer valuable structure–activity relationship (SAR) insights, facilitating focused libraries and scalable reactions for drug discovery and probe development.^2^ These characteristics make MCRs ideal for high-throughput screening (HTS), as demonstrated by Dömling, where MCRs have been integrated into HTS platforms to rapidly identify biologically active compounds from vast chemical libraries.^3^

**Figure 1:**
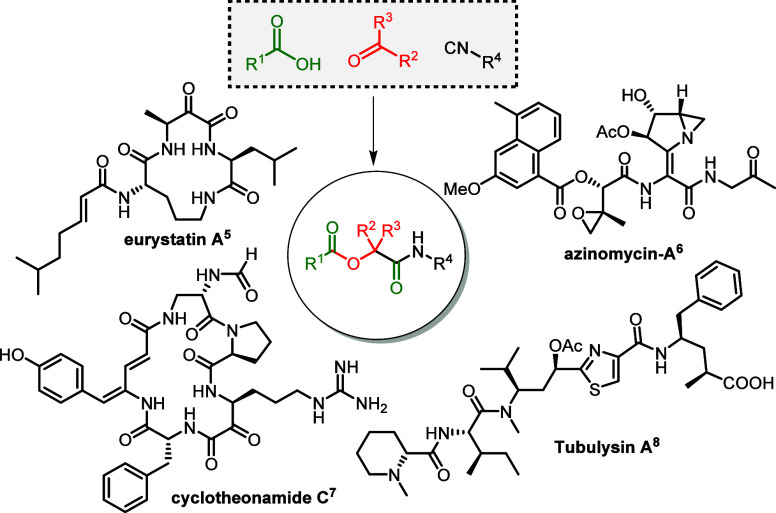
Applications of the Passerini reaction in natural product synthesis.

The Passerini reaction (P-3CR) is a highly versatile MCR, known for its efficiency, broad substrate tolerance, and atom economy.^4^ The P-3CR involves an isocyanide, an aldehyde or ketone, and a carboxylic acid, resulting in a functionalized α-acyloxyamides. The applicability of these α-acyloxyamides is exemplified by their use in the total synthesis of natural product (Figure 1).^5−8^

Despite its advantages, the P-3CR often requires long reaction times, which limits its use in HTS, where rapid reactions are essential.^9^ Thus, accelerating the P-3CR would expand its utility in HTS platforms. Innovative strategies that have been explored to accelerate the P-3CR include solvent optimization,^10^ neat conditions,^11^ elevated temperatures,^12^ and the implementation of novel techniques such as sonication,^13^ mechanochemistry,^14^ or increased pressure.^15^

Notably, computational studies by Morokuma et al. revealed that hydrogen bonding between the carboxylic acid and the aldehyde in the rate-determining step is crucial for the P-3CR.^16^ Interestingly, these studies also highlighted that alcoholic solvents slow down the reaction due to competing hydrogen bonding. In contrast, we found that strong hydrogen bond donating solvents like 2,2,2-trifluoroethanol (TFE) and 1,1,1,3,3,3-hexafluoroisopropanol (HFIP), facilitated isocyanide-based MCRs.^17^ This prompted us to explore if strong hydrogen bond donating (HBD) solvents could also enhance the kinetics of the classical P-3CR.

## Results and Discussion

**Figure 2:**
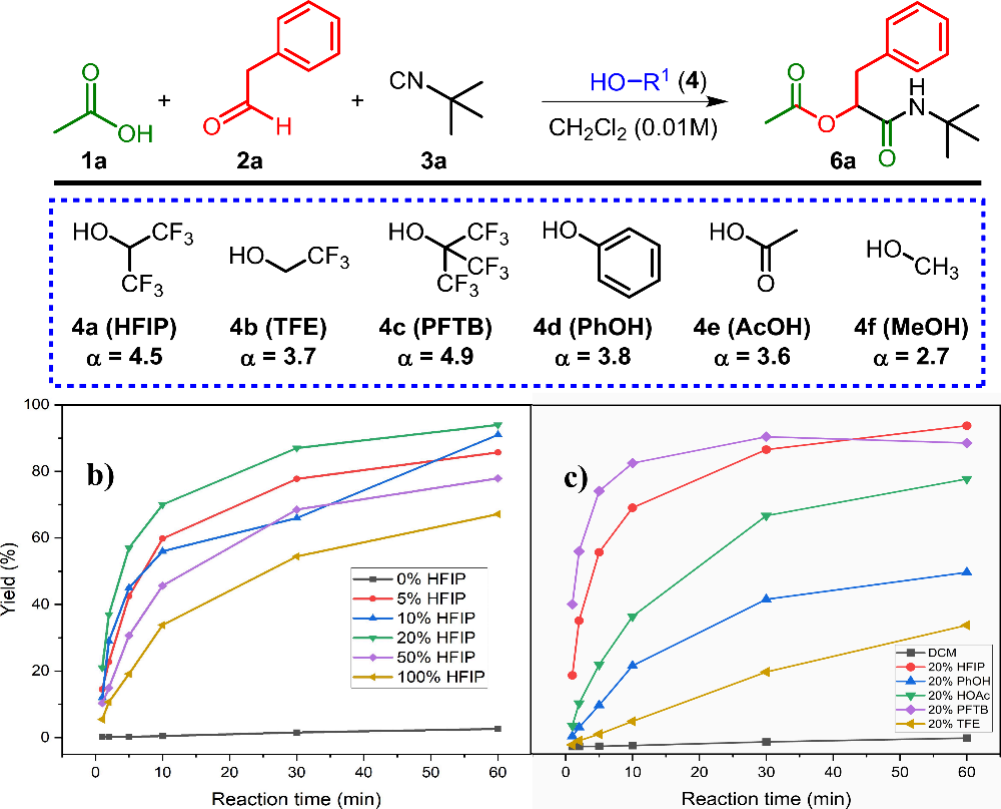
Rate enhancements observed with alcoholic solvents, along with their α values, as reported by Hunter.^18^ (a) Standard conditions: acetic acid (0.10 mmol), phenyl acetaldehyde (0.10 mmol), and *tert*-butyl isocyanide (0.10 mmol) in DCM mixtures of alcohols at 0.01 M concentration. Product formation was monitored over time by LC-MS. (b) Kinetic profile HFIP concentrations. (c) Kinetic profile HBD solvents.

We began our investigations with acetic acid (**1a**), phenylacetaldehyde (**2a**), and *tert*-butyl isocyanide (**3a**) as benchmark substrates (Figure 2). Solvent HBD properties were based on HBD constants (α), as reported by Hunter.^18^ Given the high α value of HFIP (4.5), we first tested the reaction in HFIP at 0.01 M. For comparison, we ran the reaction in CH_2_Cl_2_ at the same concentration. We observed a significant increase in reaction rate in HFIP, as monitored by HPLC analysis using biphenyl as an internal standard. Intrigued by this result, we tested HFIP:CH_2_Cl_2_ mixtures (5, 10, 20, and 50% v/v HFIP) (Figure 2B); finding 20% HFIP gave the fastest reaction rate. Next, we examined alcohols with varying HBD properties (**4a**–**4f**) (Figure 2). As expected, weaker HBD solvent TFE (**4b**, α = 3.9) showed slower reaction rates, while stronger HBD solvent perfluoro-*tert*-butanol (PFTB (**4c**), α = 4.9) increased the rate, although slight decomposition occurred with PFTB. Notably, TFE, typically used to suppress Passerini reactions when targeting Ugi 4CR reactions, paradoxically accelerated the P-3CR. Next, phenol, which has comparable acidity to HFIP (p*K*_a_ = 10.0 vs 9.3), while having a comparable α value to TFE, also enhanced the reaction rate, although less than HFIP. This supports our hypothesis that strong HBD solvents accelerate the Passerini reaction, even when competing in the rate-limiting step. Similarly, when acetic acid was used both as the acid component and as the cosolvent, it indeed increased the reaction rate, although to a lesser extent than HFIP. Despite the higher acidity of acetic acid (p*K*_a_ = 4.8) compared to HFIP, it remains the stronger HBD solvent. Finally, we confirmed that MeOH inhibited the P-3CR, with no product formation observed.

**Figure 3:**
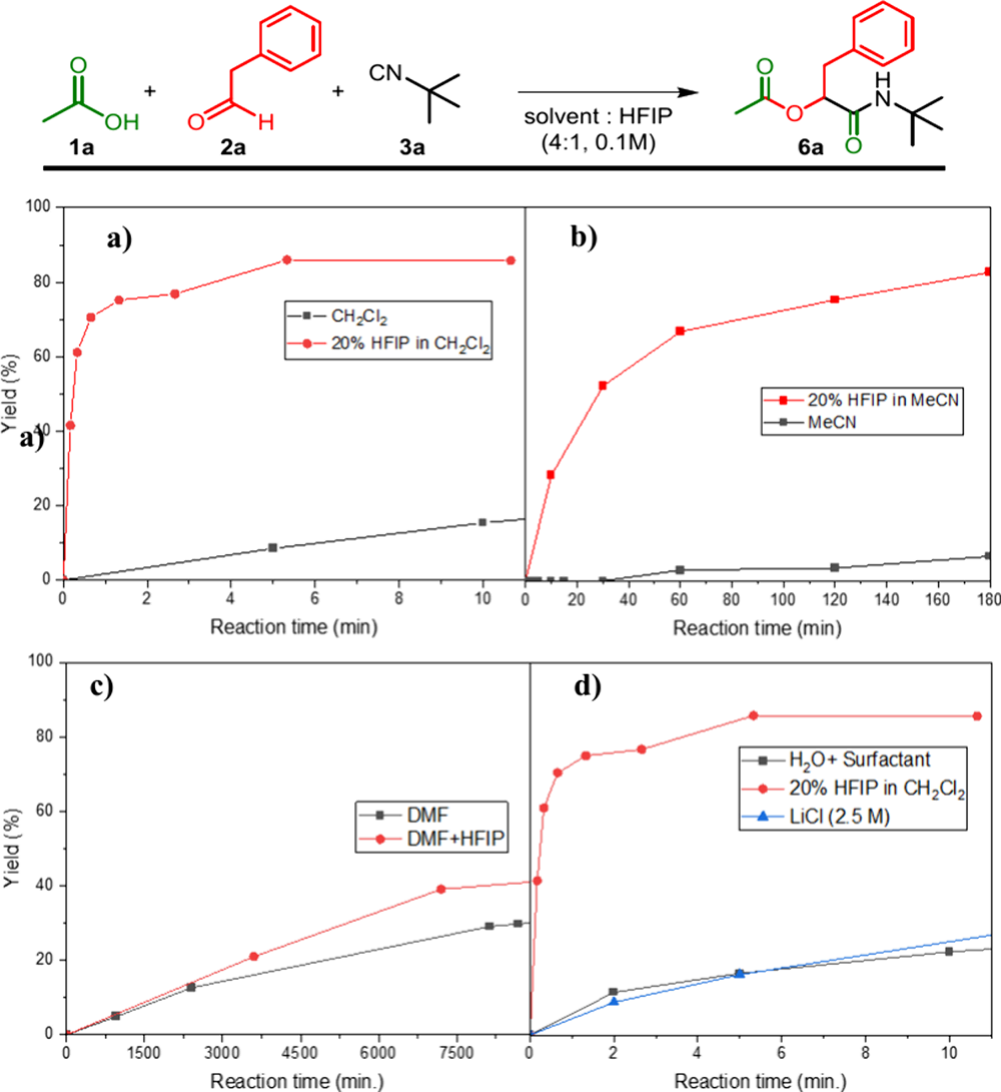
Representative plots of reaction progress in different solvents. (a) CH_2_Cl_2_. (b) MeCN. (c) DMF. (d) LiCl solution and Triton X-100 in H_2_O.

To explore the synthetic utility of HFIP as a cosolvent, we tested its effect in other organic solvents at a constant concentration of 0.1 M (Figure 3). A 20% v/v HFIP mixture in CH_2_Cl_2_ led to complete conversion within minutes (Figure 3a), compared to ∼50% conversion after 2 h without HFIP (Figure S4a), consistent with conventional Passerini reaction times. Similarly, HFIP significantly enhanced the reaction in chloroform (Figure S5a). In solvents like MeCN (Figure 3b), TBME (Figure S8a), and THF ((Figure S9a), reaction rates drastically dropped without HFIP, yielding only trace amounts after 3 h, likely due to higher hydrogen bond accepting (HBA) constants (β).^18^ However, HFIP as a cosolvent drastically accelerates the reaction, nearly achieving full conversion in 3 h. When moving to DMF, an even stronger HBA solvent (β value amides = 8.3), the low rate without HFIP (∼20% yield in 3 days), did not improve with the addition of HFIP. In contrast, 20% HFIP in MeOH showed moderate improvement, yielding 20% conversion after 4 h (Figure S10a).

In 2004, Pirrung and Sarma reported an 18-fold rate enhancement in the Passerini reaction using water as a solvent, with an even faster reaction observed in a 2.5 M aqueous LiCl solution.^10a,19^ Moreover, Paprocki et al. also improved yields by using surfactants like Triton X-100 in water.^10f^ Therefore, we compared the Passerini reaction in 20% v/v HFIP in CH_2_Cl_2_ with reactions in 2.5 M aqueous LiCl and Triton X-100 solutions at 0.1 M (Figure 3d). Surprisingly, the HFIP:CH_2_Cl_2_ mixture resulted in significantly faster reactions than both aqueous mixtures.

To establish the difference in rate of the P-3CR performed in a certain solvent and in its corresponding HFIP mixture, we determined the rate constants (Table 1). Since the Passerini reaction (with equimolar reagents) follows a third-order rate law, we used the analytic solution of the mass balance and determined the rates by plotting^19^ (see the Supporting Information S2.4 and S2.5 for details). The most substantial rate enhancement due to the addition of HFIP was observed in chloroform (i.e., 382 times faster compared to chloroform alone).

**Table 1:** Rate Constants Based on Third-Order Rate Equation for Solvents with and without HFIP as Cosolvent^a^

**solvent**	*k* **(no HFIP)** (mol^–2^· L^2^· s^–1^)	*k* **(20% HFIP)** (mol^–2^· L^2^· s^–1^)	*k* **(HFIP)/***k* (no HFIP)
CH_2_Cl_2_	2.60 × 10^–2^	7.20	276
CHCl_3_	2.52 × 10^–2^	9.66	382
MeCN	6.44 × 10^–3^	0.14	22
EtOAc	1.83 × 10^–3^	6.88 × 10^–2^	38
TBME	1.11 × 10^–3^	7.79 × 10^–2^	70
THF	1.12 × 10^–4^	6.10 × 10^–3^	54
MeOH	2.31 × 10^–4^	2.44 × 10^–3^	11
DMF	9.40 × 10^–5^	1.24 × 10^–4^	1.3
LiCl (2.5M)	3.10 × 10^–2^		

a See the Supporting Information for kinetic plots.

**Scheme 1:**
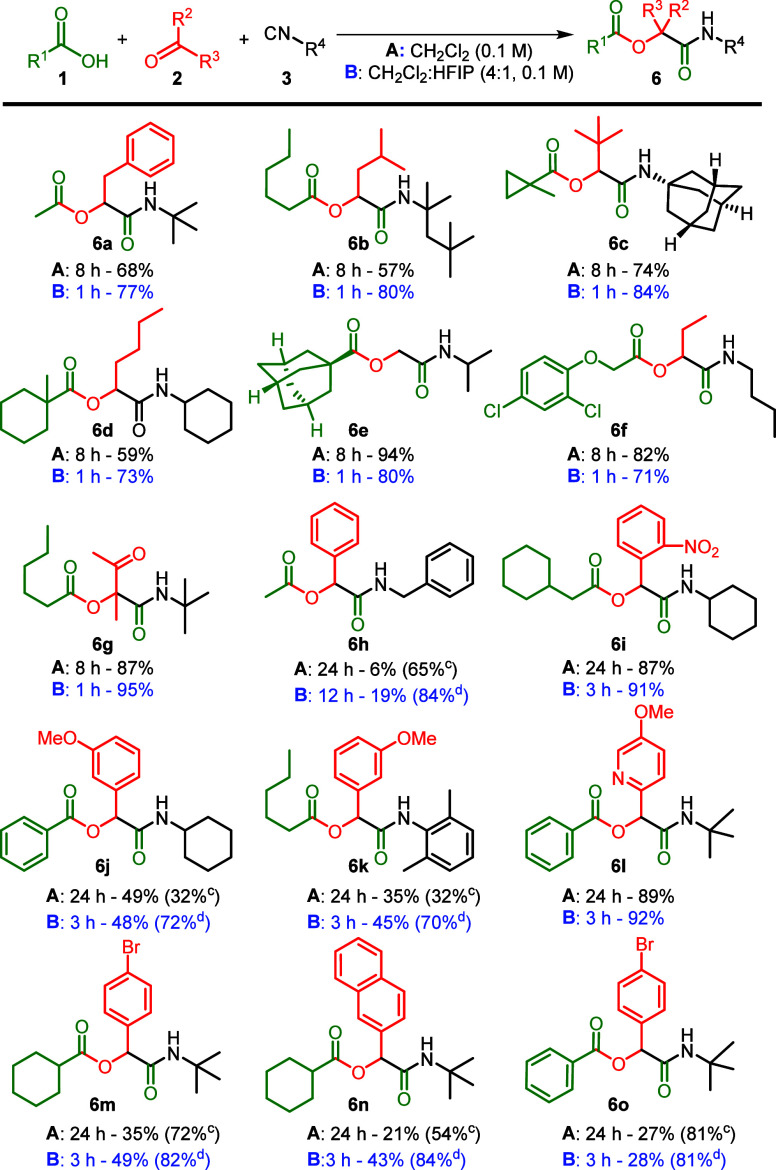
Scope of the Passerini Reaction in CH_2_Cl_2_ and CH_2_Cl_2_:HFIP (4:1) (a) Standard conditions: carboxylic acid (1.5 mmol), aldehyde/ketone (1.0 mmol), isocyanide (1.2 mmol) in A: CH_2_Cl_2_ (0.1 M) and B: CH_2_Cl_2_:HFIP (4:1, 0.1 M). (b) Reaction times and isolated yields. (c) Reaction is performed in CH_2_Cl_2_ (1 M) for 24 h. (d) Reaction is performed in CH_2_Cl_2_:HFIP (4:1, 1 M) for 12 h.

After establishing the solvent parameters, we aimed to investigate the effect of the rate enhancement on the yield of the reaction. We investigated various starting components and carried out the reaction both with and without HFIP (Scheme 1). To standardize the reaction conditions, we performed the reaction for 1 h in the presence of 20% v/v HFIP and for 8 h without HFIP. As evident in our benchmark reaction toward α-acyloxyamide **6a**, a slight increase in isolated yield was observed upon the addition of HFIP. Similarly, in the formation of α-acyloxyamides **6b**–**d**, we also observed a slight increase in yield. When moving to α-acyloxyamides **6e** and **6f**, a slight decrease in yield was noticeable, which might be attributed to slight decomposition caused by HFIP. For aromatic aldehydes, we extended the reaction times under both conditions, with and without HFIP (respectively 3 and 24 h), due to the lower electrophilicity. It became evident that electron-withdrawing groups significantly enhanced the yield under both conditions.

**Scheme 2:**
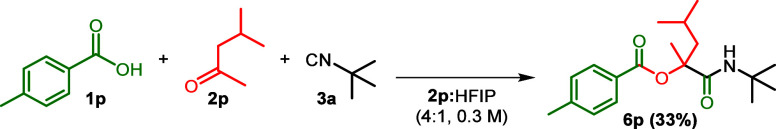
Passerini Reaction of Sterically Hindered Ketone **2p**

For aromatic aldehydes (compounds **6h**–**6o**), we observed low conversion rates and subsequently low yields at the standard 0.1 M concentration in both DCM and HFIP/DCM. We found that increasing the concentration from 0.1 to 1 M significantly enhanced product formation (see Section S3.2). As expected, no difference in yield was observed when transforming from aliphatic to aromatic substituents in the isocyanide (acyloxyamides **6j** vs **6k**) or in the acid components (acyloxyamides **6m** vs **6n**–**6o**). Activated ketones efficiently produced the desired Passerini product, yielding 95 and 87% under conditions with and without the addition of HFIP, respectively. However, alkyl substituted ketones generally exhibit low conversions due to their lower electrophilicity, even when extending the reaction time to multiple days. Therefore, to further explore the limits of this rate enhancement caused by HFIP, we investigated the Passerini reaction involving *p*-toluic acid (**1p**), methyl isobutyl ketone (**2p**), and *tert*-butyl isocyanide (**3a**). Jenner demonstrated that this reaction produced only a 5% yield after 16.5 h when the ketone was used as the solvent.^15^ Although increasing the pressure to 3 kbar favored this reaction, resulting in 39% yield, it might not be a feasible reaction setup for every lab. Therefore, we investigated whether 20% v/v HFIP with sterically hindered ketone **2p** as the solvent would enhance the yield. After some optimization, we found that performing the reaction at a concentration of 0.3 M in ketone **2p** with 20% v/v HFIP for 24 h resulted in a 33% isolated yield (Scheme 2). Higher yields could be achieved by extending the reaction time further.

**Figure 4:**
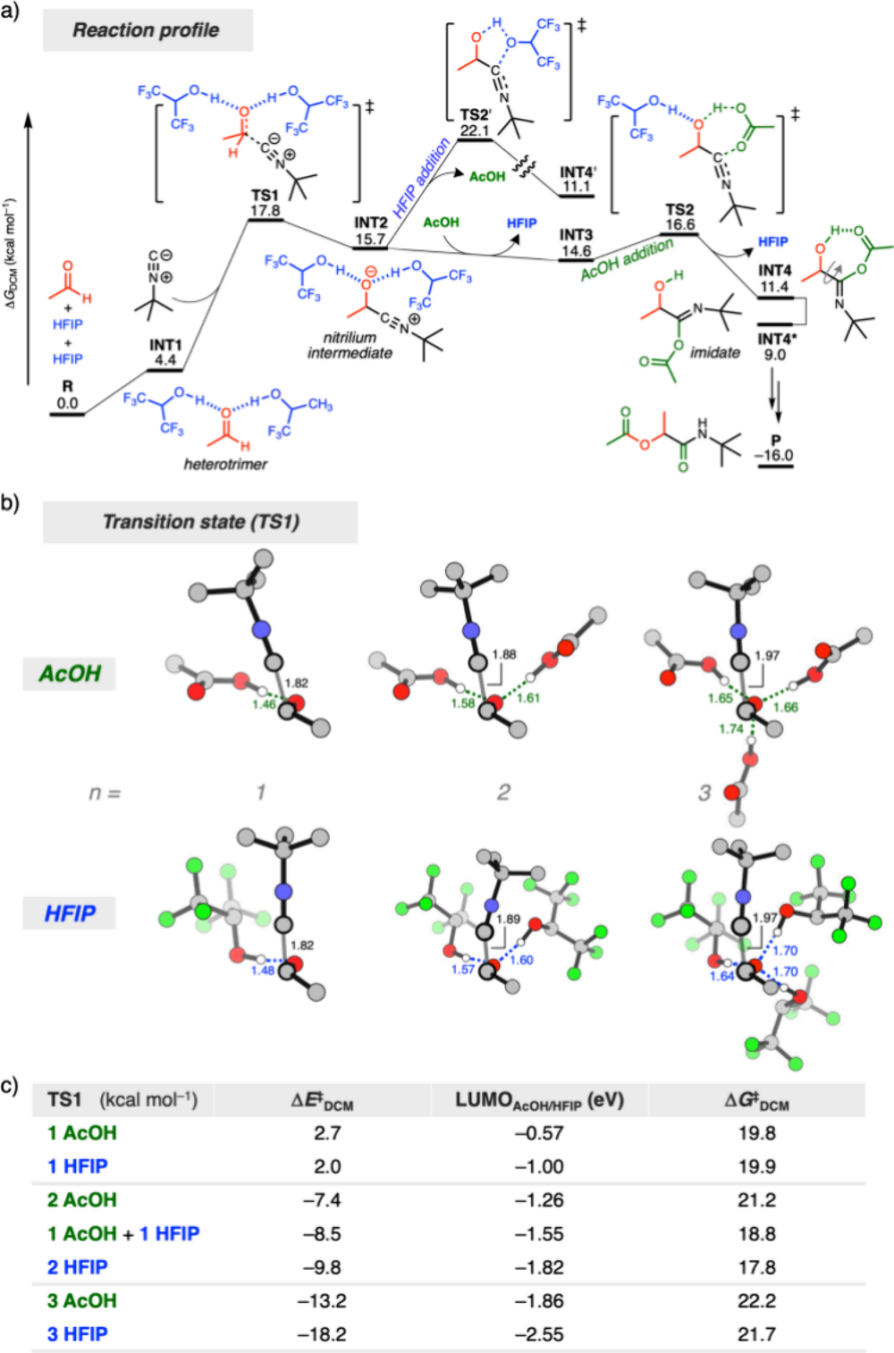
Mechanistic effect of HFIP as a cosolvent using DFT calculations. (a) Reaction profile (Δ*G*_DCM_ in kcal mol^–1^) of a Passerini-type reaction between acetaldehyde, *tert*-butyl isocyanide, AcOH, and HFIP in dichloromethane. (b) Transition state structures for the nucleophilic addition of *tert*-butyl isocyanide to acetaldehyde (**TS1**) with key bond lengths (in Å). (c) Reaction barriers (Δ*E*^‡^_DCM_ and Δ*G*^‡^_DCM_) and the corresponding LUMO energies (eV) of the AcOH/HFIP molecules in the transition state geometry of **TS1**. All nonpolar hydrogens are omitted for clarity reasons. Atom colors: carbon (gray), fluorine (green), hydrogen (white), nitrogen (blue), and oxygen (red). Computed at COSMO(DCM)-ZORA-BLYP-BJ(D3)/TZ2P.

Having established the role of HFIP as a cosolvent in the Passerini reaction, we explored its mechanistic effect using DFT calculations (see the SI for details). Figure 4a shows the computed reaction profile of model substrates acetaldehyde, *tert*-butyl isocyanide, HFIP, and AcOH with the energies relative to reactants. As reported by Morokuma et al.,^16^ the rate-determining step is the isocyanide addition (**TS1**). Initially, the barrier for isocyanide addition was computed as Δ*E*^‡^_DCM_ = +2.7 kcal mol^–1^, which lowered systematically with one, two, and three AcOH molecules (Δ*E*^‡^_DCM_ = −7.4 to −13.2 kcal mol^–1^; Figure S14a,b). Similarly, introducing HFIP molecules reduced the barrier from Δ*E*^‡^_DCM_ = +2.0 to −9.8 to −18.2 kcal mol^–1^ (Figure S14c,d). HFIP showed a steeper decrease compared to AcOH (ΔΔ*E*^‡^_DCM,HFIP_ = −20.2 kcal mol^–1^ vs ΔΔ*E*^‡^_DCM,AcOH_ = −15.9 kcal mol^–1^), due to its stronger interaction with aldehyde, consistent with Hunter’s work, leading to more efficient isocyanide addition.^18,20^

Notably, the largest barrier reduction occurred with the first few HFIP or AcOH molecules, followed by a leveling-off effect, attributed to saturation in aldehyde–HFIP/AcOH interactions.^21^ The barrier-lowering effect was less pronounced in Δ*G*_DCM_ energies due to entropic penalties, when adding multiple HFIP molecules, as **TS1** shifted from Δ*G*^‡^_DCM_ = +19.9 to +17.8 to +21.7 kcal mol^–1^ (see Figure S15 for the reaction profile of Figure 4 in Δ*E*_DCM_). Note that the computed rate-determining transition states are consistent with third-order kinetics, involving the aldehyde, isocyanide, and the hydrogen bonding component (HFIP/AcOH). After isocyanide addition, AcOH attacked the nitrilium intermediate (**TS2**: Δ*G*^‡^_DCM_ = +16.6 kcal mol^–1^), while attack of HFIP has a higher barrier (**TS2’**: Δ*G*^‡^_DCM_ = +22.1 kcal mol^–1^). The subsequent Mumm rearrangement then yields the final product (**P**: Δ*G*_DCM_ = – 16.0 kcal mol^–1^).

In conclusion, we have provided new insights into the traditional Passerini reaction, demonstrating that strong HBD solvents accelerate reaction kinetics, contrary to the common belief that they slow the reaction due to competition with the carboxylic acid in the rate-determining step. HFIP, in particular, exhibited the fastest kinetics as a cosolvent (20% v/v) in apolar solvents, with up to a 382-fold increase observed in chloroform. This rate enhancement was limited to aprotic, nonbasic solvents (Table 1) and was consistent across a broad substrate scope, without affecting overall yield (Scheme 1). This makes it especially valuable for high-throughput applications or substrates with slow reaction rates (Scheme 2). Finally, computational studies confirmed that the rate-limiting aldehyde addition is most favorable when two HFIP molecules participate in hydrogen bonding (Figure 4), validating both our hypothesis and experimental results.

## Experimental Section

### General Information

Commercially available reagents, including substrates carboxylic acids **1**, aldehydes **2**, and isocyanides **3**, were purchased from Sigma-Aldrich, Fisher Scientific, Strem Chemicals, TCI Chemicals, Activate Scientific, or Fluorochem and were used as purchased unless mentioned otherwise. Solvents were purchased from VWR Chemicals or Sigma-Aldrich and used without purification, unless stated otherwise. Reagent-grade solvent was used for the optimization. Thin layer chromatography (TLC) was performed using plates from Merck (SiO_2_, Kieselgel 60 F254 neutral, on aluminum with fluorescence indicator), and compounds were visualized by UV detection (254 nm), KMnO_4_, and/or Hanessian’s stain. Liquid chromatography–mass spectrometry (LC-MS) analysis was performed on a Shimadzu Nexera 2 UHPLC system equipped with a Shimadzu LC-30AD pump, an SPD-M30A photodiode array detector, and a LC-MS-2020 single quadrupole detector. The system was run on Milli-Q water and LC-MS grade acetonitrile both modified with 0.1% formic acid. A Waters XSelect CSH C18 column (3.0 mm × 75 mm with a particle size of 3.5 μm) was used operating at 30 °C. The method was set up with a gradient of 5% acetonitrile in water for 2 min and an increase to 95% acetonitrile over 12 min and 1 min at 95% followed by flushing back to 5% acetonitrile. Flash column chromatography was performed by employing silica (200–300 mesh) as support and *n*-heptane/ethyl acetate. NMR spectra were recorded on a Brüker Avance 300 using the residual CDCl_3_ as internal reference (^1^H: δ 7.26 ppm, ^13^C: δ 77.16 ppm). Chemical shifts (δ) are given in ppm, and coupling constants (*J*) are quoted in hertz (Hz). Resonances are described as s (singlet), d (doublet), t (triplet), q (quartet), br (broad singlet), and m (multiplet) or combinations thereof. Ultrahigh-resolution mass-spectrometer Bruker solariX XR FT-ICR-MS was used for accurate mass measurements. Samples were ionized by electrospray ionization (ESI) in positive ion mode. NMR data were processed with Mestrenova version 12.

### General Procedure for Passerini Reaction

To a solution of isocyanide **3** (1.2 mmol, 1.2 equiv) and acid **1** (1.5 mmol, 1.5 equiv) in solvent (10 mL, 0.1 M), the carbonyl compound **2** (1.0, 1.0 equiv) was added and the reaction was allowed to stir for a specific time (see the SI for details). Then, the reaction was washed two times with 10 mL of NaHCO_3_ and one time with 10 mL of brine. The organic layers were dried over anhydrous Na_2_SO_4_. After the removal of the solvent, product **6** was purified through chromatography.

#### 1-(*tert*-Butylamino)-1-oxo-3-phenylpropan-2-yl Acetate (**6a**)

Prepared from *tert*-butyl isocyanide (136 μL, 1.2 mmol, 1.2 equiv), acetic acid (85.8 μL, 1.5 mmol, 1.5 equiv), and phenyl acetic aldehyde (117 μL, 1 mmol, 1 equiv) according to the general procedure and isolated as a white solid. Yield DCM: 179 mg, 0.68 mmol, 68%; Yield DCM:HFIP (80:20): 203 mg, 0.77 mmol, 77%. Column chromatography: silica, 20% EtOAc/heptane. ^1^H NMR (300 MHz, CDCl_3_): 7.30–7.16 (m, 5H), 5.65 (s, 1H), 5.22 (t, *J* = 5.9 Hz, 1H), 3.15–3.13 (dd, *J*_1_ = 5.9 Hz, *J*_2_ = 3.0 Hz, 2H), 2.06 (s, 3H), 1.25 (s, 9H). ^13^C{^1^H} NMR (75 MHz, CDCl_3_): 169.3, 167.8, 135.9, 129.7, 128.2, 126.8, 74.5, 51.1, 37.6, 28.5, 20.9. HRMS (ESI-TOF) *m*/*z*: [M + H]^+^ calcd for [C_15_H_21_NO_3_+H]^+^:264.1594, found: 264.1600.

#### 4-Methyl-1-oxo-1-((2,4,4-trimethylpentan-2-yl)amino)pentan-2-yl Hexanoate (**6b**)

Prepared from 2-isocyano-2,4,4-trimethylpentane (201 μL, 1.2 mmol, 1.2 equiv), hexanoic acid (188 μL, 1.5 mmol, 1.5 equiv), and isovaleraldehyde (107 μL, 1 mmol, 1 equiv) according to the general procedure and isolated as a white solid. Yield DCM: 195 mg, 0.57 mmol, 57%; yield DCM:HFIP (80:20): 273 mg, 0.80 mmol, 80%. Column chromatography: silica, 20% EtOAc/heptane. ^1^H NMR (300 MHz, CDCl_3_): 5.82 (s, 1H), 5.03 (t, *J* = 6.4 Hz, 1H), 2.31–2.25 (m, 2H), 1.67–1.52 (m, 7H), 1.32 (d, *J* = 2.2 Hz, 6H), 1.27–1.23 (m, 4H), 0.91 (s, 9H), 0.86–0.80 (m, 9H). ^13^C{^1^H} NMR (75 MHz, CDCl_3_): 172.3, 169.0, 72.9, 55.1, 52.1, 40.8, 34.3, 31.6, 31.5, 31.2, 28.9, 28.8, 24.6, 24.5, 23.1, 22.3, 21.7, 13.8. HRMS (ESI-TOF) *m*/*z*: [M + H]^+^ calcd for [C_20_H_39_NO_3_+H]^+^: 342.3003, found: 342.3015.

#### 1-(((1*S*,3*s*)-Adamantan-1-yl)amino)-3,3-dimethyl-1-oxobutan-2-yl 1-Methylcyclopropane-1-carboxylate (**6c**)

Prepared from adamantyl isocyanide (194 mg, 1.2 mmol, 1.2 equiv), 1-methylcyclohexane-1-carboxylic acid (150 mg, 1.5 mmol, 1.5 equiv), and *tert*-butyl aldehyde (109 μL, 1 mmol, 1 equiv) according to the general procedure and isolated as a white solid. Yield DCM: 257 mg, 0.74 mmol, 74%; yield DCM:HFIP (80:20): 292 mg, 0.84 mmol, 84%. Column chromatography: silica, 10% EtOAc/heptane. ^1^H NMR (300 MHz, CDCl_3_): 5.42 (s, 1H), 4.61 (s, 1H), 2.06 (s, 3H), 1.98 (s, 6H), 1.66 (s, 6H), 1.34 (s, 3H), 1,26–1,24 (m, 2H), 0.99 (s, 9H), 0.76–0.73 (m, 2H). ^13^C{^1^H}NMR (75 MHz, CDCl_3_) δ 174.5, 167.4, 80.9, 51.9, 41.6, 36.4, 34.3, 29.5, 26.4, 19.5, 18.7, 17.1, 16.9. HRMS (ESI-TOF) *m*/*z*: [M + H]^+^ calcd for [C_21_H_33_NO_3_+H]^+^: 348.2533, found: 348.2548.

#### 1-(Cyclohexylamino)-1-oxohexan-2-yl 1-Methylcyclohexane-1-carboxylate (**6d**)

Prepared from cyclohexyl isocyanide (149 μL, 1.2 mmol, 1.2 equiv), 1-methylcyclohexane-1-carboxylic acid (213 mg, 1.5 mmol, 1.5 equiv), and pentanal (106 μL, 1 mmol, 1 equiv) according to the general procedure and isolated as a white solid. Yield DCM: 199 mg, 0.59 mmol, 59%; yield DCM:HFIP (80:20): 246 mg, 0.73 mmol, 73%. Column chromatography: silica, 10% EtOAc/heptane. ^1^H NMR (300 MHz, CDCl_3_): 5.87 (d, *J* = 8.3 Hz, 1H), 5.14 (dd, *J*_1_= 7.1 Hz, *J*_2_= 4.8 Hz, 1H), 3.80–3.68 (m, 1H), 2.06–2.00 (m, 2H), 1.90–1.76 (m, 4H), 1.68–1.49 (m, 6H), 1.41–1.23 (m, 10H), 1.18 (s, 3H), 1.17–1.05 (m, 3H), 0.87–0.83 (m, 3H). ^13^C{^1^H} NMR (75 MHz, CDCl_3_): 181.9, 176.2, 169.3, 73.6, 47.8, 43.4, 35.6, 35.6, 33.1, 33.0, 31.6, 26.9, 25.7, 25.5, 24.7, 24.7, 23.4, 23.2, 22.4, 13.9. HRMS (ESI-TOF) *m*/*z*: [M + H]^+^ calcd for [C_20_H_35_NO_3_+H]^+^: 338.2690, found: 338.2683.

#### 2-(Isopropylamino)-2-oxoethyl (3*r*,5*r*,7*r*)-adamantane-1-carboxylate (**6e**)

Prepared from isopropyl isocyanide (113 μL, 1.2 mmol, 1.2 equiv), adamantane-1-carboxilyc acid (270 mg, 1.5 mmol, 1.5 equiv), and formaldehyde (74.5 μL, 1 mmol, 1 equiv) according to the general procedure and isolated as a white solid.

Yield DCM: 263 mg, 0.94 mmol, 94%; yield DCM:HFIP (80:20): 224 mg, 0.80 mmol, 80%. Column chromatography: silica, 20% EtOAc/heptane. ^1^H NMR (300 MHz, CDCl_3_): 5.90 (d, *J* = 5.9 Hz, 1H), 4.39 (s, 2H), 4.04–3.92 (m, 1H), 1.95–1.92 (m, 3H), 1.82 (d, *J* = 3.2 Hz, 6H), 1.68–1.57 (m, 6H), 1.07 (d, *J* = 6.6 Hz, 6H). ^13^C{^1^H}NMR (75 MHz, CDCl_3_): 175.9, 166.3, 62.4, 41.0, 40.6, 38.7, 36.2, 27.7, 22.5. HRMS (ESI-TOF) *m*/*z*: [M + H]^+^ calcd for [C_16_H_25_NO_3_+H]^+^: 280.1907, found: 280.1923.

#### 1-(Butylamino)-1-oxobutan-2-yl 2-(2,4-Dichlorophenoxy)acetate (**6f**)

Prepared from butyl isocyanide (126 μL, 1.2 mmol, 1.2 equiv), 2-(2,4-dichlorophenoxy)acetic acid (332 mg, 1.5 mmol, 1.5 equiv), and propanal (72.1 μL, 1 mmol, 1 equiv) according to the general procedure and isolated as a white solid. Yield DCM: 297 mg, 0.82 mmol, 82%; yield DCM:HFIP (80:20): 257 mg, 0.71 mmol, 71%. Column chromatography: silica, 20% EtOAc/heptane. ^1^H NMR (300 MHz, CDCl_3_): 7.33 (d, 1H, *J* = 2.3 Hz), 7.12 (dd, *J*_1_= 8.8 Hz, *J*_2_ = 2.5 Hz, 1H), 6.78 (d, *J* = 8.8 Hz, 1H), 6.32 (t, *J* = 6.0 Hz, 1H), 5.14 (t, *J* = 5.4 Hz, 1H), 4.76 (d, *J* = 16.0 Hz, 1H), 4.69 (d, *J* = 16.1 Hz, 1H), 3.25–3.04 (m, 2H), 1.89–1.76 (m, 2H), 1.43 – 1.31 (m, 2H), 1.29 – 1.16 (m, 2H), 0.86–0.79 (m, 6H). ^13^C{^1^H} NMR (75 MHz, CDCl_3_): 168.8, 166.9, 152.0, 130.3, 127.7, 127.2, 123.8, 114.3, 75.9, 66.1, 39.0, 31.5, 25.0, 19.9, 13.6, 8.7. HRMS (ESI-TOF) *m*/*z*: [M + H]^+^ calcd for [C_16_H_21_Cl_2_NO_4_+H]^+^: 362.0920, found: 362.0939.

#### 1-(*tert*-Butylamino)-2-methyl-1,3-dioxobutan-2-yl Hexanoate (**6g**)

Prepared from *tert*-butyl isocyanide (136 μL, 1.2 mmol, 1.2 equiv), hexanoic acid (188 μL, 1.5 mmol, 1.5 equiv), and 2,3-butanedione (87.0 μL, 1 mmol, 1 equiv) according to the general procedure and isolated as a colorless oil without the need of an additional purification step. Yield DCM: 248 mg, 0.87 mmol, 87%; yield DCM:HFIP (80:20): 271 mg, 0.95 mmol, 95%. ^1^H NMR (300 MHz, CDCl_3_) δ 6.74 (s, 1H), 2.28 (td, *J* = 7.5, 3.1 Hz, 2H), 2.05 (s, 3H), 1.55 (s, 3H), 1.49 (m, 2H), 1.18 (s, 13H), 0.77 – 0.69 (m, 3H). ^13^C{^1^H} NMR (75 MHz, CDCl_3_): 203.7, 172.0, 166.2, 84.7, 51.1, 33.8, 30.9, 28.2, 25.1, 24.1, 22.1, 20.9, 13.6. HRMS (ESI-TOF) *m*/*z*: [M + H]^+^ Calcd for [C_15_H_27_NO_4_+H]^+^: 286.2013, found: 286.2031.

#### 2-(Benzylamino)-2-oxo-1-phenylethyl Acetate (**6h**)

Prepared from benzyl isocyanide (146 μL, 1.2 mmol, 1.2 equiv), acetic acid (85.8 μL, 1.5 mmol, 1.5 equiv), and benzaldehyde (102 μL, 1 mmol, 1 equiv) according to the general procedure at concentration (1 M) and isolated as a white solid. Yield DCM: 184 mg, 0.65 mmol, 65%; yield DCM:HFIP (80:20): 238 mg, 0.84 mmol, 84%. Column chromatography: silica, DCM then 10% MeOH in DCM. ^1^H NMR (300 MHz, CDCl_3_): 7.44–7.15 (m, 10H), 6.61 (bs, 1H), 6.08 (s, 1H), 4.42 (t, *J* = 5.4 Hz, 2H), 2.11 (s, 3H). ^13^C{^1^H} NMR (75 MHz, CDCl_3_): 169.4, 168.4, 137.8, 135.6, 129.1, 128.8, 128.8, 127.7, 127.7, 127.5, 75.6, 43.4, 21.1. HRMS (ESI-TOF) *m*/*z*: [M + H]^+^ Calcd for [C_17_H_17_NO_3_+H]^+^: 284.1281, found: 284.1288.

#### 2-(Cyclohexylamino)-1-(2-nitrophenyl)-2-oxoethyl 2-Cyclohexylacetate (**6i**)

Prepared from cyclohexyl isocyanide (149 μL, 1.2 mmol, 1.2 equiv), 2-cyclohexylacetic acid (212 μL, 1.5 mmol, 1.5 equiv), and 2-nitrobenzaldehyde (151 mg, 1 mmol, 1 equiv) according to the general procedure and isolated as a white solid. Yield DCM: 350 mg, 0.87 mmol, 87%; yield DCM:HFIP (80:20): 366 mg, 0.91 mmol, 91%. Column chromatography: silica, 40% EtOAc/heptane. ^1^H NMR (300 MHz, CDCl_3_): 7.91 (dd, *J*_1_ = 8.2 Hz, J_2_= 1.3 Hz, 1H), 7.69 (dd, *J*_1_ = 7.9 Hz, *J*_2_ = 1.5 Hz, 1H), 7.59 (td, *J*_1_ = 7.6 Hz, *J*_2_ = 1.3 Hz, 1H), 7.44 (td, *J*_1_ = 7.7 Hz, *J*_2_ = 1.5 Hz, 1H), 6.57 (s, 1H), 6.32 (d, *J* = 8.2 Hz, 1H), 3.70–3.64 (m, 1H), 2.26 (dd, *J*_1_ = 7.1 Hz, *J*_2_ = 1.6 Hz, 2H), 1.90–1.49 (m, 11H), 1.36–0.86 (m, 10H). ^13^C{^1^H} NMR (75 MHz, CDCl_3_): 171.5, 165.8, 148.1, 133.4, 130.9, 129.8, 129.4, 124.7, 70.9, 48.4, 41.6, 34.8, 32.8, 32.8, 32.6, 32.6, 25.9, 25.8, 25.8, 25.4, 24.5. HRMS (ESI-TOF) *m*/*z*: [M + H]^+^ calcd for [C_22_H_30_N_2_O_5_+H]^+^: 403.2227, found: 403.2267.

#### 2-(Cyclohexylamino)-1-(3-methoxyphenyl)-2-oxoethyl Benzoate (**6j**)

Prepared from cyclohexyl isocyanide (149 μL, 1.2 mmol, 1.2 equiv), benzoic acid (183 mg, 1.5 mmol, 1.5 equiv), and *m*-anisaldehyde (122 μL, 1 mmol, 1 equiv) according to the general procedure at concentration (1 M) and isolated as a white solid. Yield DCM: 117 mg, 0.32 mmol, 32%; yield DCM:HFIP (80:20): 264 mg, 0.72 mmol, 72%. Column chromatography: silica, 30% EtOAc/heptane. ^1^H NMR (300 MHz, CDCl_3_): 8.10–8.08 (m, 2H), 7.60 (t, *J* = 7.4 Hz, 1H), 7.47 (t, *J* = 7.7 Hz, 2H), 7.30 (t, *J* = 7.9 Hz, 1H), 7.12–7.08 (m, 2H), 6.89 (dd, *J*_1_ = 8.1 Hz, *J*_2_ = 2.4 Hz, 1H), 6.27 (s, 1H), 6.07 (d, *J* = 8.2 Hz, 1H), 3.81 (m, 4H), 2.00 – 1.84 (m, 2H), 1.75 – 1.53 (m, 3H), 1.35 (m, 2H), 1.23 – 1.05 (m, 3H). ^13^C{^1^H} NMR (75 MHz, CDCl_3_): 167.3, 165.0, 159.9, 137.3, 133.7, 129.9, 129.9, 129.4, 128.7, 119.7, 114.5, 113.2, 75.9, 55.4, 48.3, 33.0, 32.9, 25.5, 24.8, 24.8. HRMS (ESI-TOF) *m*/*z*: [M + H]^+^ calcd for [C_22_H_25_NO_4_+H]^+^ 368.1856, found: 368.1874.

#### 2-((2,6-Dimethylphenyl)amino)-1-(3-methoxyphenyl)-2-oxoethyl Hexanoate (**6k**)

Prepared from 2-isocyano-1,3-dimethylbenzene (157 mg, 1.2 mmol, 1.2 equiv), hexanoic acid (188 μL, 1.5 mmol, 1.5 equiv), and *m*-anisaldehyde (122 μL, 1 mmol, 1 equiv) according to the general procedure at concentration (1 M) and isolated as a pale-yellow solid. Yield DCM: 123 mg, 0.32 mmol, 32%; yield DCM:HFIP (80:20): 268 mg, 0.70 mmol, 70%. Column chromatography: silica, 20% EtOAc/heptane. ^1^H NMR (300 MHz, CDCl_3_) δ 7.34 (d, *J* = 2.2 Hz, 1H), 7.30 (d, *J* = 7.9 Hz, 1H), 7.15 – 7.00 (m, 5H), 6.94 – 6.87 (m, 1H), 6.18 (s, 1H), 3.81 (s, 3H), 2.49 (td, *J* = 7.4, 1.2 Hz, 2H), 2.10 (s, 6H), 1.78–1.63 (m, 2H), 1.33 (h, *J* = 3.7 Hz, 4H), 0.94–0.83 (m, 3H). ^13^C{^1^H} NMR (75 MHz, CDCl_3_) δ 172.3, 166.8, 160.0, 137.0, 135.6, 132.8, 130.0, 128.3, 127.6, 119.5, 115.0, 112.8, 75.7, 55.4, 34.3, 31.3, 24.7, 22.4, 18.3, 14.0. HRMS (ESI-TOF) *m*/*z*: [M + H]^+^ calcd for [C_23_H_29_NO_4_+H]^+^: 384.2169, found 384.2173.

#### 2-(*tert*-Butylamino)-1-(5-methoxypyridin-2-yl)-2-oxoethyl Benzoate (**6l**)

Prepared from *tert*-butyl isocyanide (136 μL, 1.2 mmol, 1.2 equiv), benzoic acid (183 mg, 1.5 mmol, 1.5 equiv), and 5-methoxypicolinaldehyde (137 mg, 1 mmol, 1 equiv) according to the general procedure and isolated as a colorless solid. Yield DCM: 305 mg, 0.89 mmol, 89%; yield DCM:HFIP (80:20): 315 mg, 0.92 mmol, 92%. Column chromatography: silica, 20% EtOAc/heptane. ^1^H NMR (300 MHz, CDCl_3_) δ 8.20–8.13 (m, 2H), 7.62–7.56 (m, 2H), 7.47 (dd, *J* = 8.3, 6.9 Hz, 2H), 7.20 (d, *J* = 7.3 Hz, 1H), 6.73 (s, 1H), 6.70 (s, 1H), 3.93 (s, 3H), 1.36 (s, 9H). ^13^C{^1^H} NMR (75 MHz, CDCl_3_) δ 166.0, 165.3, 163.7, 152.7, 139.7, 133.6, 130.3, 130.1, 128.7, 114.4, 111.0, 75.7, 53.5, 51.7, 28.9. HRMS (ESI-TOF) *m*/*z*: [M + H]^+^ calcd for [C_19_H_22_N_2_O_4_+H]^+^: 343.1652, found: 343.1653.

#### 1-(4-Bromophenyl)-2-(*tert*-butylamino)-2-oxoethyl Cyclohexanecarboxylate (**6m**)

Prepared from *tert*-butyl isocyanide (136 μL, 1.2 mmol, 1.2 equiv), cyclohexanecarboxylic acid (192 mg, 1.5 mmol, 1.5 equiv), and 4-bromobenzaldehyde (100 μL, 1 mmol, 1 equiv) according to the general procedure at concentration (1 M) and isolated as a colorless solid. Yield DCM: 284 mg, 0.72 mmol, 72%; yield DCM:HFIP (80:20): 324 mg, 0.82 mmol, 82%. Column chromatography: silica, 20% EtOAc/heptane. ^1^H NMR (300 MHz, CDCl_3_) δ 7.53–7.43 (m, 2H), 7.33–7.24 (m, 2H), 5.95 (s, 1H), 5.91 (s, 1H), 2.42 (tt, *J* = 11.2, 3.7 Hz, 1H), 1.94 (t, *J* = 14.4 Hz, 2H), 1.77 (m, 2H), 1.67 (m, 1H), 1.55–1.40 (m, 2H), 1.34–1.17 (m, 12H). ^13^C{^1^H} NMR (75 MHz, CDCl_3_) δ 173.9, 167.1, 135.4, 131.9, 129.0, 123.0, 74.6, 51.6, 43.0, 29.1, 28.9, 28.8, 25.7, 25.4, 25.3. HRMS (ESI-TOF) *m*/*z*: [M + H]^+^ calcd for [C_19_H_26_BrNO_3_+H]^+^: 396.1169, found: 396.1174.

#### 2-(*tert*-Butylamino)-1-(naphthalen-2-yl)-2-oxoethyl Cyclohexanecarboxylate (**6n**)

Prepared from *tert*-butyl isocyanide (136 μL, 1.2 mmol, 1.2 equiv), cyclohexanecarboxylic acid (192 mg, 1.5 mmol, 1.5 equiv), and 2-naphthaldehyde (96.4 μL, 1 mmol, 1 equiv) according to the general procedure at concentration (1 M) and isolated as a colorless solid. Yield DCM: 198 mg, 0.54 mmol, 54%; yield DCM:HFIP (80:20): 308 mg, 0.84 mmol, 84%. Column chromatography: silica, 30% EtOAc/heptane. ^1^H NMR (300 MHz, CDCl_3_) δ 7.90–7.89 (m, 1H), 7.84 (td, *J* = 5.7, 2.8 Hz, 3H), 7.53–7.47 (m, 3H), 6.15 (s, 1H), 5.99 (s, 1H), 2.51–2.41 (m, 1H), 2.04–1.92 (m, 2H), 1.82–1.74 (m, 2H), 1.58–1.20 (m, 15H). ^13^C {^1^H} NMR (75 MHz, CDCl_3_) δ 174.2, 167.6, 133.6, 133.5, 133.2, 128.7, 128.4, 127.8, 127.2, 126.6, 126.5, 124.6, 75.5, 51.6, 43.2, 29.2, 29.0, 28.8, 25.8, 25.5, 25.4. HRMS (ESI-TOF) *m*/*z*: [M + H]^+^ calcd for [C_23_H_29_NO_3_+H]^+^: 368.2220, found: 368.2221.

#### 1-(4-Bromophenyl)-2-(*tert*-butylamino)-2-oxoethyl Benzoate (**6o**)

Prepared from *tert*-butyl isocyanide (136 μL, 1.2 mmol, 1.2 equiv), benzoic acid (183 mg, 1.5 mmol, 1.5 equiv), and 4-bromobenzaldehyde (100 μL, 1 mmol, 1 equiv) according to the general procedure at concentration (1 M) and isolated as a colorless solid. Yield DCM: 310 mg, 0.81 mmol, 81%; yield DCM:HFIP (80:20): 310 mg, 0.81 mmol, 81%. Column chromatography: silica, 20% EtOAc/heptane. ^1^H NMR (300 MHz, CDCl_3_) δ 8.09–8.05 (m, 2H), 7.66–7.59 (m, 1H), 7.55–7.46 (m, 4H), 7.43–7.37 (m, 2H), 6.16 (s, 1H), 6.03 (s, 1H), 1.37 (s, 9H). ^13^C{^1^H} NMR (75 MHz, CDCl_3_) δ 167.0, 164.9, 135.2, 133.9, 132.1, 129.9, 129.3, 129.2, 128.9, 123.2, 75.4, 51.8, 28.8. HRMS (ESI-TOF) *m*/*z*: [M + H]^+^ calcd for [C_19_H_20_BrNO_3_+H]^+^: 390.0700, found: 390.0696.

#### 1-(*tert*-Butylamino)-2,4-dimethyl-1-oxopentan-2-yl 4-Methylbenzoate (**6p**)

*tert*-Butyl isocyanide (141 μL, 1.3 mmol, 1.3 equiv) and *p*-toluic acid (131 mg, 1.0 mmol, 1 equiv) were added to a mixture of methyl isobutyl ketone (2.5 mL, 20.0 mmol, 20 equiv) and HFIP (625 μL), and the reaction was allowed to stir for 24 h. Then, the reaction was diluted with EtOAc and washed two times with 10 mL of NaHCO_3_ and one time with 10 mL of brine. The organic layers were dried over anhydrous Na_2_SO_4_. After the removal of the solvent, product **6p** was purified through chromatography and isolated as a colorless oil. Yield: 105 mg, 0.33 mmol, 33%. Column chromatography: silica, 10% EtOAc/heptane. ^1^H NMR (300 MHz, Chloroform-*d*) δ 7.87–7.81 (m, 2H), 7.29 (s, 2H), 6.23 (s, 1H), 2.43 (s, 3H), 2.22 (dd, *J* = 14.5, 5.9 Hz, 1H), 2.05 (dd, *J* = 14.5, 7.1 Hz, 1H), 1.76 (s, 3H), 1.39 (s, 9H), 0.92 (dd, *J* = 9.9, 6.7 Hz, 6H). ^13^C{^1^H} NMR (75 MHz, CDCl3) δ 172.0, 164.7, 143.9, 129.4, 129.3, 128.1, 85.7, 51.0, 44.6, 28.6, 24.6, 23.8, 23.3, 21.7. HRMS (ESI-TOF) *m*/*z*: [M + H]^+^ calcd for [C_19_H_29_NO_3_+H]^+^: 320.2220, found: 320.2222.

## Data Availability

The data underlying this study are available in the published article and its Supporting Information.

## Supporting Information Available

Experimental details, characterization data, and copies of ^1^H and ^13^C NMR spectra (PDF)
