# Supplementary material for: Strong Hydrogen Bond Donating Solvents Accelerate the Passerini Three-Component Reaction
Source: J Org Chem. 2025 Apr 3;90(14):5000–7. doi: 10.1021/acs.joc.5c00236 (PMC11998065; doi:10.1021/acs.joc.5c00236)
Supplement: Supplementary file 1 — jo5c00236_si_001.pdf [file jo5c00236_si_001.pdf]

# Supporting information

# Strong Hydrogen Bond Donating Solvents Accelerate the Passerini Three-Component Reaction

Claudio Ferdeghini[a‡], Minghui Wu[a‡], Prabhat Ranjan[a], M. A. Würdemann[a], Jan Pyschik[b], Alexander Mitsos[b], Eelco Ruijter,[c] Romano V.A. Orru[a], Thomas Hansen,[c*] Jordy M. Saya[a*]

[a.] Biobased Organic Chemistry, Aachen-Maastricht Institute for Biobased Materials (AMIBM), Maastricht University, Urmonderbaan 22, 6167RD Geleen, the Netherlands. [b.] Process Systems Engineering, RWTH Aachen University, Schinkelstrasse 8, Aachen 52062, Germany. [c.] Department of Chemistry & Pharmaceutical Sciences and Amsterdam Institute for Molecular & Life Science (AIMMS), Vrije Universiteit Amsterdam, De Boelelaan 1108, 1081 HZ Amsterdam, The Netherlands.

*E-mail: t.hansen@vu.nl
*E-mail: j.saya@maastrichtuniversity.nl
‡ Claudio Ferdeghini and Minghui Wu contributed equally to this manuscript.

## Table of Content

**1. General information** ........ S2

**2. Reaction Optimization and Kinetic Study** ........ S4

2.1 Calibration ........ S4

2.2 Kinetic measurements to investigate optimal HFIP concentration in $CH_2Cl_2$ ........ S5

2.3 Kinetic measurements to investigate alcohol cosolvents in $CH_2Cl_2$ ........ S6

2.4 Kinetic studies of HFIP (20 v/v%) in different solvents ........ S7

2.4.1 DCM ........ S8

2.4.2 $CHCl_3$ ........ S9

2.4.3 MeCN ........ S10

2.4.4 EtOAc ........ S11

2.4.5 TBME ........ S12

2.4.6 THF ........ S13

2.4.7 MeOH ........ S14

2.4.8 DMF ........ S15

2.4.9 HFIP (20 v/v%) in DCM vs aqueous LiCl (2.5M) and aqueous surfactant solution ........ S16

2.5 Confirmation of the Third-Order Kinetics of the Passerini Reaction ........ S17

2.5.1 Experimental set-up ........ S17

2.5.2 Kinetic studies parameter estimation ........ S18

2.5.3 Data analysis ........ S18

2.5.4 Kinetic studies on concentration dependence ........ S21

**3. Additional Details General Procedure** ........ S22

3.1 General procedure for Passerini reaction ........ S22

3.2 Reaction optimization for aromatic aldehydes ........ S23

**4. Passerini reaction of sterically hindered ketone 2p** ........ S24

**5. Computational Details** ........ S25

**6. Copies of NMR spectra** ........ S62

**7. References** ........ S78

# 1. General information

Commercially available reagents were purchased from Sigma-Aldrich, Fischer Scientific, Strem Chemicals, TCI Chemicals, Activate Scientific, or Fluorochem and were used as purchased unless mentioned otherwise. Solvents were purchased from VWR Chemicals or Sigma-Aldrich and used without purification, unless stated otherwise. Reagent grade solvent was used for the optimization. Thin layer chromatography (TLC) was performed using plates from Merck ($SiO_2$, Kieselgel 60 F254 neutral, on aluminium with fluorescence indicator) and compounds were visualized by UV detection (254 nm), $KMnO_4$, and/or hanessian's, stain. Liquid Chromatography – Mass Spectrometry (LC-MS) analysis was performed on a Shimadzu Nexera 2 UHPLC system equipped with a Shimadzu LC-30AD pump, an SPD-M30A photodiode array detector and LCMS-2020 single quadrupole detector. The system was run on MilliQ water and LC-MS grade acetonitrile both modified with 0.1% formic acid. A Waters XSelect CSH C18 column (3.0 mm x 75 mm with a particle size of 3.5 µm) was used operating at 30 °C. The method was set up with a gradient of 5% acetonitrile in water for 2 min, an increase to 95% acetonitrile over 12 min, 1 min at 95% followed by flushing back to 5% acetonitrile. Flash column chromatography was performed by employing silica (200-300 mesh) as support and *n*-heptane/ethyl acetate. NMR spectra were recorded on a Brüker Avance 300 using the residual $CDCl_3$ as internal reference ($^1H$: δ 7.26 ppm, $^{13}C$: δ 77.16 ppm). Chemical shifts (δ) are given in ppm and coupling constants (J) are quoted in hertz (Hz). Resonances are described as s (singlet), d (doublet), t (triplet), q (quartet), br (broad singlet), and m (multiplet) or combinations thereof. Ultra-high resolution mass-spectrometer Bruker solariX XR FT-ICR-MS was used for accurate mass measurements. Samples were ionized by electrospray ionization (ESI) in positive ion mode. NMR data were processed with Mestrenova version 12.

# 2. Reaction Optimization and Kinetic Study

## 2.1 Calibration

The calibration is performed by using 1-(*tert*-butylamino)-1-oxo-3-phenylpropan-2-yl acetate (**6a**) as target product and biphenyl (**IS**) as internal standard. The calibration curve is made according to the response of integral area to concentration of 6a and IS in PDA of LCMS. Three repetitive calibration measurement has been performed, and fitting curve is made based on the mean value of three measurements.

**Table S1.** PDA integration of **6a** and **IS**.

| Concentration | Calibration 1 | | Calibration 2 | | Calibration 3 | |
|---|---|---|---|---|---|---|
| Mol/L | Integra **6a** | Integra **IS** | Integra **6a** | Integra **IS** | Integra **6a** | Integra **IS** |
| 0 | 0 | 0 | 0 | 0 | 0 | 0 |
| 0.00005 | 209611 | 708347 | 184365 | 718863 | 193728 | 737477 |
| 0.00025 | 901597 | 3616531 | 922493 | 3633584 | 945325 | 3847968 |
| 0.0005 | 1918460 | 7198413 | 1885265 | 7291748 | 2053741 | 7690343 |
| 0.00125 | 4723635 | 18214600 | 4679211 | 18288320 | 5410041 | 19426519 |
| 0.0025 | 9275603 | 35907939 | 9297855 | 36545803 | 10262919 | 38342839 |

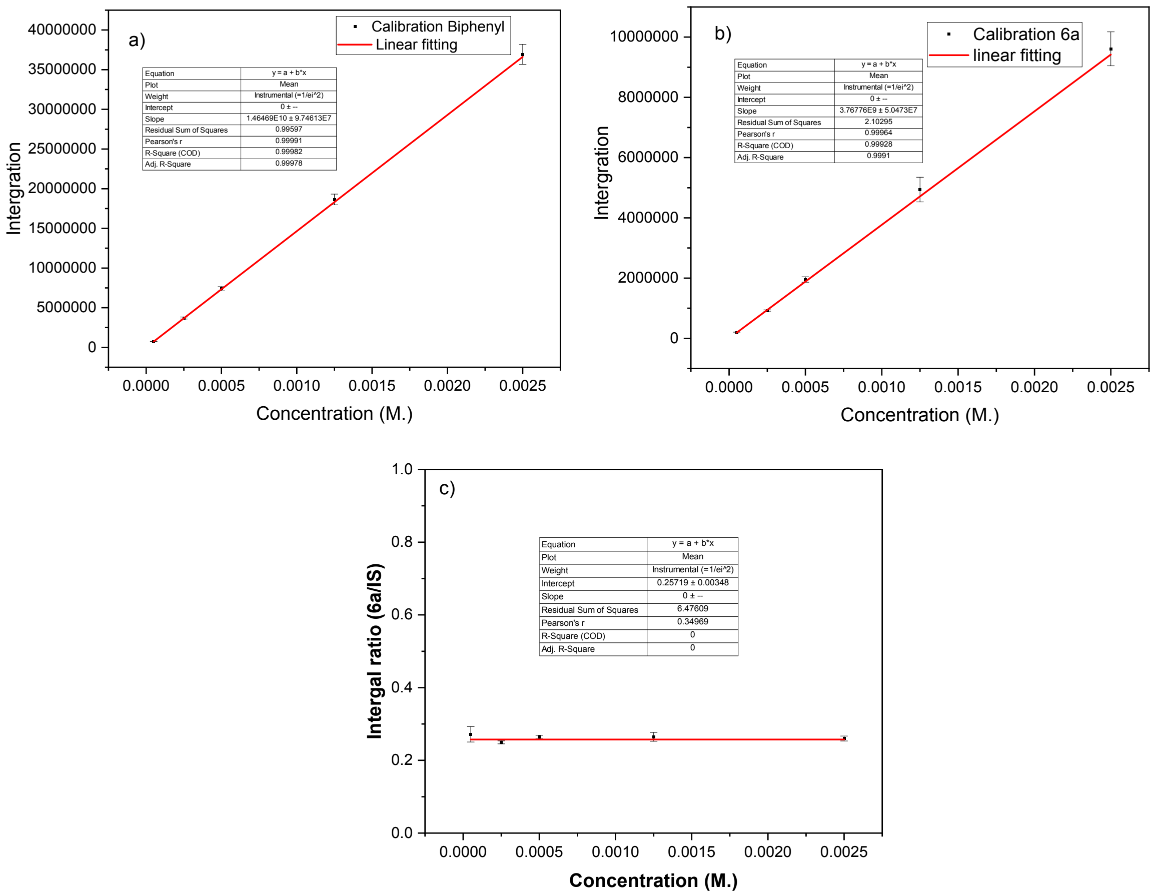


**Figure S1.** a) Calibration curve of IS; b) Calibration curve of 6a; c) integral response of 6a/IS.

## 2.2 Kinetic measurements to investigate optimal HFIP concentration in $CH_2Cl_2$

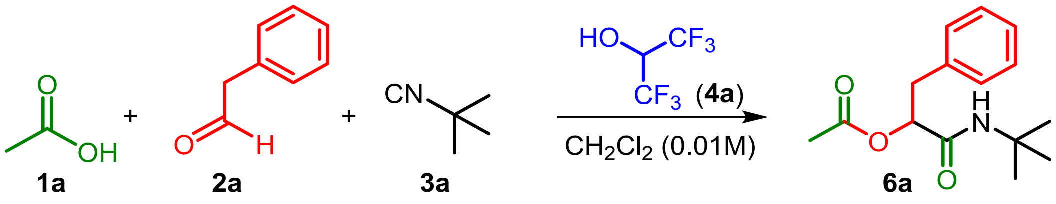


These investigations are conducted by comparing the yield of reaction with different ratios of HFIP in $CH_2Cl_2$. Firstly, to 9500 µL of mixture of (0 to 100 v/v%) HFIP in $CH_2Cl_2$, acetic acid (5.7 µL, 0.10 mmol, 1 equiv) and 2-phenylacetaldehyde (11 µL, 0.10 mmol, 1 equiv) were added and reaction mixture was keeping stirring. After that, *tert*-butyl isocyanide (12 µL, 0.10 mmol, 1 equiv) was added in the reaction, in the same time the timer was started to record. The samples are taken from reaction mixture and measured by LCMS after 1, 2, 5, 10, 30 and 60 minutes. For each sample, 100 µL reaction mixture and 50 µL biphenyl solution (0.02 M) were added in quenching solution (1% $Et_3N$ in MeCN).

After LCMS measurements, the yield was calculated based on the integral ratio of **6a/IS** and shown in Figure S2.

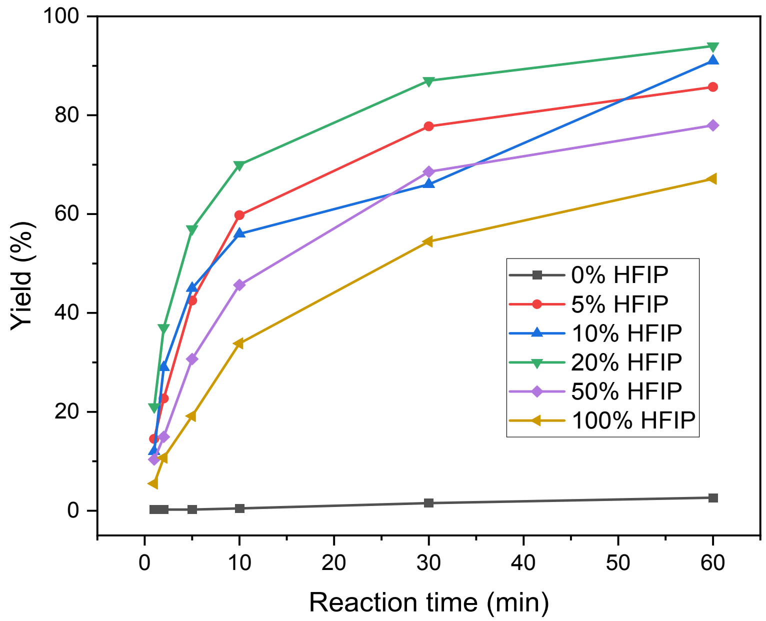


**Figure S2.** The reaction yield of various ratio HFIP in $CH_2Cl_2$ from 0 to 60 minutes.

## 2.3 Kinetic measurements to investigate alcohol cosolvents in $CH_2Cl_2$

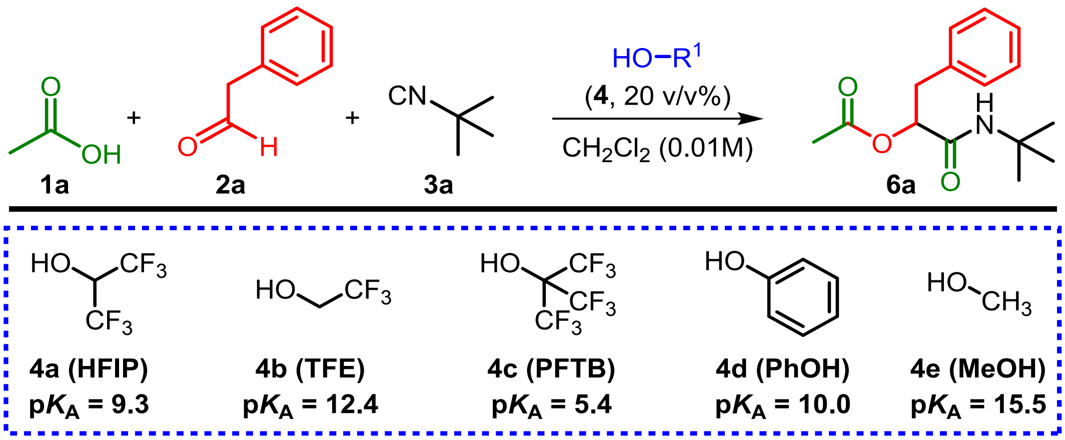


These investigations are conducted by comparing the yield of reaction with different alcohol cosolvents (20 v/v%) in $CH_2Cl_2$. Firstly, to 9500 µL of mixture of alcohol **4** (20 v/v%) in $CH_2Cl_2$, acetic acid (5.7 µL, 0.10 mmol, 1 equiv) and 2-phenylacetaldehyde (11 µL, 0.10 mmol, 1 equiv) were added and reaction mixture was keeping stirring. After that, *tert*-butyl isocyanide (12 µL, 0.10 mmol, 1 equiv) was added in the reaction, in the same time the timer was started to record. The samples are taken from reaction mixture and measured by LCMS after 1, 2, 5, 10, 30 and 60 minutes. For each sample, 100 µL reaction mixture and 50 µL standard solution (0.02 M) were added in quenching solution (1% $Et_3N$ in MeCN).

After LCMS measurements, the yield was calculated based on the integral ratio of **6a/IS** and shown in Figure S3.

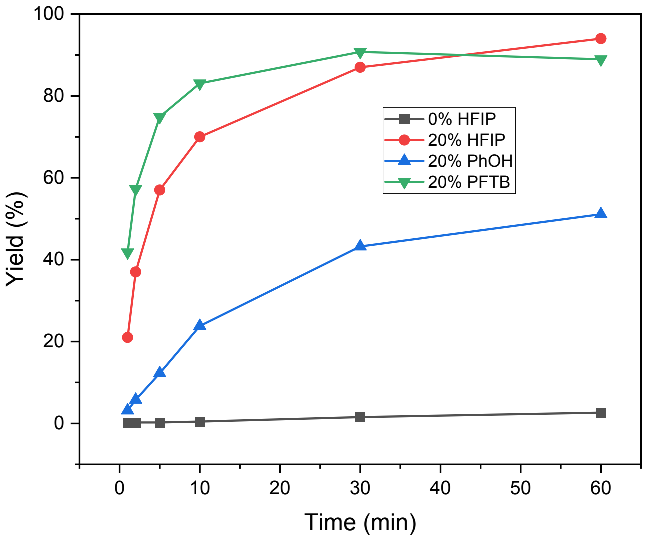


**Figure S3.** The reaction yield of different alcohol cosolvents (20 v/v %) in $CH_2Cl_2$ from 0 to 60 minutes.

## 2.4 Kinetic studies of HFIP (20 v/v%) in different solvents

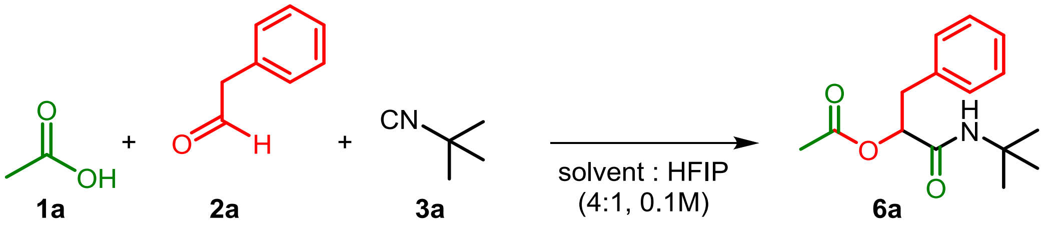


The kinetic study is conducted by comparing the LC-MS yield of the reaction when using different solvent. Firstly, to 971.2 µL of solvent were put in a vial and biphenyl (3.1 mg, 0.02 mmol) was added as internal standard. Then, in order were added, 2-phenylacetaldehyde (11.3 µL, 0.1 mmol, 1.0 equiv), *tert*-butyl isocyanide (11.7 µL, 0.1 mmol, 1.0 equiv) and acetic acid (5.7 µL, 0.10 mmol, 1.0 eq) and the timer was started to record. The samples are taken from reaction mixture and measured by LCMS after 2, 5, 10, 15, 30, 60, 120 and 180 minutes. For each sample, 100 µL of reaction mixture were taken and 5 µL of $Et_3N$ were added to quench the reaction. 895 µL of MeCN were added and the solution was filtered through a 0.2 µm PTFE filter.

### 2.4.1 DCM

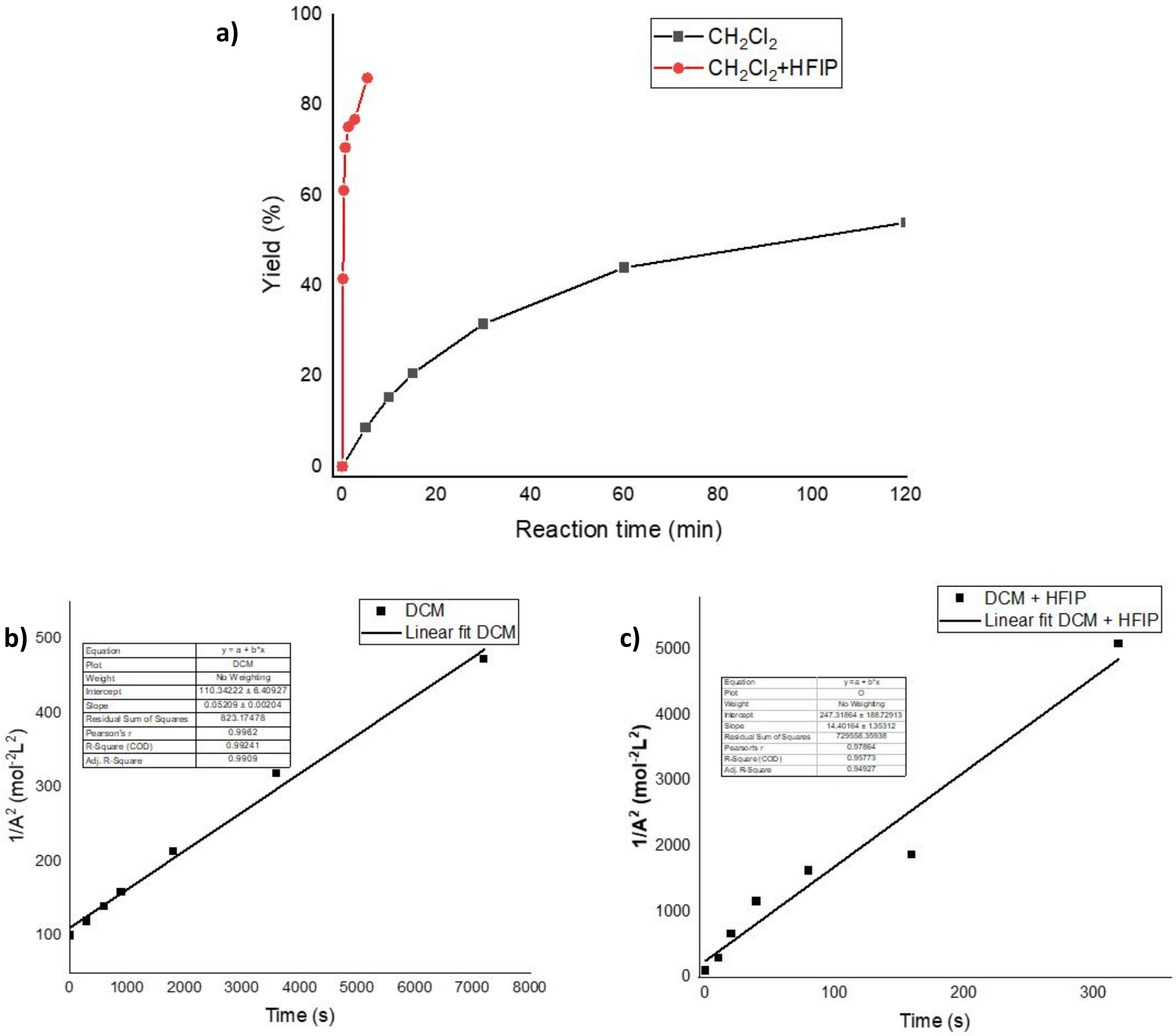


**Figure S4**. a) Reaction progress in DCM versus in HFIP (20 v/v%) in DCM. b) Third order kinetic plot for a Passerini reaction in DCM. c) Third order kinetic plot for a Passerini reaction in HFIP (20 v/v%) in DCM.

### 2.4.2 $CHCl_3$

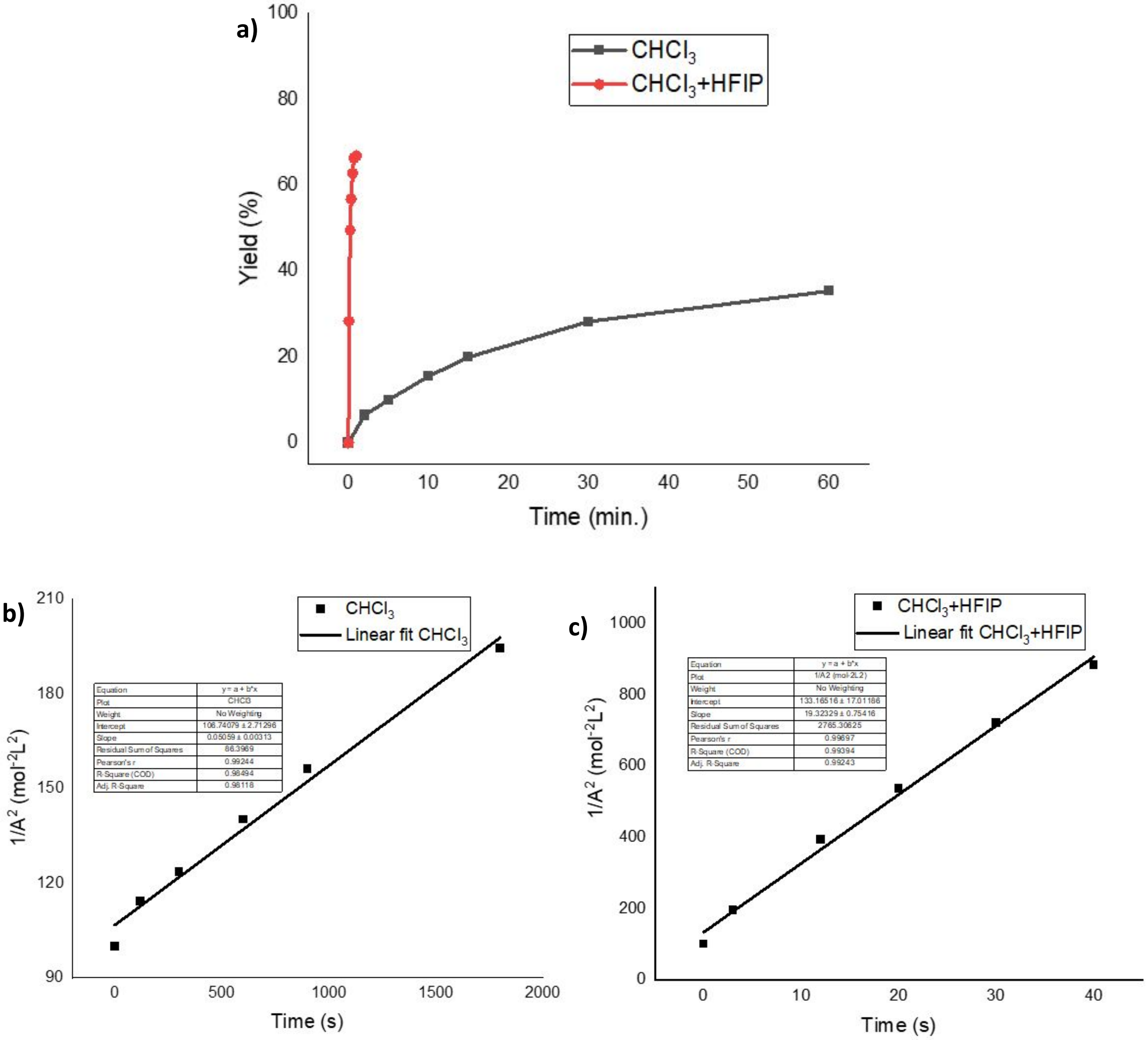


**Figure S5**. a) Reaction progress in $CHCl_3$ versus in HFIP (20 v/v%) in $CHCl_3$. b) Third order kinetic plot for a Passerini reaction in $CHCl_3$. c) Third order kinetic plot for a Passerini reaction in HFIP (20 v/v%) in $CHCl_3$.

### 2.4.3 MeCN

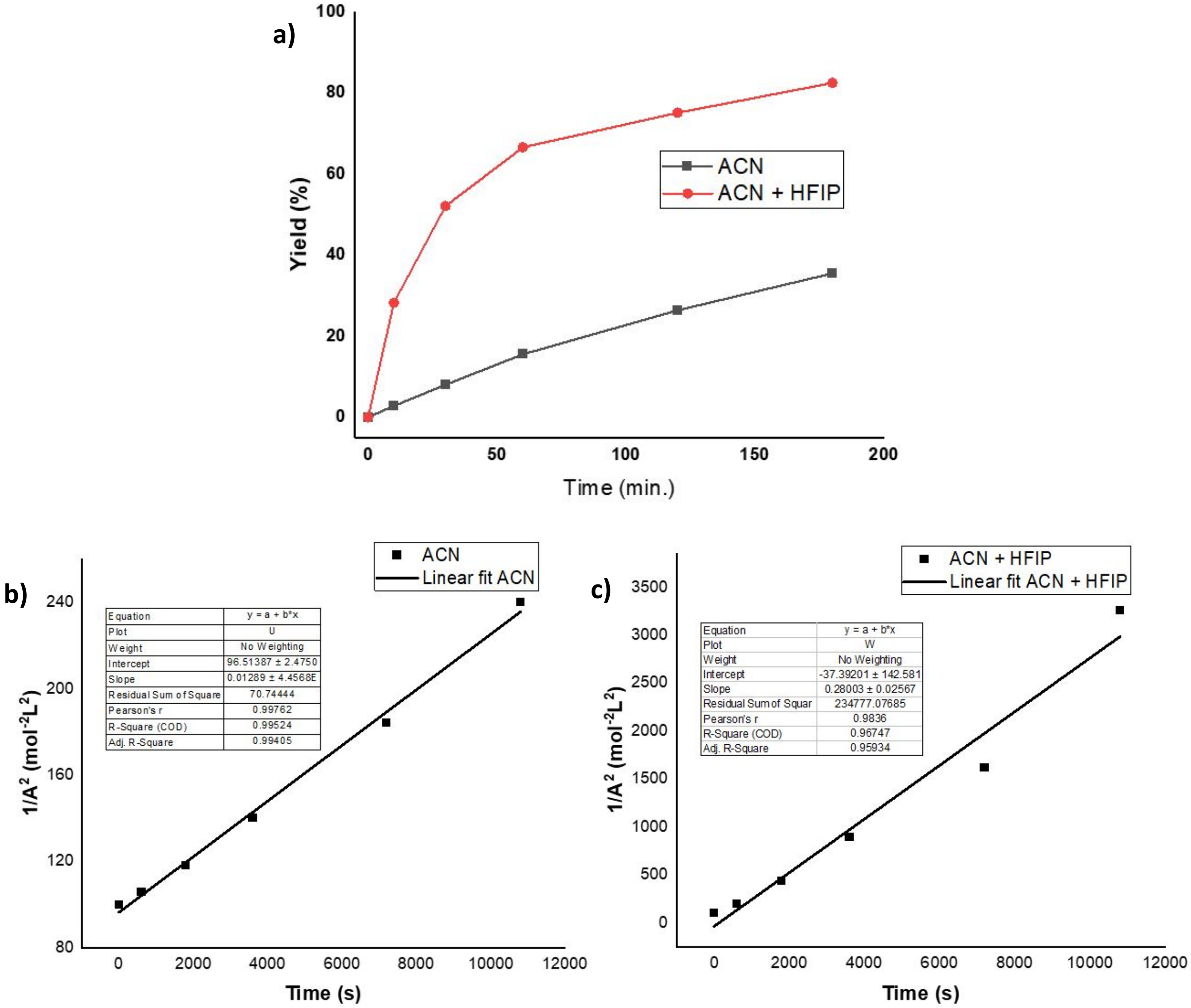


**Figure S6**. a) Reaction progress in MeCN versus in HFIP (20 v/v%) in MeCN. b) Third order kinetic plot for a Passerini reaction in MeCN. c) Third order kinetic plot for a Passerini reaction in HFIP (20 v/v%) in MeCN.

## 2.4.4 EtOAc

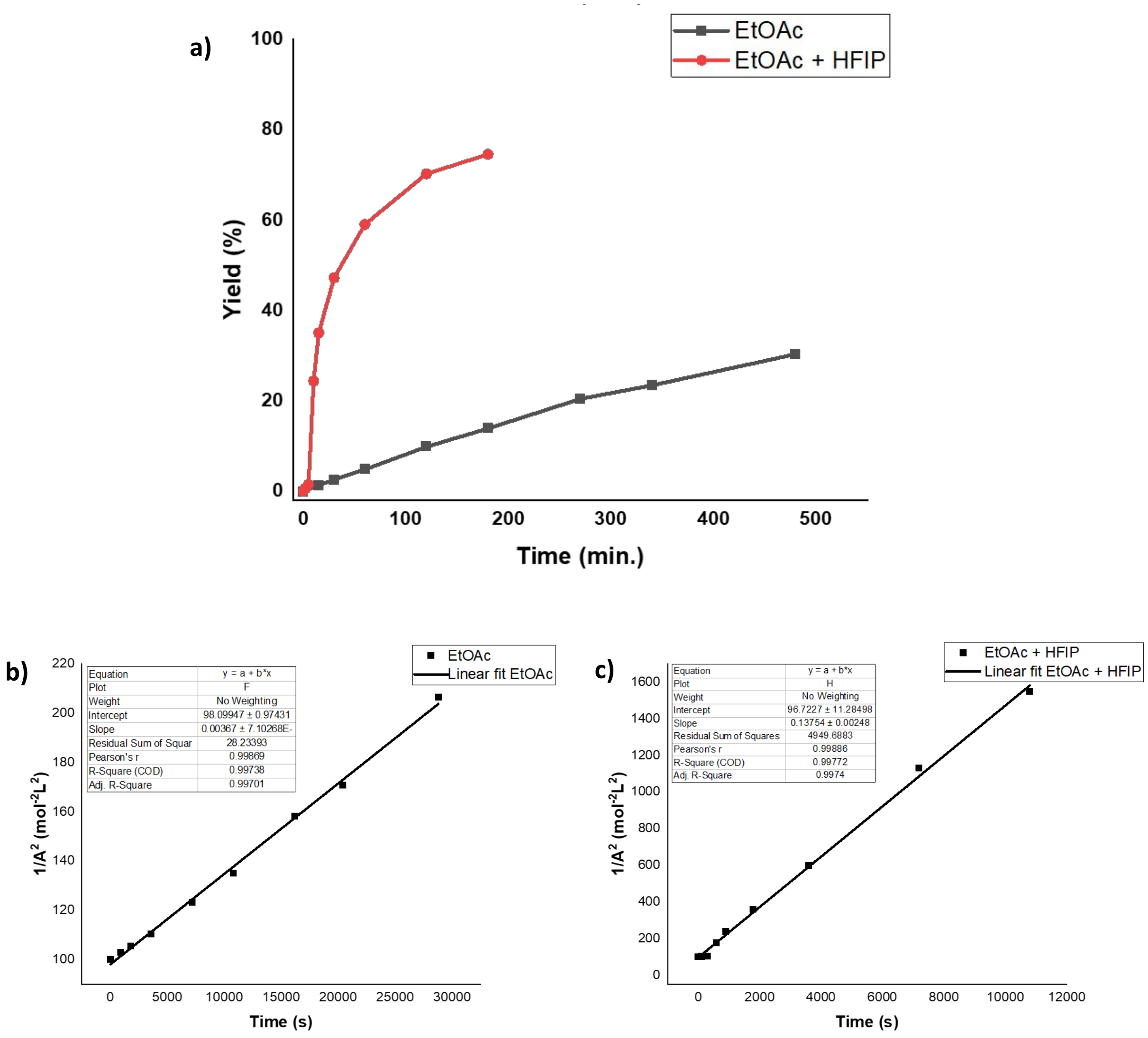


**Figure S7**. a) Reaction progress in EtOAc versus in HFIP (20 v/v%) in EtOAc. b) Third order kinetic plot for a Passerini reaction in EtOAc. c) Third order kinetic plot for a Passerini reaction in HFIP (20 v/v%) in EtOAc.

## 2.4.5 TBME

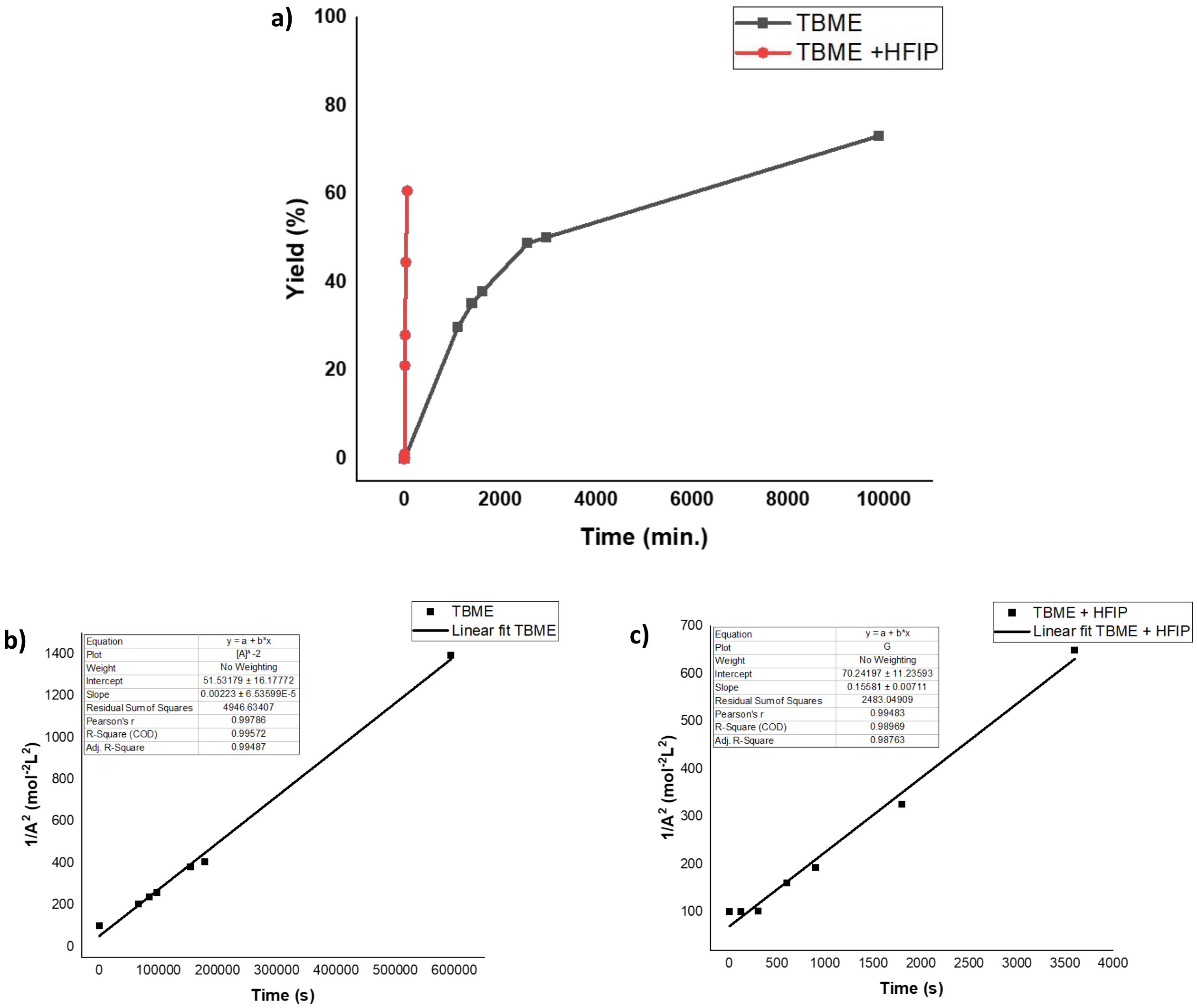


**Figure S8**. a) Reaction progress in TBME versus in HFIP (20 v/v%) in TBME. b) Third order kinetic plot for a Passerini reaction in TBME. c) Third order kinetic plot for a Passerini reaction in HFIP (20 v/v%) in TBME.

### 2.4.6 THF

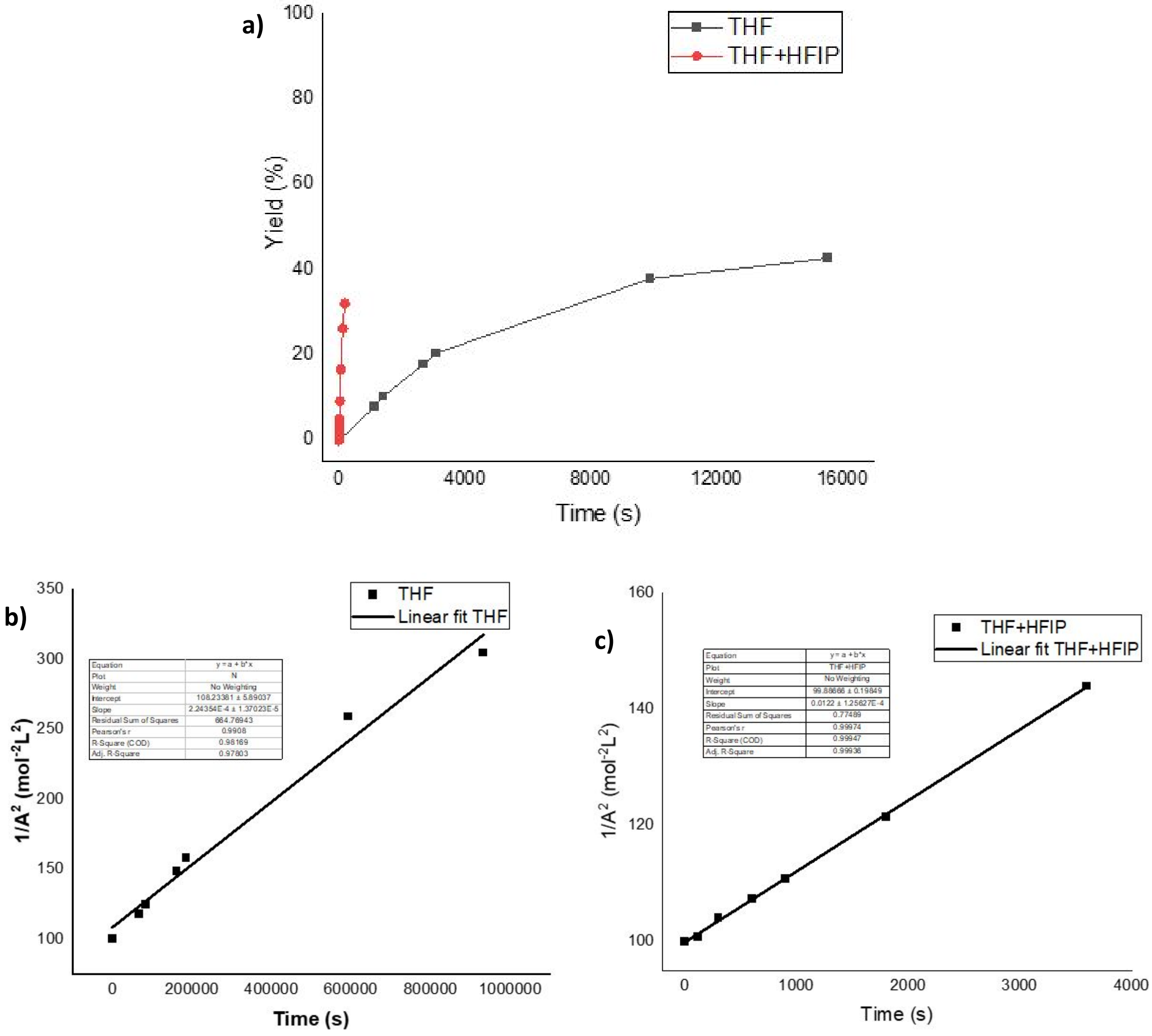


**Figure S9**. a) Reaction progress in THF versus in HFIP (20 v/v%) in THF. b) Third order kinetic plot for a Passerini reaction in THF. c) Third order kinetic plot for a Passerini reaction in HFIP (20 v/v%) in THF.

## 2.4.7 MeOH

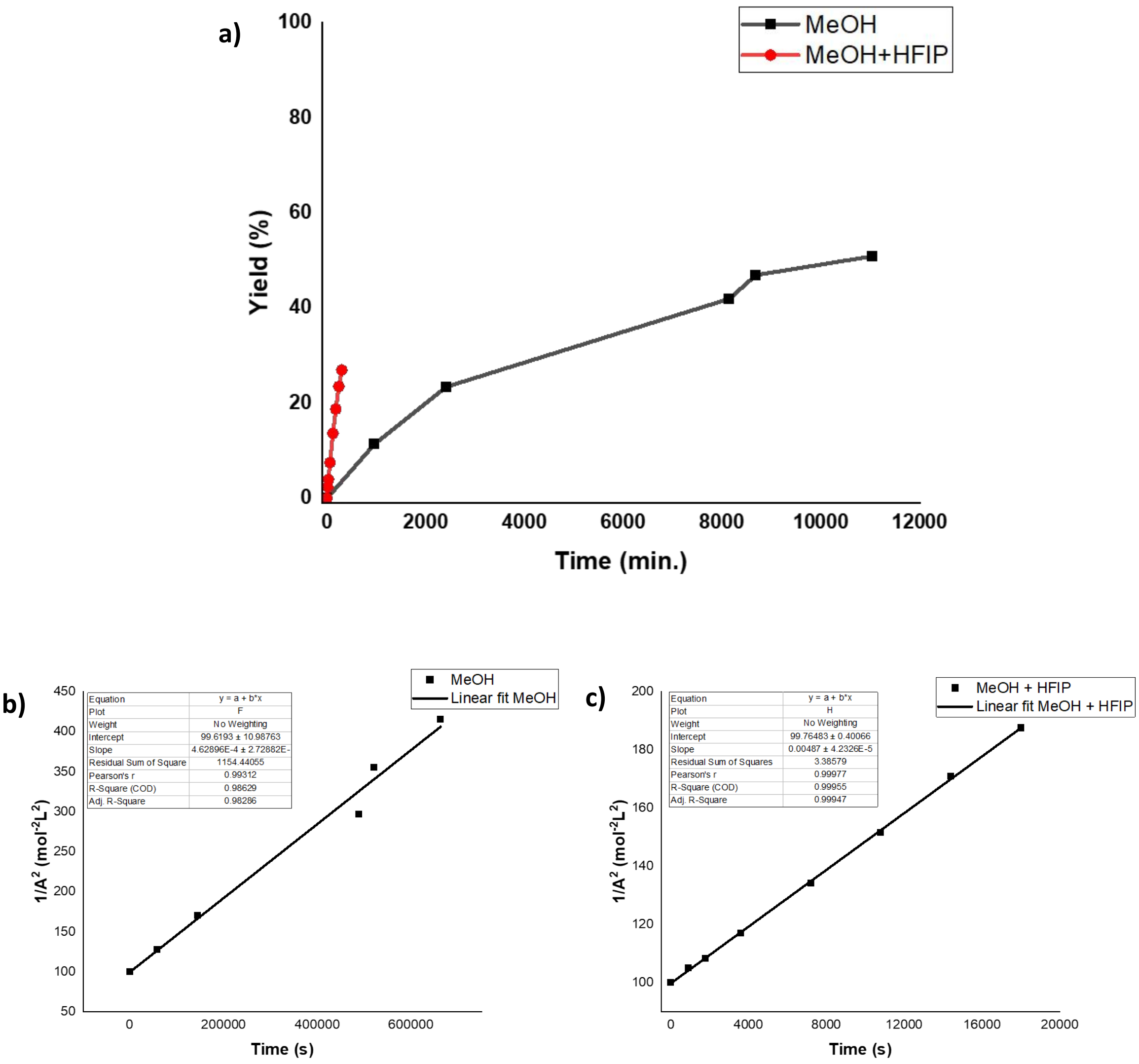


**Figure S10**. a) Reaction progress in MeOH versus in HFIP (20 v/v%) in MeOH. b) Third order kinetic plot for a Passerini reaction in MeOH. c) Third order kinetic plot for a Passerini reaction in HFIP (20 v/v%) in MeOH.

## 2.4.8 DMF

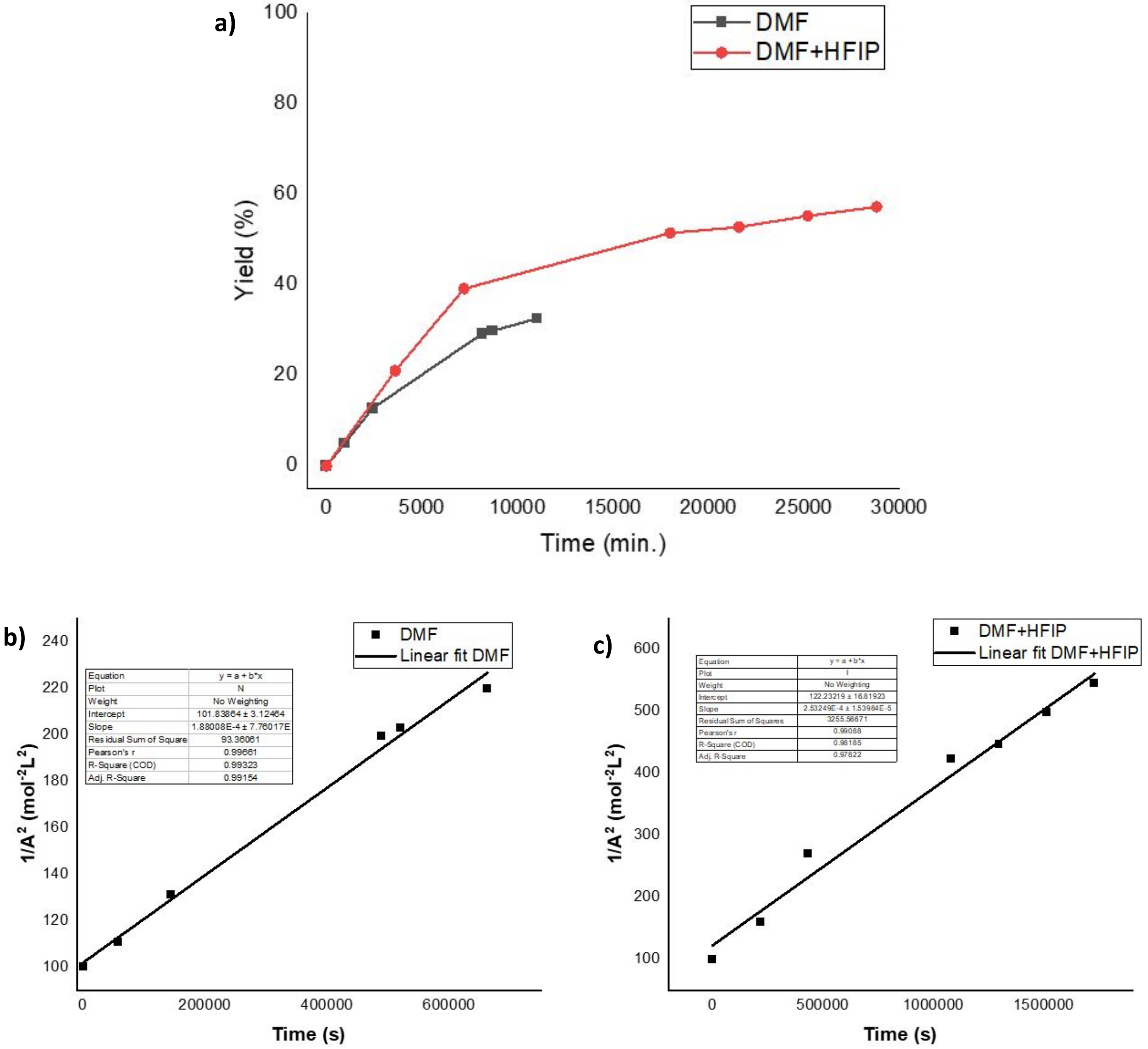


**Figure S11**. a) Reaction progress in DMF versus in HFIP (20 v/v%) in DMF. b) Third order kinetic plot for a Passerini reaction in DMF. c) Third order kinetic plot for a Passerini reaction in HFIP (20 v/v%) in DMF.

### 2.4.9 HFIP (20 v/v%) in DCM vs aqueous LiCl (2.5M) and aqueous surfactant solution

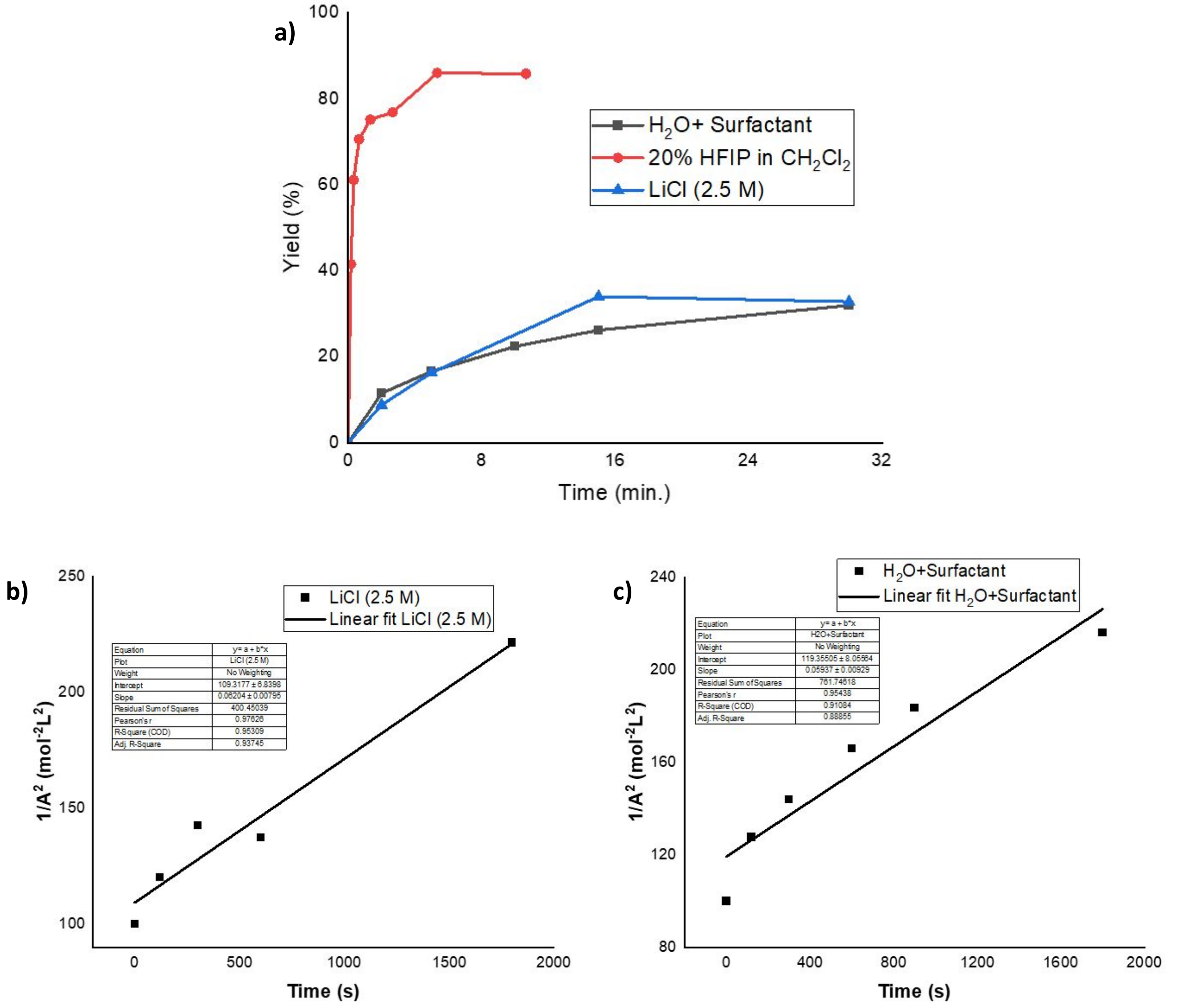


**Figure S12**. a) Reaction progress in aqueous LiCl (2.5M) and aqueous surfactant solution versus in HFIP (20 v/v%) in DCM. b) Third order kinetic plot for a Passerini reaction in aqueous LiCl (2.5M). c) Third order kinetic plot for a Passerini reaction in aqueous surfactant solution.

## 2.5 Confirmation of the Third-Order Kinetics of the Passerini Reaction

### 2.5.1 Experimental set-up

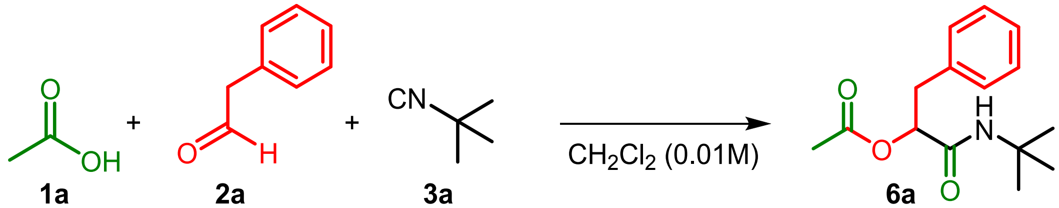


To demonstrate that the Passerini reaction follows a third-order rate law, the reaction was conducted at a concentration of 0.1 M for each reagent: acetic acid (**1a**), 2-phenylacetaldehyde (**2a**), *tert*-butyl isocyanide (**3a**) in $CH_2Cl_2$ (Table S2). To determine the reaction order with respect to each reagent, the concentration of one reagent was systematically doubled (0.2 M), while the other two reagents were kept at their initial concentration (0.1 M).

**Table S2.** Reagent concentrations to determine a third-order rate law in the Passerini reaction.

| | **1a** | **2a** | **3a** |
|---|---|---|---|
| Equimolar ratio | 0.1 | 0.1 | 0.1 |
| 2 x [**1a**] | 0.2 | 0.1 | 0.1 |
| 2 x [**2a**] | 0.1 | 0.2 | 0.1 |
| 2 x [**3a**] | 0.1 | 0.1 | 0.2 |

Samples were taken from the reaction mixture and measured by LCMS after 5, 10, 15, 30 and 60 minutes. For each sample, 100 µL reaction mixture and 50 µL biphenyl solution (0.02 M) were added in quenching solution (1% $Et_3N$ in MeCN). After LCMS measurements, the yield was calculated based on the integral ratio of **6a**/**IS** and shown in Figure S13.

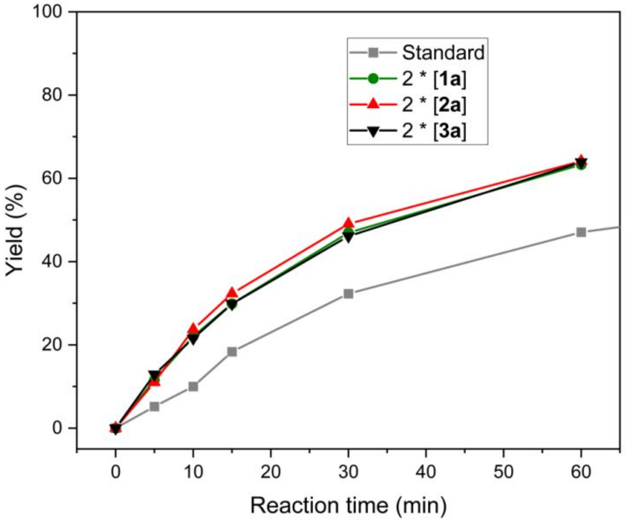


**Figure S13.** Reaction progress in DCM to determine the order of the reaction.

### 2.5.2 Kinetic studies parameter estimation

The three reagents are abbreviated with *A,B,C*, in the following. We assume that volume is constant and therefore we can use concentrations $C_A(t)$, $C_B(t)$, $C_C(t)[\frac{\text{mol}}{\text{L}}]$ in the mass-balances. Let $C_{A0}$, $C_{B0}$, $C_{C0}$ be the initial concentrations. Let $r(t)$ be the reaction rate $[\frac{\text{mol}}{\text{L}*\text{s}}]$:

Species balance gives us for each time instant

$$\left.\frac{dC_A}{dt}\right|_t = \left.\frac{dC_B}{dt}\right|_t = \left.\frac{dC_C}{dt}\right|_t = -r(t)$$

We can eliminate two of the concentrations as

$$C_B(t) = C_{B0} - C_{A0} + C_A(t),\ C_C(t) = C_{C0} - C_{A0} + C_A(t).$$

The reaction rate $r$ depends on the current concentrations. Assuming first order in each species, we get

$$r(t) = k\ C_A(t)\ C_B(t) C_C(t)$$

Where $k$ is the reaction constant $[\frac{\text{L}^6}{\text{mol}^2\text{s}}]$ for the given temperature. Combining the equations, we obtain

$$\left.\frac{dC_A}{dt}\right|_t = -r(t) = -\ k\ C_A(t) C_B(t) C_C(t) = -kC_A(t)(C_{B0} - C_{A0} + C_A(t))(C_{C0} - C_{A0} + C_A(t))$$

If $C_{B0} = C_{A0} = C_{C0}$, this equation simplifies to

$$\left.\frac{dC_A}{dt}\right|_t = -kC_A(t)^3$$

which can be integrated analytically to

$$\frac{1}{C_A(t)^2} = 2kt + \frac{1}{C_{A0}{}^2}$$

Thus, if the inverse of the squared concentrations ($\frac{1}{C_A{}^2}$) is plotted over time, the slope is equal to $2k$. In other words, by analytically solving the differential equations, the parameter estimation is reduced to a simple linear regression that can be solved by plotting.

### 2.5.3 Data analysis

To investigate the reaction order four different mixtures of the reactants were tested.

| | $C_{A0}$ | $C_{B0}$ | $C_{C0}$ |
|---|---|---|---|
| Equimolar ratio | 0.1 | 0.1 | 0.1 |
| 2 x [**1a**] | 0.2 | 0.1 | 0.1 |
| 2 x [**2a**] | 0.1 | 0.2 | 0.1 |
| 2 x [**3a**] | 0.1 | 0.1 | 0.2 |

However, the analytical/graphical solution of the differential equation assumed equimolarity (see SI 2.5). Hence, the parameter estimation in the following was done numerically in Python.

The solution is split into two parts. First, a model is built in Pyomo and the differential equation is solved.

$$\left.\frac{dC_A}{dt}\right|_t = -\,k\;C_A(t)^n\;C_B(t)^n\;C_C(t)^n = -\,k\;C_A(t)^n\;(C_{B0}-C_{A0}+C_A(t))^n\;(C_{C0}-C_{A0}+C_A(t))^n$$

Secondly, we define an objective function and let it be optimized to get a good fit. This is done by finding the parameter values for k and n, that minimize the sum (over both species and experiments) of all squared errors between the concentration calculated by the mathematical model $C_A$and measured in the lab $\hat{C}_A$.

$$\min_{k,n} \sum_{h=0}^{n_{exp}} \sum_{j=0}^{n_{meas}} \left(C_A(t_{j,h}) - \hat{C}_A(t_{j,h})\right)^2$$

In one case we let the optimizer choose the exponent n freely, in the other we fixed n=1.

## Results

### Variable exponent n

We plotted the reaction yield over time for the optimized values k=0.47 and n=0.82. The sum of all squared errors is: 0.000072

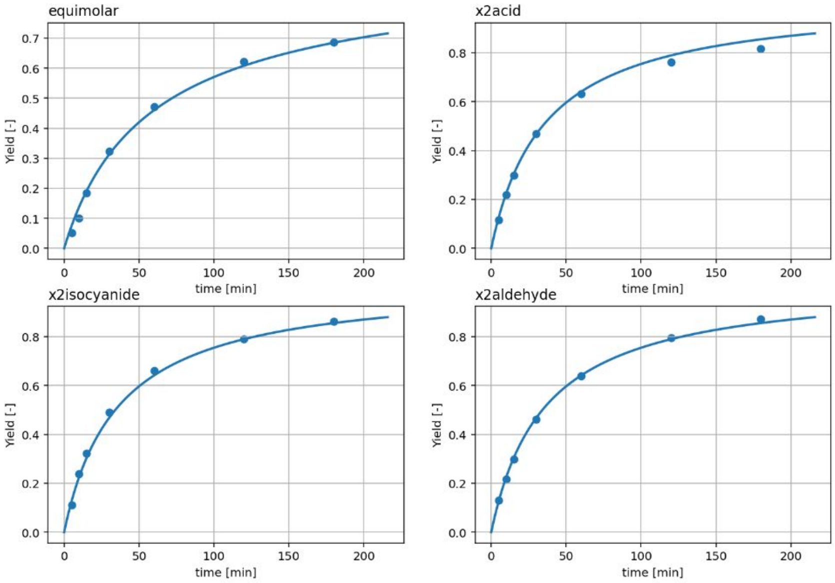


**Figure S14.** Reaction yield over time for the optimized values k=0.47 and n=0.82.

### Fixed exponent n=1

We now plot the reaction yield over time for fixed n=1 and optimized k=1.83. The sum of all squared errors is: 0.00026

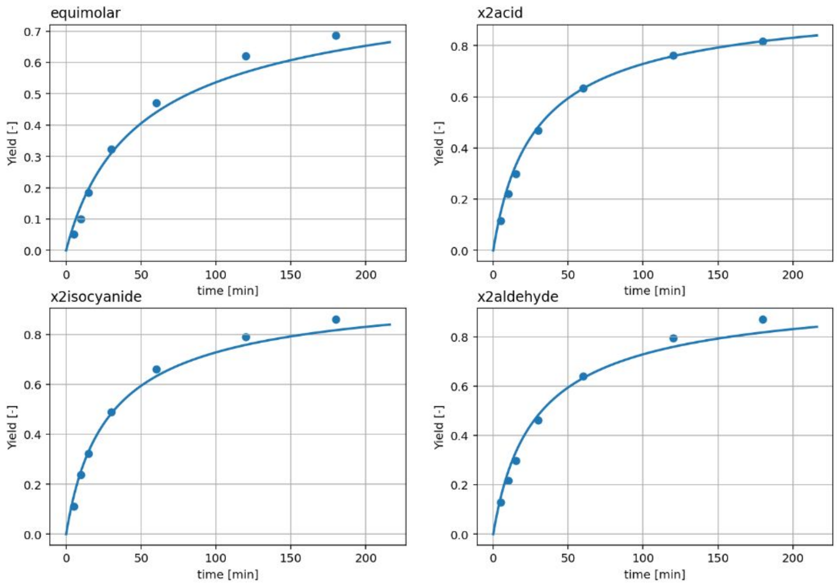


**Figure S15.** Reaction yield over time for fixed n=1 and optimized k=1.83.

## Comparison

The sum of all squared errors is slightly better if n is included in the optimization. However, both sums are very small in comparison to the experimental error and thus based on these experiments first-order reaction of each reagent can be considered valid.

Python packages used

| | |
|---|---|
| Pyomo | Modeling |
| Numpy | Maths |
| Pandas | Data |
| Casadi | Solving DAE |
| scipy | Optimization |
| Matplotlib | Plotting |

## Burés method to determine reaction order

The plots below show the product concentration over the *normalized* reactant concentration.[1] Case 1 has equimolar initial conditions. Case 2 has double the initial concentration of the reactant of interest.
In all other regards the experiments are identical. Conducting human visual analysis, it can be seen that the reaction orders of 0.82 and 1 appear to be a good fit.
A good judgement of the best fit is made difficult, in all plots, by the two curves not fully overlapping.

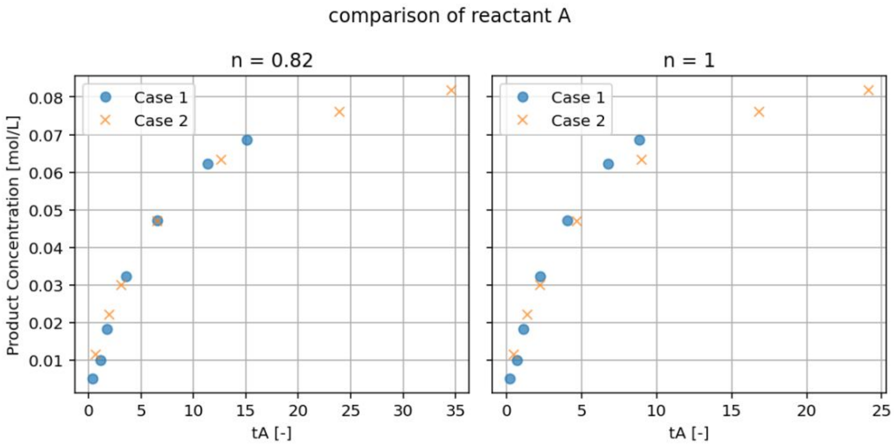


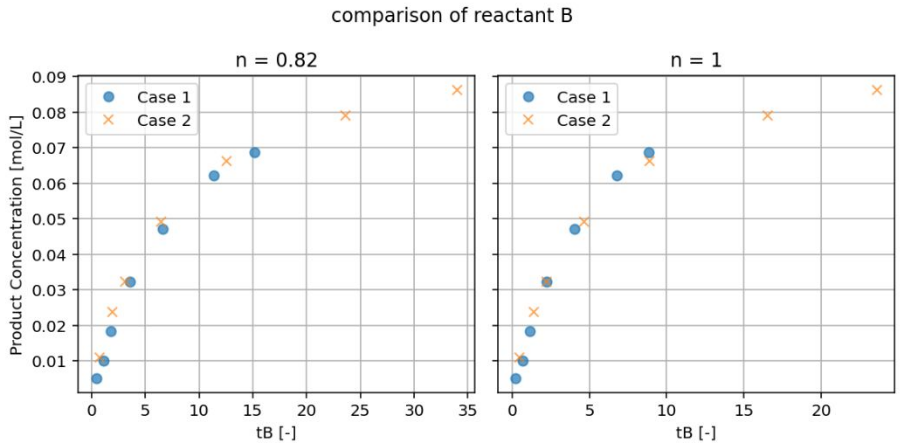


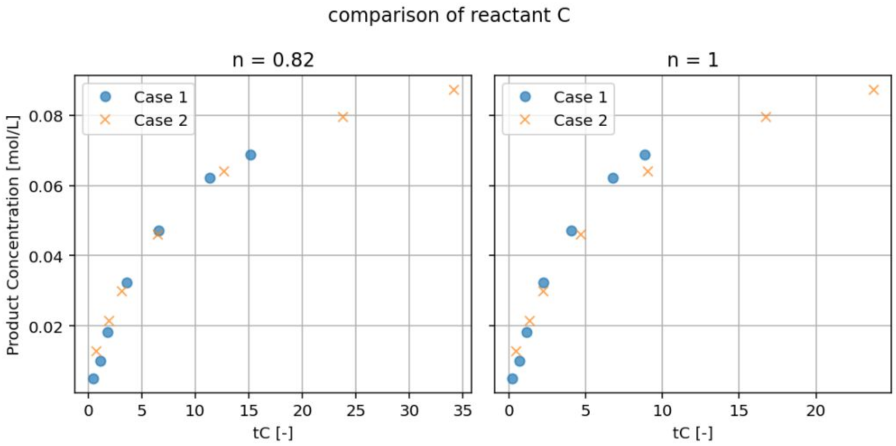


### 2.5.4 Kinetic studies on concentration dependence

Kinetic studies were conducted in DCM and DCM/HFIP to confirm the consistency of the rate constant under equimolar conditions at different reagent concentrations using the third-order rate law model.

**Table S3.** Rate constants based on third-order rate equation for different concentrations

| Concentration mol/L | Solvent | *k* ($mol^{-2} \cdot L^2 \cdot s^{-1}$) |
|---|---|---|
| 0.1 | DCM | 0.026 |
| 0.5 | DCM | 0.027 |
| 1 | DCM | 0.026 |
| 0.01 | DCM/HFIP (4:1) | 7.08 |
| 0.02 | DCM/HFIP (4:1) | 8.37 |
| 0.05 | DCM/HFIP (4:1) | 7.64 |

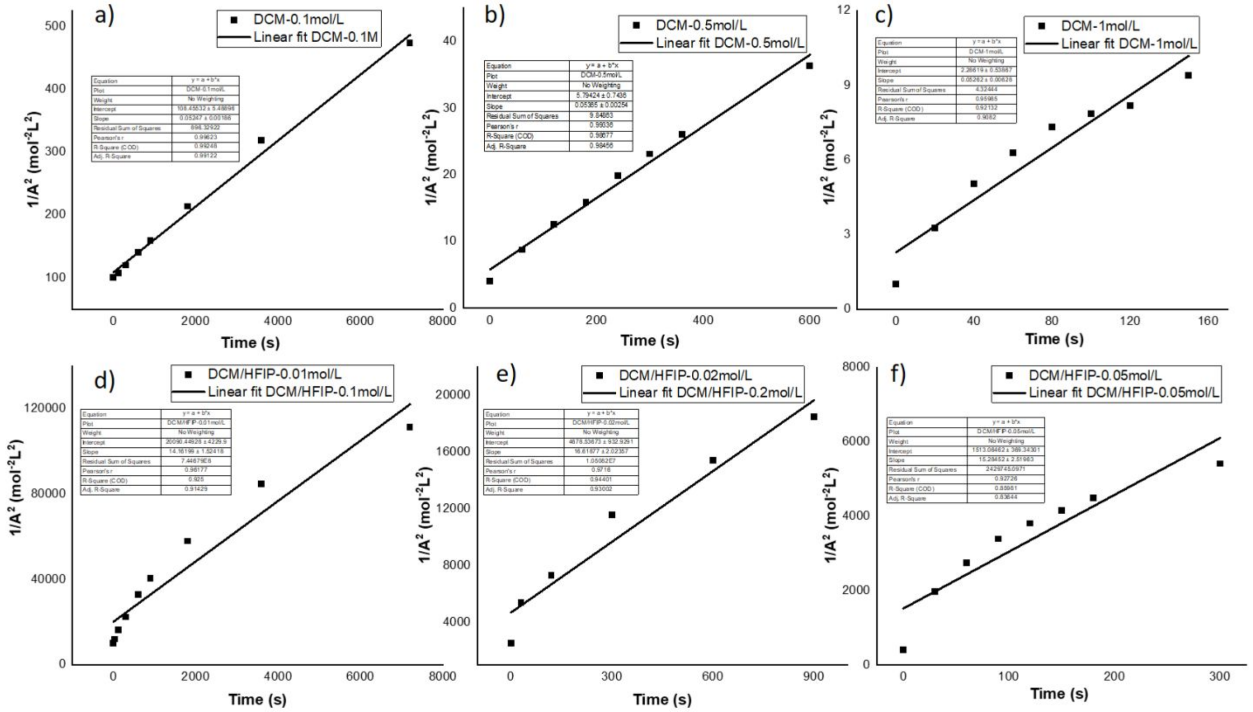


**Figure S16**. a) Third order kinetic plot for a Passerini reaction in DCM (0.1 M). b) Third order kinetic plot for a Passerini reaction in DCM (0.5 M). c) Third order kinetic plot for a Passerini reaction in DCM (1 M). d) Third order kinetic plot for a Passerini reaction in HFIP (20 v/v%) in DCM (0.01 M). e) Third order kinetic plot for a Passerini reaction in HFIP (20 v/v%) in DCM (0.02 M). f) Third order kinetic plot for a Passerini reaction in HFIP (20 v/v%) in DCM (0.05 M).

# 3. Additional Details General Procedure

## 3.1 General procedure for Passerini reaction

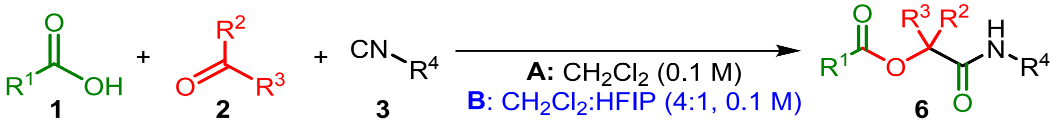


To a solution of isocyanide **3** (1.2 mmol, 1.2 equiv) and acid **1** (1.5 mmol, 1.5 equiv) in solvent (10 mL, 0.1 M), the carbonyl compound **2** (1.0, 1.0 equiv) was added and the reaction was allowed to stir for a specific time. Then the reaction was washed two times with 10 mL of $NaHCO_3$ and one time with 10 mL of brine.

The organic layers were dried over anhydrous $Na_2SO_4$. After the removal of the solvent, product **6** was purified through chromatography.

**Solvent**: DCM or DCM:HFIP = 80:20

**Reaction time:**

| Substrate | Solvent (0.1 M) | Time (h) |
|---|---|---|
| Aliphatic aldehyde | DCM | 8 |
| | DCM:HFIP (80:20) | 1 |
| Aromatic aldehyde | DCM | 24 |
| | DCM:HFIP (80:20) | 24 |
| 2,3-butanedione | DCM | 16 |
| | DCM:HFIP (80:20) | 2 |

## 3.2 Reaction optimization for aromatic aldehydes

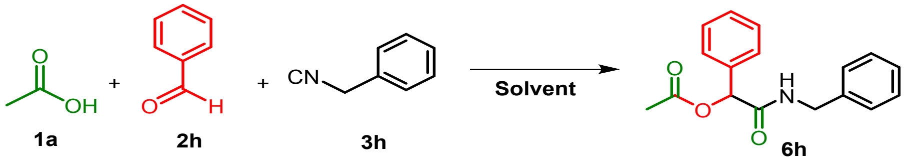


**Table S4** Optimization table on time and concentration for low yield molecules.

| Entry | Solvent | Concentration | Time | Yield |
|---|---|---|---|---|
| 1 | DCM | 0.1 M | 6 h | 22% |
| 2 | DCM | 0.5 M | 6 h | 28% |
| 3 | DCM | 1 M | 6 h | 50% |
| 4 | DCM | 1 M | 12 h | 68% |
| 5 | DCM | 1 M | 24 h | 70% (65%*) |
| 6 | DCM/HFIP (4:1) | 1 M | 1 h | 60% |
| 7 | DCM/HFIP (4:1) | 1 M | 3 h | 70% |
| 8 | DCM/HFIP (4:1) | 1 M | 6 h | 80% |
| 9 | DCM/HFIP (4:1) | 1 M | 12 h | 86% (84%*) |
| 10 | DCM/HFIP (4:1) | 1 M | 24 h | 88% |

*isolated yield

## 4. Passerini reaction of sterically hindered ketone 2p

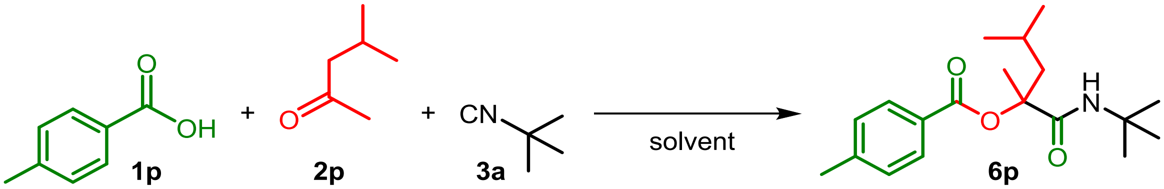


**Table S5.** Optimization table hindered ketone **2p**.

| Entry | Reagent Ratio (1p:2p:3a) | Solvent | Solvent Ratio (HFIP:solvent) | Concentration | Time | Yield |
|---|---|---|---|---|---|---|
| 1 | 1:1.2:1.3 | DCM | No HFIP | 0.1 M | 24 h | n.r. |
| 2 | 1:1.2:1.3 | DCM | 1:4 | 0.1 M | 24 h | n.r. |
| 3 | 1:40:1.3 | **2p** | 1:4 | 0.16 M | 24 h | 26% |
| 4 | 1:40:1.3 | **2p** | 1:4 | 0.16 M | 48 h | 33% |
| 5 | 1:40:1.3 | **2p** | 1:4 | 0.16 M | 72 h | 40% |
| 6 | 1:20:1.3 | **2p** | 1:4 | 0.31 M | 24 h | 39%(33%*) |
| 7 | 1:20:1.3 | **2p** | 1:4 | 0.31 M | 72 h | 50% |
| 8 | 1:10:1.3 | **2p** | 1:4 | 0.59 M | 24 h | 25% |
| 9 | 1:10:1.3 | **2p** | 1:3 | 0.50 M | 24 h | 30% |

*isolated yield

## 5. Computational Details

All density functional theory (DFT) calculations were performed using the Amsterdam Density Functional (AMS2023.101) software package.[2] The generalized gradient approximation (GGA) exchange-correlation functional BLYP was used for all computations, which consists of the Becke exchange, and the Lee–Yang–Parr (LYP) correlation functional.[3] In addition, dispersion effects have been included using the D3(BJ) approximation by Grimme *et al*.[4] Scalar relativistic effects are accounted for using the zeroth-order regular approximation (ZORA).[5] The basis set used, denoted TZ2P, is of triple-z quality for all atoms and has been improved by four sets of polarization functions.[6] The polarization functions are 2p and 3d on H, 3d and 4f on C, P, Cl, and 5p and 4f on Pd. No frozen core approximation has been employed. For all calculations, the accuracies of the fit scheme (Zlm fit) and the integration grid (Becke grid) were set to VERYGOOD.[7] No symmetry constraints were used for all computations. To account for bulk solvation of DCM, we used the conductor-like screening model (COSMO).[8] All calculated stationary points have been verified by performing a vibrational analysis calculation,[9] to be energy minima (no imaginary frequencies) or transition states (only one imaginary frequency). The character of the normal mode associated with the imaginary frequency of the transition state has been inspected to ensure that it is associated with the reaction of interest. The optimized structures were illustrated using CYLview.[10] Conformer searches were performed using the algorithm of the RDKit, 5000 conformers of the given system were randomly generated. The generated conformers were compared to each other via the root mean square (RMS), and structures that were too similar were removed. The unique conformers were optimized by a universal force field (UFF). Again, the obtained conformers were compared by RMS, and duplicates were removed. The final conformers were sorted according to their energy. All these steps were automatically executed by the RDKit algorithm.[11] It would be too computationally expensive to optimize all generated conformers at a DFT level of accuracy. Therefore, several conformers were manually selected for re-optimization with DFT. For the thermochemistry calculations, we used a standard approach whereby the geometries were optimized, and the vibrational frequencies were obtained through numerical differentiation of the analytical gradient. Enthalpies at 298.15 K and 1 atm ($\Delta H$) were calculated from the electronic bond energies and vibrational frequencies by using a standard thermochemistry relation for an ideal gas [Eq. S1].

$$\Delta H = \Delta E_{\text{trans}} + \Delta E_{\text{rot}} + \Delta E_{\text{vib,0}} + \Delta\ (\Delta E_{\text{vib,298}}) + \Delta\ (p\text{V}) \qquad \text{(S1)}$$

$\Delta E_{\text{trans,298}}$, $\Delta E_{\text{rot,298}}$, and $\Delta E_{\text{vib,0}}$ are the differences between the reactants in the translational, rotational, and zero-point vibrational energy, respectively, whereas $\Delta E_{\text{vib,298}}$ takes the vibrational energy change upon going from 0 to 298.15 K into account. Entropies were corrected according to the approach of Martin-

Hay-Pratt.[12] This is important because the ideal gas approximation ignores the solvent suppression effect on the rotational and translational degrees of freedom of the solute, which can lead to a large overestimation of the entropy contributions to the Gibbs energy in solution. Finally, the change of the Gibbs energy ($\Delta G$) in solution was then calculated according to Eq. S2.

$$\Delta G = \Delta H - T\Delta S \tag{S2}$$

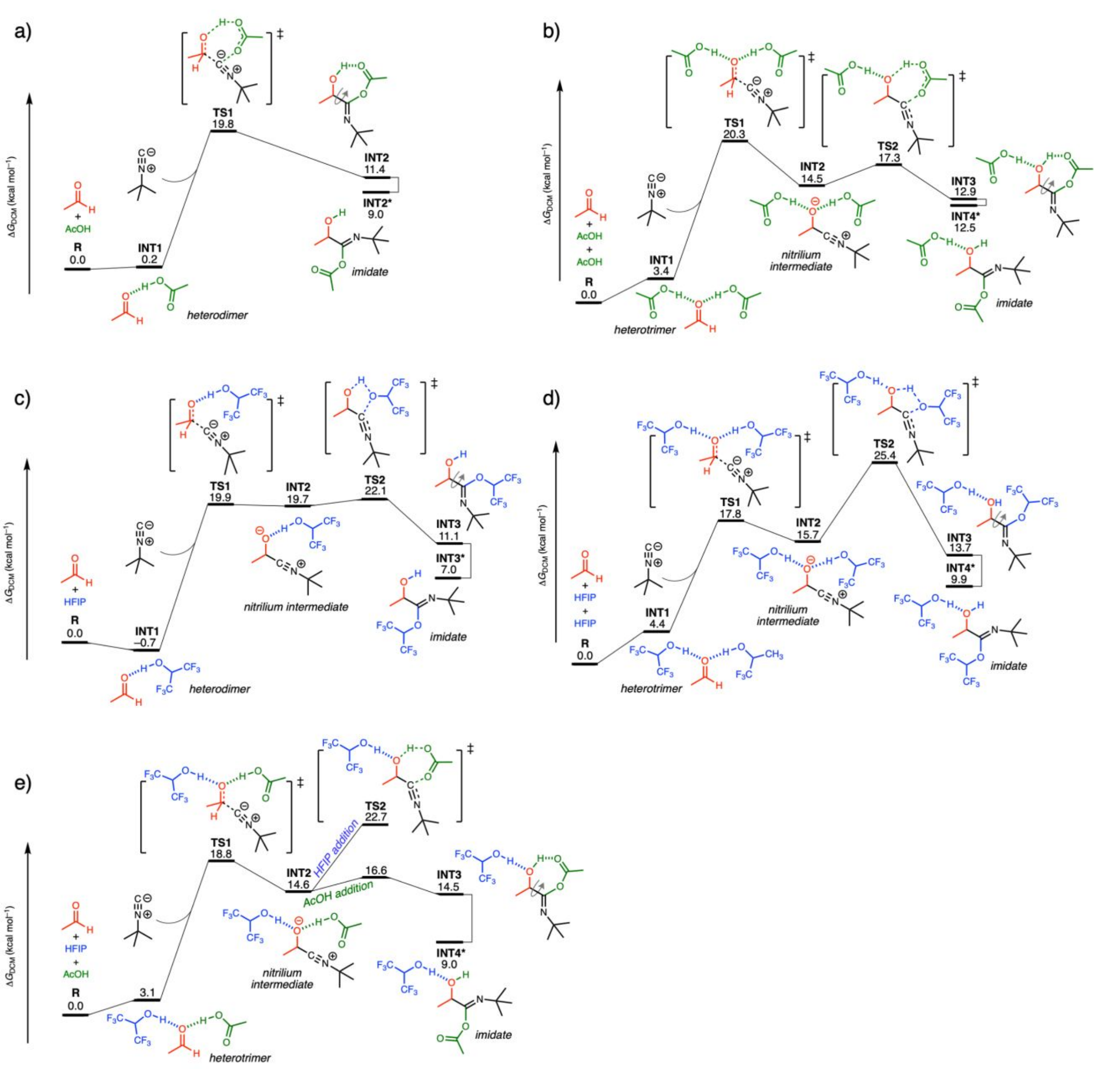


**Figure S17.** Reaction profiles ($\Delta G_{DCM}$ in kcal mol$^{-1}$) of a Passerini-type reaction between acetaldehyde, *tert*-butyl isocyanide with a) 1, b) 2 AcOH molecule(s), or c) 1, d) 2 HFIP molecule(s), or e) 1 AcOH and 1 HFIP molecule in dichloromethane.

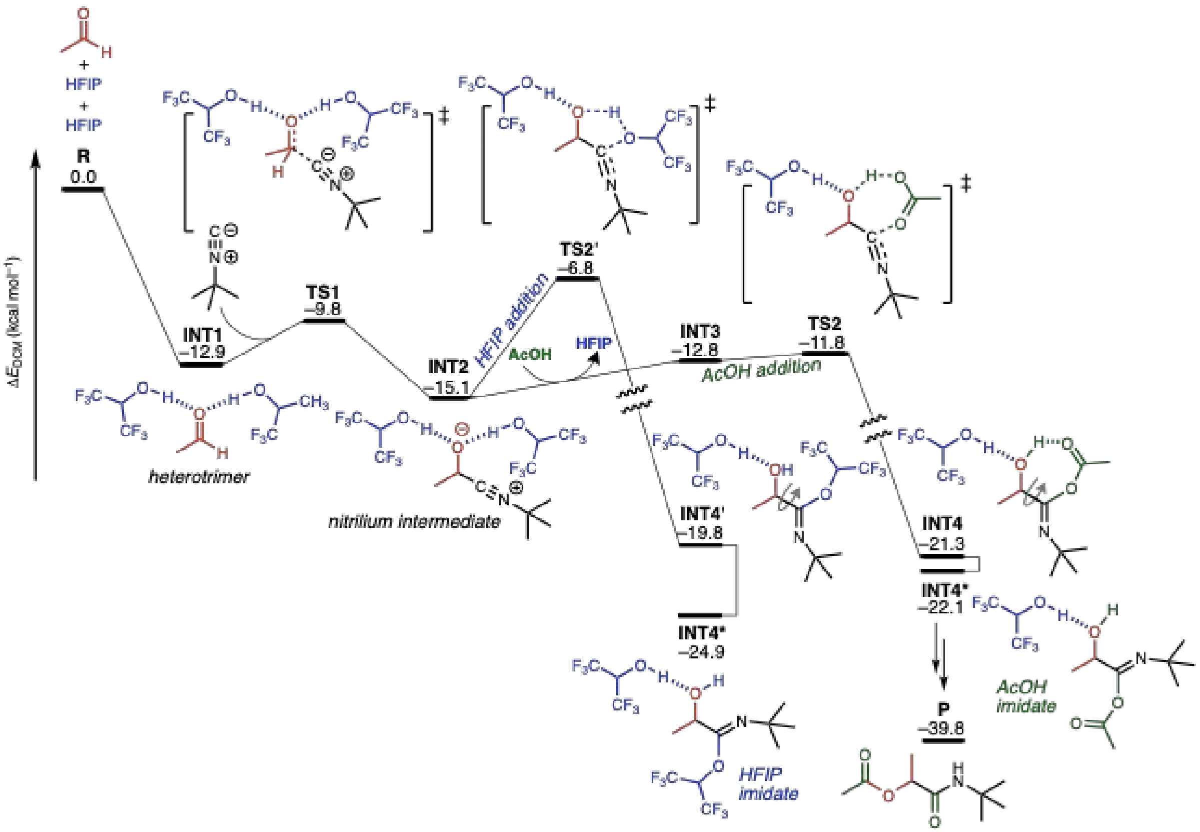


**Figure S18.** Reaction profile ($\Delta E_{DCM}$ in kcal mol$^{-1}$) of a Passerini-type reaction between acetaldehyde, *tert*-butyl isocyanide, AcOH, and HFIP in dichloromethane.

**Table S6.** Cartesian coordinates (in Å), energies (in kcal mol$^{-1}$), and the number of imaginary frequencies of all stationary points and transition states, computed at COSMO(DCM)-ZORA-BLYP-BJ(D3)/TZ2P.

**$Me_3CNC$**
***E*** = -1900.20
***H*** = -1815.92
***G*** = -1840.30
***N*imag** = 0

| | | | |
|---|---|---|---|
| H | 2.65538550 | -4.14744342 | -0.15237119 |
| H | 4.25643209 | -4.91918171 | -0.06279161 |
| H | 3.34557268 | -4.93863683 | -1.59146527 |
| C | 5.55412629 | -3.28550433 | -1.91451082 |
| H | 6.02037202 | -2.34294757 | -2.21797855 |
| H | 6.26114119 | -3.84393868 | -1.29281196 |
| H | 5.32982186 | -3.87502791 | -2.80896352 |
| C | 2.60977230 | -1.67858830 | -2.63449298 |
| N | 3.34936323 | -2.27695055 | -1.95045666 |
| C | 4.26889526 | -3.02005211 | -1.10096307 |
| C | 4.56803011 | -2.15474351 | 0.14241187 |
| H | 5.25918100 | -2.69620651 | 0.79617844 |
| H | 3.64833741 | -1.94625324 | 0.69791708 |
| C | 3.58049246 | -4.34376742 | -0.70330113 |
| H | 5.02954063 | -1.20620190 | -0.14940764 |

**AcOH**
***E*** = -1043.00
***H*** = -1002.36
***G*** = -1023.35
***N*imag** = 0

| | | | |
|---|---|---|---|
| C | 2.06344818 | -1.83084907 | -2.32020103 |
| C | 2.97429765 | -0.75651333 | -1.78353566 |
| O | 3.66979257 | -0.00396073 | -2.45069544 |
| O | 2.94806981 | -0.70276958 | -0.41694372 |
| H | 2.10179264 | -1.83835411 | -3.40999854 |
| H | 1.03764566 | -1.64885591 | -1.98072600 |
| H | 2.37330049 | -2.80564227 | -1.92688057 |
| H | 3.56069011 | 0.01170988 | -0.13843121 |

**HFIP**
***E*** = -1459.99
***H*** = -1417.57
***G*** = -1444.90
***N*imag** = 0

| | | | |
|---|---|---|---|
| O | -0.52948279 | 0.13872144 | -4.09596361 |
| C | 0.77185763 | -1.91810800 | -3.89464737 |

| | | | |
|---|---|---|---|
| F | 0.83171896 | -3.23964528 | -4.21270569 |
| F | 1.85221827 | -1.30720317 | -4.45790851 |
| F | 0.90094381 | -1.80581001 | -2.53739162 |
| H | -0.52949543 | -1.33472199 | -5.49449662 |
| H | -0.52948137 | 0.25475618 | -3.12539886 |
| C | -1.83083356 | -1.91808841 | -3.89461403 |
| F | -2.91118483 | -1.30731424 | -4.45803620 |
| F | -1.95999533 | -1.80556044 | -2.53738428 |
| F | -1.89063532 | -3.23968636 | -4.21244014 |
| C | -0.52948749 | -1.24066556 | -4.40502714 |

**Acetaldehyde**
***E*** = -874.14
***H*** = -837.49
***G*** = -856.26
***N*imag** = 0

| | | | |
|---|---|---|---|
| C | 0.08572097 | 0.18184814 | 0.86146898 |
| O | -0.13090483 | 0.27289961 | -0.34120063 |
| H | 1.82126179 | -1.09255906 | 0.71328175 |
| H | -0.57919326 | 0.70507612 | 1.58395755 |
| C | 1.20920826 | -0.60106086 | 1.47426878 |
| H | 1.82433570 | 0.07709751 | 2.08286862 |
| H | 0.78896637 | -1.34307715 | 2.16805749 |

**AcOH-INT1**
***E*** = -1924.06
***H*** = -1845.45
***G*** = -1875.93
***N*imag** = 0

| | | | |
|---|---|---|---|
| C | -4.35234004 | 0.88406232 | -1.48379832 |
| C | -3.04699677 | 0.58929713 | -0.78105767 |
| O | -2.91531067 | 0.48776721 | 0.43701532 |
| O | -2.01759200 | 0.44805879 | -1.64515996 |
| H | -5.15955467 | 0.96912632 | -0.75482809 |
| H | -4.57615918 | 0.08553697 | -2.20013837 |
| H | -4.26143166 | 1.81799026 | -2.05040110 |
| H | -1.16633590 | 0.25724529 | -1.13309514 |
| C | 0.33314324 | -0.12829884 | 0.80957962 |
| O | 0.33123220 | -0.06277099 | -0.42182800 |
| H | 2.43947614 | -0.53222485 | 1.00716319 |
| H | -0.61800849 | 0.02074660 | 1.35472922 |
| C | 1.55014004 | -0.39960932 | 1.62824187 |
| H | 1.69211974 | 0.43082494 | 2.33460197 |
| H | 1.37004502 | -1.29398345 | 2.24178783 |

**AcOH-TS1**

***E*** = -3814.66
***H*** = -3651.41
***G*** = -3693.17
***N*imag** = -323.5428

| | | | |
|---|---|---|---|
| C | -4.00104069 | -0.51442515 | 1.41766115 |
| C | -2.52556907 | -0.42407464 | 1.07651949 |
| O | -1.63346721 | -0.37989661 | 1.92927239 |
| O | -2.29284875 | -0.42337675 | -0.23855438 |
| H | -4.14879247 | -0.40295897 | 2.49330812 |
| H | -4.39000094 | -1.48798163 | 1.09416479 |
| H | -4.56219653 | 0.25564701 | 0.87764920 |
| H | -1.24579436 | -0.40453882 | -0.45687581 |
| C | 1.03880447 | -0.55467070 | 0.00240192 |
| O | 0.13729634 | -0.45379120 | -0.93141310 |
| H | 2.71734390 | -1.12003044 | -1.24896046 |
| H | 0.81884368 | -0.05409260 | 0.96138643 |
| C | 2.50319748 | -0.45804349 | -0.40401921 |
| H | 2.69382438 | 0.57671304 | -0.71405389 |
| H | 3.16345005 | -0.70361895 | 0.43467393 |
| H | -2.35390975 | -3.24471553 | 0.06918225 |
| H | -2.55567820 | -5.00256327 | 0.27027950 |
| H | -1.37346441 | -4.36908792 | -0.89914261 |
| C | 0.03636816 | -5.55609978 | 1.17363257 |
| H | 0.81526541 | -5.55005669 | 1.94211290 |
| H | -0.64988513 | -6.38435342 | 1.37471539 |
| H | 0.49983757 | -5.71290547 | 0.19485470 |
| C | 0.83449842 | -2.22278498 | 0.68902852 |
| N | 0.16677728 | -3.14494833 | 0.92349796 |
| C | -0.76201121 | -4.23685976 | 1.19538670 |
| C | -1.38271049 | -3.97202315 | 2.58375975 |
| H | -2.11024183 | -4.76106629 | 2.79677236 |
| H | -1.88741872 | -3.00238316 | 2.60332100 |
| C | -1.83097998 | -4.20486367 | 0.08104295 |
| H | -0.61138814 | -3.98499920 | 3.35978779 |

**AcOH-INT2**
***E*** = -3829.73
***H*** = -3663.84
***G*** = -3701.60
***N*imag** = 0

| | | | |
|---|---|---|---|
| C | -2.75016069 | 1.71985403 | -1.23149886 |
| C | -1.30213848 | 1.30922255 | -1.19879361 |
| O | -0.95299445 | 0.83568182 | 0.03506868 |
| O | -0.52785803 | 1.36321960 | -2.14670530 |
| H | -2.91003269 | 2.54429849 | -0.52727841 |
| H | -3.02294465 | 2.03251904 | -2.24011961 |

| | | | |
|---|---|---|---|
| H | -3.38206069 | 0.88516472 | -0.90920594 |
| H | 1.37408162 | 1.36066217 | -1.83457453 |
| C | 1.45314178 | 1.54545262 | 0.10938869 |
| O | 2.10469130 | 1.38997387 | -1.17864170 |
| H | 3.24532545 | 2.34004786 | 0.99281850 |
| H | 0.91511674 | 2.50368111 | 0.13102747 |
| C | 2.52931684 | 1.53507632 | 1.18860928 |
| H | 2.07844283 | 1.69574152 | 2.17347857 |
| H | 3.05092543 | 0.57394183 | 1.19422120 |
| C | -0.91288701 | -2.22338429 | -0.45370142 |
| H | -0.17021325 | -2.34562914 | -1.24986924 |
| H | -1.45498529 | -3.16900876 | -0.34247573 |
| H | -1.62928002 | -1.45596598 | -0.75549199 |
| C | 0.42548390 | 0.42641785 | 0.30150785 |
| N | 0.71058632 | -0.72019009 | 0.71291276 |
| C | -0.21896169 | -1.87045699 | 0.88227910 |
| C | 0.66056430 | -3.05802902 | 1.32898567 |
| H | 1.41343473 | -3.28361698 | 0.56543348 |
| H | 0.04602303 | -3.95100062 | 1.48937784 |
| C | -1.25760921 | -1.55522406 | 1.98364532 |
| H | 1.17979353 | -2.81596168 | 2.26280730 |
| H | -1.85833818 | -2.44962108 | 2.18408473 |
| H | -0.75193891 | -1.26666138 | 2.91205590 |
| H | -1.92668668 | -0.74564903 | 1.68178479 |

**AcOH-INT2***

***E*** = -3831.53

***H*** = -3665.75

***G*** = -3704.01

***N*imag** = 0

| | | | |
|---|---|---|---|
| H | -2.78952020 | -8.02542116 | 2.84566849 |
| H | 1.33492864 | -7.08700664 | 1.86629950 |
| H | 0.63255741 | -8.67137629 | 1.45833092 |
| H | 0.12894843 | -7.21492607 | 0.56807917 |
| C | -4.81289373 | -6.42817332 | 5.05663231 |
| C | -4.00702024 | -5.85094998 | 3.92475664 |
| O | -2.65660740 | -5.85507059 | 4.24345845 |
| O | -4.42798391 | -5.44829495 | 2.85809940 |
| H | -5.86708451 | -6.45953326 | 4.77894636 |
| H | -4.67773830 | -5.81406909 | 5.95399559 |
| H | -4.45363474 | -7.43627197 | 5.29143505 |
| H | -0.38586106 | -4.05971012 | 2.00910492 |
| C | -1.76199337 | -3.73544367 | 3.33084703 |
| O | -0.84458898 | -3.24943688 | 2.33369230 |
| H | -1.40288642 | -2.08445157 | 4.67632724 |
| H | -2.77458545 | -3.40764310 | 3.05957081 |
| C | -1.40118897 | -3.17802300 | 4.71914059 |

| | | | |
|---|---|---|---|
| H | -2.13071621 | -3.49879805 | 5.47002799 |
| H | -0.40442337 | -3.52044560 | 5.01914534 |
| C | -0.27403960 | -7.89605506 | 3.96231350 |
| H | -1.06642631 | -7.76650142 | 4.70442581 |
| H | 0.62251984 | -7.37016877 | 4.30923646 |
| H | -0.03849316 | -8.96347277 | 3.88589329 |
| C | -1.74175176 | -5.25763100 | 3.32278855 |
| N | -0.89415958 | -5.88780664 | 2.64382726 |
| C | -0.70830286 | -7.36407289 | 2.57751011 |
| C | -1.99804241 | -8.06463678 | 2.09368139 |
| H | -1.78129363 | -9.11835663 | 1.88658136 |
| H | -2.36536113 | -7.60065066 | 1.17212168 |
| C | 0.41978059 | -7.60068907 | 1.55169178 |

**HFIP-INT1**
***E*** = -2341.10
***H*** = -2260.08
***G*** = -2298.44
***N*imag** = 0

| | | | |
|---|---|---|---|
| F | -2.75269734 | -1.49970499 | 0.29470964 |
| H | -3.81246746 | -0.13077382 | -2.53863266 |
| H | -1.14553322 | -0.28506785 | -1.42986381 |
| C | -3.32530059 | 1.22308027 | -0.96209635 |
| F | -2.99761810 | 2.23815666 | -1.81161354 |
| F | -2.52681936 | 1.34658448 | 0.14278844 |
| F | -4.61383105 | 1.42183815 | -0.56351120 |
| C | -3.12788794 | -0.13971052 | -1.68372284 |
| C | 0.28554967 | -0.25107481 | 0.77394707 |
| O | 0.26806382 | -0.32678705 | -0.45433450 |
| H | 2.42475116 | -0.37198214 | 0.97951453 |
| H | -0.66745478 | -0.15676820 | 1.32953177 |
| C | 1.52899155 | -0.27633202 | 1.59779705 |
| H | 1.57087263 | 0.64225727 | 2.20048747 |
| H | 1.46146232 | -1.10801139 | 2.31359261 |
| O | -1.81933002 | -0.27670856 | -2.17073477 |
| C | -3.53996125 | -1.36244168 | -0.81640716 |
| F | -4.83575346 | -1.29439914 | -0.39792486 |
| F | -3.40527724 | -2.50446293 | -1.54948750 |

**HFIP-TS1**
***E*** = -4232.36
***H*** = -4067.06
***G*** = -4114.62
***N*imag** = -303.5539

| | | | |
|---|---|---|---|
| F | 3.45159201 | -3.07837713 | 0.12561961 |
| F | 5.41154681 | -3.49249257 | -0.78406478 |

| | | | |
|---|---|---|---|
| F | 3.59740944 | -3.45581833 | -2.03609366 |
| C | 4.39020380 | -1.31798323 | -1.17879144 |
| C | 2.63751436 | -0.05941105 | 2.11145126 |
| O | 3.16363385 | 0.57133470 | 1.10262720 |
| H | 1.26873059 | 1.49195211 | 2.77003824 |
| H | 3.20186068 | -0.91658780 | 2.52002223 |
| C | 1.96411695 | 0.76667413 | 3.20406074 |
| H | 1.43527807 | 0.12603654 | 3.91798588 |
| H | 2.75359939 | 1.31322915 | 3.73415200 |
| O | 4.98561342 | -0.75157834 | -0.05341480 |
| C | 5.21521914 | -0.95299670 | -2.44149764 |
| F | 4.67386504 | -1.47132006 | -3.58680304 |
| F | 5.24390701 | 0.40444229 | -2.58763904 |
| H | 0.68721194 | -3.99364971 | -0.33073350 |
| H | -0.96063485 | -4.01139027 | -1.00022597 |
| H | -0.71840507 | -3.83043270 | 0.75322971 |
| C | -1.69013984 | -1.42065750 | -0.22585062 |
| H | -1.65088423 | -0.33317580 | -0.33949339 |
| H | -2.36601014 | -1.82991282 | -0.98263752 |
| H | -2.08231555 | -1.66424951 | 0.76598935 |
| C | 1.26457545 | -1.04309108 | 1.43231709 |
| N | 0.58254865 | -1.48359054 | 0.60105825 |
| C | -0.29246661 | -2.04262678 | -0.42768728 |
| C | 0.30205036 | -1.65873999 | -1.79924673 |
| H | -0.36999322 | -2.02063824 | -2.58296157 |
| H | 1.27956360 | -2.12603869 | -1.94474369 |
| C | -0.31740930 | -3.57276808 | -0.23145904 |
| H | 0.39817957 | -0.57320940 | -1.89384391 |
| F | 6.50767376 | -1.38222165 | -2.36723511 |
| H | 3.37611851 | -0.93055332 | -1.36116074 |
| C | 4.22738059 | -2.85148263 | -0.98025420 |
| H | 4.25236592 | -0.19291662 | 0.44528280 |

**HFIP-INT2**
***E*** = -4234.92
***H*** = -4070.83
***G*** = -4114.87
***N*imag** = 0

| | | | |
|---|---|---|---|
| F | 3.42894091 | -3.02486963 | -0.02068006 |
| F | 5.42567613 | -3.48267379 | -0.82318288 |
| F | 3.69952633 | -3.31350054 | -2.18393258 |
| C | 4.52153148 | -1.24533828 | -1.17703277 |
| C | 2.60796496 | -0.13064512 | 2.03585563 |
| O | 3.26722260 | 0.59513898 | 1.07053349 |
| H | 1.34037525 | 1.48595537 | 2.76332574 |
| H | 3.21746857 | -0.94022270 | 2.48609205 |
| C | 2.00113045 | 0.71661034 | 3.17511262 |

| | | | |
|---|---|---|---|
| H | 1.44434151 | 0.09356208 | 3.88460964 |
| H | 2.83031061 | 1.20166524 | 3.69924024 |
| O | 5.04071719 | -0.74959918 | 0.00732237 |
| C | 5.45097950 | -0.86992688 | -2.36345497 |
| F | 4.98271410 | -1.32608094 | -3.56826500 |
| F | 5.54049702 | 0.49025504 | -2.45815159 |
| H | 0.70870136 | -3.91326692 | -0.15282544 |
| H | -0.97680985 | -3.92449323 | -0.72020998 |
| H | -0.62402518 | -3.63222370 | 0.99884917 |
| C | -1.58753613 | -1.26435345 | -0.08928221 |
| H | -1.52388473 | -0.18901930 | -0.27959001 |
| H | -2.31705998 | -1.70134953 | -0.77735363 |
| H | -1.92768281 | -1.43030640 | 0.93702541 |
| C | 1.49563544 | -0.87029613 | 1.32707241 |
| N | 0.72733560 | -1.34461568 | 0.60579832 |
| C | -0.22501279 | -1.94550526 | -0.33113766 |
| C | 0.30565150 | -1.67006334 | -1.75384262 |
| H | -0.41324236 | -2.07500304 | -2.47202124 |
| H | 1.26800032 | -2.16290730 | -1.91410315 |
| C | -0.27601794 | -3.45593833 | -0.02303008 |
| H | 0.41101544 | -0.59584549 | -1.93049435 |
| F | 6.72014400 | -1.34971715 | -2.21247884 |
| H | 3.53709931 | -0.81491647 | -1.42840557 |
| C | 4.28541793 | -2.77937708 | -1.06521126 |
| H | 4.25770125 | -0.14395625 | 0.49743529 |

**HFIP-TS2**

***E*** = -4232.16
***H*** = -4066.52
***G*** = -4112.46
***N*imag** = -46.9140

| | | | |
|---|---|---|---|
| F | -3.15699389 | -0.11305056 | -3.84088709 |
| H | -3.15000087 | -1.88639269 | -1.02876193 |
| H | -0.48669825 | -0.70544733 | -2.32573268 |
| C | -3.55061975 | -2.96776846 | -2.81347747 |
| F | -3.06545606 | -4.16152461 | -2.34020579 |
| F | -3.35428098 | -2.96867524 | -4.16645234 |
| F | -4.90916566 | -2.99532656 | -2.60369428 |
| C | -2.82316830 | -1.78927179 | -2.08893502 |
| C | 0.17907242 | -0.14660285 | -0.60239804 |
| O | 0.30023845 | -0.11253605 | -2.00991042 |
| H | 2.32108104 | -0.17749069 | -0.22306797 |
| H | -0.70378999 | 0.41322734 | -0.25075777 |
| C | 1.44338219 | 0.39996611 | 0.08085691 |
| H | 1.56689000 | 1.43928811 | -0.23698938 |
| H | 1.34011656 | 0.37080398 | 1.16959409 |
| H | -1.95270594 | -3.51064949 | 1.96571038 |

| | | | |
|---|---|---|---|
| H | -1.90624520 | -5.19489165 | 1.39716672 |
| H | -2.41013382 | -3.90373898 | 0.28497617 |
| C | 0.03674254 | -4.82443746 | -0.60606825 |
| H | 1.08357962 | -4.70778445 | -0.90155598 |
| H | -0.15330570 | -5.87999848 | -0.38926459 |
| H | -0.60896461 | -4.49875940 | -1.42509986 |
| C | -0.05365029 | -1.55194297 | -0.16514996 |
| N | -0.05875650 | -2.60207976 | 0.31718073 |
| C | -0.26311737 | -4.00975899 | 0.67059610 |
| C | 0.70836661 | -4.34970668 | 1.81704320 |
| H | 0.57641477 | -5.40297881 | 2.08154892 |
| H | 0.50006364 | -3.73728815 | 2.69953048 |
| C | -1.73594848 | -4.15366128 | 1.10781701 |
| H | 1.74581928 | -4.19353264 | 1.50683558 |
| O | -1.48147956 | -1.86849852 | -2.28859310 |
| C | -3.41005075 | -0.40898529 | -2.53060502 |
| F | -4.76607110 | -0.29104679 | -2.34554110 |
| F | -2.83121437 | 0.58744061 | -1.78052424 |

**HFIP-TS2**
***E*** = -4232.16
***H*** = -4066.52
***G*** = -4112.46
***N*imag** = -46.9140

| | | | |
|---|---|---|---|
| F | -3.15699389 | -0.11305056 | -3.84088709 |
| H | -3.15000087 | -1.88639269 | -1.02876193 |
| H | -0.48669825 | -0.70544733 | -2.32573268 |
| C | -3.55061975 | -2.96776846 | -2.81347747 |
| F | -3.06545606 | -4.16152461 | -2.34020579 |
| F | -3.35428098 | -2.96867524 | -4.16645234 |
| F | -4.90916566 | -2.99532656 | -2.60369428 |
| C | -2.82316830 | -1.78927179 | -2.08893502 |
| C | 0.17907242 | -0.14660285 | -0.60239804 |
| O | 0.30023845 | -0.11253605 | -2.00991042 |
| H | 2.32108104 | -0.17749069 | -0.22306797 |
| H | -0.70378999 | 0.41322734 | -0.25075777 |
| C | 1.44338219 | 0.39996611 | 0.08085691 |
| H | 1.56689000 | 1.43928811 | -0.23698938 |
| H | 1.34011656 | 0.37080398 | 1.16959409 |
| H | -1.95270594 | -3.51064949 | 1.96571038 |
| H | -1.90624520 | -5.19489165 | 1.39716672 |
| H | -2.41013382 | -3.90373898 | 0.28497617 |
| C | 0.03674254 | -4.82443746 | -0.60606825 |
| H | 1.08357962 | -4.70778445 | -0.90155598 |
| H | -0.15330570 | -5.87999848 | -0.38926459 |
| H | -0.60896461 | -4.49875940 | -1.42509986 |
| C | -0.05365029 | -1.55194297 | -0.16514996 |

| | | | |
|---|---|---|---|
| N | -0.05875650 | -2.60207976 | 0.31718073 |
| C | -0.26311737 | -4.00975899 | 0.67059610 |
| C | 0.70836661 | -4.34970668 | 1.81704320 |
| H | 0.57641477 | -5.40297881 | 2.08154892 |
| H | 0.50006364 | -3.73728815 | 2.69953048 |
| C | -1.73594848 | -4.15366128 | 1.10781701 |
| H | 1.74581928 | -4.19353264 | 1.50683558 |
| O | -1.48147956 | -1.86849852 | -2.28859310 |
| C | -3.41005075 | -0.40898529 | -2.53060502 |
| F | -4.76607110 | -0.29104679 | -2.34554110 |
| F | -2.83121437 | 0.58744061 | -1.78052424 |

**HFIP-INT3**
***E*** = -4246.35
***H*** = -4078.65
***G*** = -4123.47
***N*imag** = 0

| | | | |
|---|---|---|---|
| H | -0.00343082 | -1.79037908 | -2.16116646 |
| H | 1.11220806 | -2.91711983 | -2.96445659 |
| H | -0.76913262 | 1.35269334 | 0.98325816 |
| H | 1.01410927 | -1.22956129 | -3.50854329 |
| C | 0.97996373 | -1.88219417 | -2.62867841 |
| H | 2.79216042 | -2.05511027 | 0.36199645 |
| H | 1.05674286 | -2.34035322 | 0.09922309 |
| H | -0.70540283 | 2.26075865 | -1.31337168 |
| C | 1.12292363 | 2.04836536 | -0.49951775 |
| O | 0.17507485 | 2.66406492 | -1.42218319 |
| H | 3.22251627 | 2.27696209 | -0.05961185 |
| H | 0.82817209 | 2.28540058 | 0.53230050 |
| C | 2.48331881 | 2.68027683 | -0.75755404 |
| H | 2.40765075 | 3.76222073 | -0.60891559 |
| H | 2.81561932 | 2.46879660 | -1.77698132 |
| C | 3.47921845 | -1.72218026 | -2.32782311 |
| H | 4.29284989 | -1.44890384 | -1.64643748 |
| H | 3.55593915 | -1.09510102 | -3.22303101 |
| H | 3.60490106 | -2.77066284 | -2.62090080 |
| C | 1.12237147 | 0.53516746 | -0.71007502 |
| N | 2.04831748 | -0.06426340 | -1.30928642 |
| C | 2.11185219 | -1.51205308 | -1.64151201 |
| C | 2.04248284 | -2.38426463 | -0.36643225 |
| H | 2.25513072 | -3.42693619 | -0.62859093 |
| C | -0.66995449 | -0.51927282 | 2.00011008 |
| F | 0.64167966 | -0.38354142 | 2.35165940 |
| F | -0.92016169 | -1.84548719 | 1.83406085 |
| F | -1.42764775 | -0.07356315 | 3.03975697 |
| C | -0.92810808 | 0.30617166 | 0.71131604 |
| O | -0.06894627 | -0.16232830 | -0.31600592 |

| | | | |
|---|---|---|---|
| C | -2.37493456 | 0.20594037 | 0.16895834 |
| F | -3.29169342 | 0.58180374 | 1.09801069 |
| F | -2.49941440 | 1.05540020 | -0.90359141 |
| F | -2.69036969 | -1.04064309 | -0.26120513 |

**HFIP-INT3***
***E*** = -4250.98
***H*** = -4083.12
***G*** = -4127.57
***N*imag** = 0

| | | | |
|---|---|---|---|
| H | -0.16511501 | -1.98014671 | -1.52947586 |
| H | 0.65190752 | -3.04422276 | -2.69047496 |
| H | -0.61703962 | 1.53510385 | 0.96540443 |
| H | 0.14136378 | -1.41827120 | -3.18908911 |
| C | 0.54639420 | -2.00562909 | -2.35739174 |
| H | 2.63227293 | -3.26739628 | -0.95726671 |
| H | 1.77333865 | -2.16255898 | 0.13707422 |
| H | 2.31670053 | 1.80919397 | -2.21444599 |
| C | 1.26118059 | 2.09372039 | -0.62721907 |
| O | 1.94526194 | 2.61507507 | -1.77769307 |
| H | 3.01949047 | 2.00827914 | 0.64136186 |
| H | 0.26215717 | 2.54804431 | -0.61411629 |
| C | 2.02105173 | 2.45777051 | 0.66266009 |
| H | 2.12021967 | 3.54569634 | 0.72261035 |
| H | 1.50291510 | 2.10878156 | 1.56188540 |
| C | 2.92100581 | -1.61056732 | -3.11045524 |
| H | 2.55850256 | -1.06460914 | -3.98882328 |
| H | 3.90245040 | -1.20811744 | -2.83567305 |
| H | 3.03758365 | -2.66646120 | -3.37868429 |
| C | 1.14372420 | 0.57356617 | -0.78768528 |
| N | 1.85618361 | -0.00105067 | -1.65347712 |
| C | 1.93046119 | -1.45932663 | -1.93694217 |
| C | 2.47280284 | -2.21390434 | -0.70117420 |
| H | 3.43232627 | -1.78845322 | -0.38597890 |
| C | -0.81835603 | -0.21025142 | 2.18239448 |
| F | -0.92032569 | -1.56254669 | 2.10057682 |
| F | -1.87367190 | 0.25181882 | 2.90618730 |
| F | 0.32173543 | 0.07768030 | 2.87317244 |
| C | -0.75531259 | 0.46608962 | 0.78982443 |
| O | 0.32816069 | -0.14759080 | 0.10049373 |
| C | -2.05282291 | 0.30996109 | -0.05118739 |
| F | -3.09885163 | 0.96123680 | 0.52454746 |
| F | -1.84766646 | 0.85790308 | -1.28505808 |
| F | -2.40823255 | -0.98923357 | -0.23196951 |

**2AcOH-INT1**
***E*** = -2972.42

***H*** = -2852.34
***G*** = -2892.68
***N*imag** = 0

| | | | |
|---|---|---|---|
| H | 5.40336922 | -4.54729158 | -0.38606315 |
| H | 5.81467112 | -3.20914175 | -1.50361159 |
| H | 4.33768533 | -4.14302479 | -1.76767033 |
| H | 3.21602995 | -1.12558665 | -0.21423496 |
| C | 2.27051252 | -0.25146771 | 1.95901525 |
| O | 2.33760199 | 0.01157474 | 0.75097166 |
| H | 0.89096787 | -0.02805079 | 3.54385169 |
| H | 2.75932404 | -1.17535742 | 2.31381236 |
| C | 1.58321435 | 0.59914433 | 2.96515010 |
| H | 2.33843486 | 0.95477253 | 3.68225145 |
| H | 1.05686590 | 1.43829190 | 2.50590970 |
| C | 4.99999439 | -3.73677243 | -0.99466296 |
| C | 4.23224431 | -2.76915166 | -0.12539099 |
| O | 4.08145453 | -2.88226930 | 1.08855558 |
| O | 3.71376765 | -1.73934823 | -0.83531831 |
| O | 1.58742515 | 2.28673469 | -0.60533743 |
| H | -0.76042987 | 4.61253531 | -0.73706972 |
| H | 0.06819684 | 3.84562963 | -2.12711513 |
| O | -0.27231621 | 2.49907745 | 0.67562162 |
| C | 0.43975572 | 2.90726564 | -0.23665347 |
| C | 0.15991986 | 4.13309293 | -1.07339664 |
| H | 0.99871813 | 4.83401220 | -0.99542734 |
| H | 1.73830385 | 1.48252728 | -0.02901819 |

**2AcOH-TS1**
***E*** = -4867.67
***H*** = -4662.61
***G*** = -4712.58
***N*imag** = -373.5827

| | | | |
|---|---|---|---|
| C | 4.92138809 | -3.49152230 | -0.27806757 |
| H | 5.90879814 | -3.18487981 | -0.64040895 |
| H | 4.28377948 | -3.63821175 | -1.15816582 |
| C | 0.63043498 | 2.12557782 | -0.59646767 |
| C | 2.45418123 | 0.29761283 | 2.43602402 |
| O | 3.04577720 | 0.64299923 | 1.33285979 |
| H | 1.00403696 | 1.89577814 | 2.56500649 |
| H | 2.97343764 | -0.45083700 | 3.04966455 |
| C | 1.68898726 | 1.34765550 | 3.21711424 |
| H | 1.13251999 | 0.89272551 | 4.04222370 |
| H | 2.42576454 | 2.04878766 | 3.62965324 |
| O | 1.97209609 | 2.19090232 | -0.48843055 |
| C | 0.10190015 | 2.95479115 | -1.74763113 |
| H | -0.98875211 | 2.92485216 | -1.76168140 |

| | | | |
|---|---|---|---|
| H | 0.44914599 | 3.98971435 | -1.65496920 |
| H | 1.66159637 | -3.93927145 | 0.20851813 |
| H | 0.22075010 | -4.44873713 | -0.70367471 |
| H | 0.13186877 | -4.24911389 | 1.06222202 |
| C | -1.38124329 | -2.22566600 | -0.09744164 |
| H | -1.65391159 | -1.17216693 | -0.20863563 |
| H | -1.78023566 | -2.78828866 | -0.94706242 |
| H | -1.82472278 | -2.61674965 | 0.82336975 |
| C | 1.10734799 | -0.92508760 | 1.87576479 |
| N | 0.66068157 | -1.60938240 | 1.05016522 |
| C | 0.15302471 | -2.38390340 | -0.07465461 |
| C | 0.79607858 | -1.79181968 | -1.34873841 |
| H | 0.44800779 | -2.36468420 | -2.21363742 |
| H | 1.88701908 | -1.84937029 | -1.29903854 |
| C | 0.57316607 | -3.85213352 | 0.14302540 |
| H | 0.50152636 | -0.74646552 | -1.47151769 |
| H | 0.49515500 | 2.56119276 | -2.69268570 |
| O | 4.25710559 | -1.21990211 | -0.02869118 |
| H | 2.30494807 | 1.59761166 | 0.28008798 |
| O | -0.07400245 | 1.45365463 | 0.15423954 |
| O | 3.95357355 | -2.58433219 | 1.75629830 |
| H | 5.00150073 | -4.42560487 | 0.28006619 |
| H | 3.81631888 | -0.51433514 | 0.58200750 |
| C | 4.33509988 | -2.40663896 | 0.60000131 |

**2AcOH-INT2**

***E*** = -4873.43

***H*** = -4668.54

***G*** = -4718.46

***N*imag** = 0

| | | | |
|---|---|---|---|
| H | 4.63141481 | -3.95324887 | -1.71626286 |
| H | 5.80880495 | -2.61034011 | -1.85352052 |
| H | 4.29728948 | -2.54098429 | -2.76375415 |
| H | 3.69406974 | -0.35334490 | 0.09734106 |
| C | 2.42338920 | -0.31768168 | 2.12602200 |
| O | 2.99785775 | 0.43878251 | 1.10471877 |
| H | 1.11161238 | 1.22051297 | 2.92610318 |
| H | 3.13052802 | -1.06691602 | 2.52191641 |
| C | 1.86499290 | 0.51921040 | 3.29273200 |
| H | 1.42309763 | -0.12508400 | 4.06141325 |
| H | 2.70092076 | 1.07316596 | 3.73044943 |
| C | 4.74534697 | -2.87266874 | -1.81949152 |
| C | 4.07585043 | -2.15960264 | -0.66002459 |
| O | 3.45734039 | -2.74908416 | 0.23305092 |
| O | 4.21298910 | -0.83296756 | -0.71002081 |
| H | -2.01284112 | -2.36716845 | 1.35550093 |
| H | -2.46194987 | -2.48651933 | -0.36126622 |

| | | | |
|---|---|---|---|
| H | -1.90018299 | -0.92990112 | 0.30404121 |
| C | 0.04281409 | -1.87371523 | -1.44123350 |
| H | 1.08102828 | -2.11986395 | -1.68005871 |
| H | -0.61252841 | -2.34925916 | -2.17689490 |
| H | -0.09373930 | -0.79024707 | -1.49057446 |
| C | 1.33981564 | -1.14301227 | 1.50824968 |
| N | 0.56696270 | -1.74047908 | 0.89815596 |
| C | -0.33381595 | -2.40431901 | -0.04000626 |
| C | -0.08592850 | -3.92123122 | 0.08026888 |
| H | -0.73456097 | -4.43536615 | -0.63531360 |
| H | -0.32454206 | -4.27426593 | 1.08804293 |
| C | -1.77427285 | -2.01514099 | 0.34728708 |
| H | 0.95731943 | -4.15807034 | -0.14696884 |
| C | -0.45957907 | 3.03345949 | -0.92008420 |
| C | 0.29269327 | 2.04141432 | -0.05419005 |
| O | -0.26375872 | 1.22528870 | 0.68692919 |
| O | 1.61947881 | 2.13549951 | -0.18195558 |
| H | -1.53454174 | 2.93843780 | -0.75680105 |
| H | -0.22699636 | 2.85051792 | -1.97589502 |
| H | -0.13431161 | 4.05345689 | -0.68608852 |
| H | 2.12173535 | 1.40844601 | 0.41122218 |

**2AcOH-TS2**
***E*** = -4872.63
***H*** = -4667.11
***G*** = -4715.60
***N*imag** = -67.4914

| | | | |
|---|---|---|---|
| H | 4.42418704 | -3.75878416 | -1.71222319 |
| H | 5.89536098 | -2.74851651 | -1.53083399 |
| H | 4.65193248 | -2.29215223 | -2.70286838 |
| H | 3.78492485 | 0.04297259 | 0.53635238 |
| C | 2.61777966 | -0.49685501 | 2.07525683 |
| O | 3.16032797 | 0.52296781 | 1.23611111 |
| H | 1.10166675 | 0.79035556 | 2.93699949 |
| H | 3.40742669 | -1.18153166 | 2.40700613 |
| C | 1.89759048 | 0.12465393 | 3.27926984 |
| H | 1.47416272 | -0.65908130 | 3.91511833 |
| H | 2.63489469 | 0.69169345 | 3.85526852 |
| C | 4.81559926 | -2.73894354 | -1.71410073 |
| C | 4.11681180 | -1.87512225 | -0.66519339 |
| O | 3.08786844 | -2.35573661 | -0.08827338 |
| O | 4.60669815 | -0.71136292 | -0.44781605 |
| H | -1.98183987 | -2.18477912 | 1.51996872 |
| H | -2.54184272 | -2.26680256 | -0.16596062 |
| H | -1.88685013 | -0.74065396 | 0.48093719 |
| C | -0.09319434 | -1.74317737 | -1.39837691 |
| H | 0.92530333 | -2.00826454 | -1.69285352 |

| | | | |
|---|---|---|---|
| H | -0.80370967 | -2.20647770 | -2.08972215 |
| H | -0.21361867 | -0.65750092 | -1.44937335 |
| C | 1.65170048 | -1.30027446 | 1.26966690 |
| N | 0.59188950 | -1.63175975 | 0.91416383 |
| C | -0.39137811 | -2.25769288 | 0.02705238 |
| C | -0.18935704 | -3.78430235 | 0.12752821 |
| H | -0.91728473 | -4.27440394 | -0.52656119 |
| H | -0.35195697 | -4.12984163 | 1.15312497 |
| C | -1.79282213 | -1.83024712 | 0.50175646 |
| H | 0.82120352 | -4.05522435 | -0.18980402 |
| C | -0.51649127 | 2.69118365 | -1.22130556 |
| C | 0.27456906 | 1.96446138 | -0.15680220 |
| O | -0.21436036 | 1.44173740 | 0.84080738 |
| O | 1.59743631 | 1.93649849 | -0.43083114 |
| H | -1.57444832 | 2.70713495 | -0.95552646 |
| H | -0.38177287 | 2.19141245 | -2.18781784 |
| H | -0.14120901 | 3.71496232 | -1.32904983 |
| H | 2.09660464 | 1.41716513 | 0.28194493 |

**2AcOH-INT3**
***E*** = -4880.54
***H*** = -4673.20
***G*** = -4720.04
***N*imag** = 0

| | | | |
|---|---|---|---|
| H | -16.00280553 | -11.61733044 | -5.06600470 |
| H | -6.78210662 | -12.60358386 | -6.18923798 |
| H | -12.66455684 | -10.28315559 | -5.16262708 |
| O | -13.16609492 | -12.33194040 | -6.21096663 |
| H | -7.52072668 | -12.69313101 | -7.82370945 |
| H | -7.86109603 | -13.94369691 | -6.58994952 |
| H | -10.72740449 | -10.25983103 | -6.21067716 |
| C | -13.88547224 | -11.76887407 | -5.39131285 |
| C | -10.24125936 | -10.08609745 | -4.30622275 |
| O | -11.09352356 | -9.74736476 | -5.44617607 |
| H | -8.34423348 | -9.57597163 | -5.26340044 |
| H | -10.81163666 | -9.77921054 | -3.42710952 |
| C | -8.91074340 | -9.33626463 | -4.35854292 |
| H | -9.10580490 | -8.25967227 | -4.34682143 |
| H | -8.29878415 | -9.59027086 | -3.48525064 |
| H | -12.10904065 | -13.52656799 | -1.50196391 |
| H | -12.15232409 | -15.15629860 | -2.22045837 |
| H | -13.00628668 | -13.81097078 | -3.00795287 |
| C | -10.89431529 | -14.52279256 | -4.60669323 |
| H | -9.94174905 | -14.48024464 | -5.13844842 |
| H | -11.12285313 | -15.57599719 | -4.40904890 |
| H | -11.67930601 | -14.11065376 | -5.24817265 |
| C | -10.11015382 | -11.60363069 | -4.19452246 |

| | | | |
|---|---|---|---|
| N | -10.85634319 | -12.28028754 | -3.44832460 |
| C | -10.83972002 | -13.75809323 | -3.26287218 |
| C | -9.58075615 | -14.14402456 | -2.45261974 |
| H | -9.60945008 | -15.21504160 | -2.22246324 |
| H | -9.54678034 | -13.58946241 | -1.50812198 |
| C | -12.10775667 | -14.08480641 | -2.44433335 |
| H | -8.66882469 | -13.93301738 | -3.01840443 |
| O | -13.58432882 | -10.56708042 | -4.85081644 |
| C | -15.19013966 | -12.32466043 | -4.86559754 |
| H | -15.40623886 | -13.28393990 | -5.33820220 |
| H | -15.12687571 | -12.44931145 | -3.77831540 |
| C | -7.67584271 | -12.87661708 | -6.76003242 |
| C | -8.85767466 | -12.08426637 | -6.27234009 |
| O | -9.00330781 | -12.21153507 | -4.91954641 |
| O | -9.60775716 | -11.42509542 | -6.98221410 |

**2AcOH-INT3***
***E*** = -4881.16
***H*** = -4674.57
***G*** = -4720.46
***N*imag** = 0

| | | | |
|---|---|---|---|
| H | -2.57999463 | -8.18566256 | 3.40163047 |
| H | 0.92564737 | -7.01088887 | 1.12671327 |
| H | 0.45352702 | -8.72428718 | 1.26063233 |
| H | -0.51654973 | -7.60249800 | 0.27706130 |
| C | -4.38210679 | -6.35420918 | 5.60236991 |
| C | -3.93929928 | -5.94869913 | 4.22361869 |
| O | -2.56382859 | -5.74598969 | 4.19976505 |
| O | -4.63509092 | -5.82438158 | 3.23590783 |
| H | -5.45668485 | -6.54017244 | 5.60324960 |
| H | -4.13565733 | -5.56030004 | 6.31616526 |
| H | -3.84251025 | -7.25537251 | 5.91434751 |
| H | -1.07184736 | -4.30812050 | 1.21349183 |
| C | -2.22329497 | -3.80341588 | 2.70269887 |
| O | -1.55332198 | -3.47524163 | 1.46056439 |
| H | -1.89927377 | -1.85372432 | 3.57540825 |
| H | -3.30055769 | -3.65776840 | 2.55532541 |
| C | -1.72930565 | -2.90113536 | 3.84268980 |
| H | -2.27740566 | -3.11542856 | 4.76519771 |
| H | -0.65954511 | -3.05564415 | 4.02055958 |
| C | 0.06195144 | -7.49255852 | 3.71375292 |
| H | -0.53356850 | -7.27505252 | 4.60500789 |
| H | 0.91243365 | -6.80267962 | 3.68084445 |
| H | 0.44970647 | -8.51418063 | 3.79369511 |
| C | -1.97162046 | -5.27741238 | 2.99237670 |
| N | -1.20022661 | -5.94303705 | 2.25966034 |
| C | -0.78686687 | -7.36264926 | 2.42852501 |

| | | | |
|---|---|---|---|
| C | -2.01193296 | -8.30330953 | 2.47590184 |
| H | -1.67082308 | -9.34277632 | 2.41936739 |
| H | -2.67706130 | -8.11275048 | 1.62692172 |
| C | 0.07557077 | -7.69815120 | 1.19418375 |
| C | 2.73896265 | -1.53383198 | 0.52746362 |
| C | 1.49094569 | -2.37697193 | 0.65673894 |
| O | 1.35784206 | -3.50981458 | 0.20132461 |
| O | 0.50926736 | -1.74445920 | 1.34251983 |
| H | 2.49769760 | -0.59296627 | 0.01968653 |
| H | 3.11916195 | -1.28169252 | 1.52407707 |
| H | 3.50038928 | -2.07596281 | -0.03526155 |
| H | -0.28323390 | -2.36719418 | 1.41034179 |

**2HFIP-INT1**

***E*** = -3806.98

***H*** = -3683.20

***G*** = -3734.74

***N*imag** = 0

| | | | |
|---|---|---|---|
| F | 0.55506370 | 3.96558406 | 0.37120922 |
| F | -0.73912487 | 3.66823002 | -1.38323658 |
| F | 1.17296152 | 4.74123184 | -1.59385516 |
| C | 1.28895285 | 2.35402481 | -1.21740880 |
| C | 3.00889932 | 0.33051994 | 1.83466269 |
| O | 2.58185832 | 0.00082216 | 0.72043711 |
| H | 1.61217929 | 1.93619526 | 2.16633225 |
| H | 3.86115354 | -0.22835766 | 2.25871032 |
| C | 2.45742868 | 1.44176216 | 2.64979875 |
| H | 2.16128377 | 1.04188239 | 3.62994699 |
| H | 3.26592018 | 2.15954255 | 2.85081151 |
| O | 0.57159309 | 1.33251255 | -0.56461987 |
| C | 1.45403776 | 2.00104165 | -2.72129410 |
| F | 2.17576639 | 2.93686456 | -3.39989434 |
| F | 2.13138410 | 0.81311341 | -2.82264045 |
| H | 2.30403799 | 2.47875014 | -0.82047745 |
| H | 1.22436395 | 0.78748168 | -0.05329831 |
| C | 0.55779184 | 3.69733079 | -0.96890689 |
| F | 3.17918733 | -2.26139446 | -3.35604198 |
| F | 4.93644067 | -3.44280375 | -2.76173498 |
| F | 5.19440263 | -1.60163670 | -3.94458836 |
| C | 4.53819495 | -1.33548460 | -1.63542094 |
| O | 3.81172255 | -2.02083219 | -0.64245979 |
| C | 5.98725572 | -1.05767397 | -1.14417133 |
| F | 6.70304478 | -0.31337472 | -2.03480213 |
| F | 5.92659453 | -0.34631578 | 0.02480014 |
| F | 6.68800686 | -2.19685594 | -0.89093775 |
| H | 4.10509649 | -0.35691418 | -1.87921643 |
| C | 4.47436606 | -2.17349823 | -2.93635524 |
| H | 3.32544571 | -1.34402679 | -0.10201693 |

| | | | |
|---|---|---|---|
| F | 0.26316674 | 1.84495489 | -3.35871960 |

**2HFIP-TS1**
***E*** = -5704.09
***H*** = -5494.56
***G*** = -5558.19
***N*imag** = -363.4090

| | | | |
|---|---|---|---|
| F | 1.62681590 | 3.59615320 | 0.37935840 |
| F | -0.12626245 | 3.43098180 | -0.93847072 |
| F | 1.64910444 | 4.55632403 | -1.60165451 |
| C | 1.86143494 | 2.13823333 | -1.48819908 |
| C | 2.63263932 | -0.14493767 | 2.21705893 |
| O | 3.16190122 | 0.40418943 | 1.16357216 |
| H | 0.98525928 | 1.24204024 | 2.48635157 |
| H | 3.26131463 | -0.85054316 | 2.77591154 |
| C | 1.72004956 | 0.70203254 | 3.09072313 |
| H | 1.21176868 | 0.09250049 | 3.84371322 |
| H | 2.35805419 | 1.43588988 | 3.59946434 |
| O | 1.47345374 | 1.03590990 | -0.71407712 |
| C | 1.44969898 | 1.84676533 | -2.95202741 |
| F | 1.76084309 | 2.87677470 | -3.78987964 |
| F | 2.11794224 | 0.74060908 | -3.39968706 |
| H | -0.10988469 | -4.29855781 | 0.20017831 |
| H | -1.61948130 | -3.85811702 | -0.63099757 |
| H | -1.40911685 | -3.46269077 | 1.09083520 |
| C | -1.39136640 | -1.07962554 | -0.35102351 |
| H | -0.94140597 | -0.11637407 | -0.60536262 |
| H | -2.11914705 | -1.35032928 | -1.12199239 |
| H | -1.91032638 | -0.99680866 | 0.60874350 |
| C | 1.39448162 | -1.40937803 | 1.54692918 |
| N | 0.64933768 | -1.77488541 | 0.73415005 |
| C | -0.30935479 | -2.17960073 | -0.29359189 |
| C | 0.45993838 | -2.27976557 | -1.62722337 |
| H | -0.24600814 | -2.56173281 | -2.41395775 |
| H | 1.24007611 | -3.04309663 | -1.56976738 |
| C | -0.89689076 | -3.54187031 | 0.12725939 |
| H | 0.90534124 | -1.31537750 | -1.88356337 |
| F | 0.11712676 | 1.59276829 | -3.08110364 |
| H | 2.94821490 | 2.29966122 | -1.49940290 |
| H | 2.13126460 | 0.90618798 | 0.04644793 |
| C | 1.23722763 | 3.44573785 | -0.92241850 |
| F | 3.71607800 | -3.26444689 | -0.32321778 |
| F | 5.69724100 | -3.32802661 | -1.27984126 |
| F | 3.87695417 | -3.22365263 | -2.51743810 |
| C | 4.48418781 | -1.22435039 | -1.27839015 |
| O | 5.07151388 | -0.83668998 | -0.06722633 |
| C | 5.24564187 | -0.55391814 | -2.45297289 |

| | | | |
|---|---|---|---|
| F | 4.73241498 | -0.89272646 | -3.67152501 |
| F | 5.14262255 | 0.80575677 | -2.33225653 |
| F | 6.57230669 | -0.86219012 | -2.46133584 |
| H | 3.43863145 | -0.89572378 | -1.36806249 |
| C | 4.45689153 | -2.77373603 | -1.36444484 |
| H | 4.36183657 | -0.34749898 | 0.47729726 |

**2HFIP-INT2**

***E*** = -5709.38

***H*** = -5500.77

***G*** = -5560.36

***N*imag** = 0

| | | | |
|---|---|---|---|
| F | -0.03155149 | 2.18456243 | 0.10886501 |
| F | -0.52072586 | 1.86168780 | -2.01076747 |
| F | -0.01823315 | 3.88269279 | -1.29118146 |
| C | 1.80270887 | 2.26893866 | -1.41025074 |
| C | 2.56170869 | -0.11442299 | 1.94434207 |
| O | 3.32566908 | 0.42956215 | 0.91854881 |
| H | 1.36158582 | 1.65894410 | 2.35364135 |
| H | 3.12040727 | -0.86326981 | 2.53390847 |
| C | 1.96475670 | 0.93487977 | 2.90877023 |
| H | 1.35155779 | 0.46137493 | 3.68351633 |
| H | 2.80294451 | 1.45408884 | 3.38253942 |
| O | 2.05432971 | 0.90996492 | -1.21468678 |
| C | 2.28273713 | 2.66941909 | -2.82894833 |
| F | 2.05115635 | 3.98855170 | -3.10669268 |
| F | 3.62792160 | 2.46276834 | -2.92744398 |
| H | 0.35728039 | -4.01900121 | 0.18831158 |
| H | -1.35366601 | -3.94412817 | -0.28988833 |
| H | -0.88176468 | -3.52211524 | 1.37264146 |
| C | -1.71665858 | -1.19189379 | 0.10785844 |
| H | -1.58075145 | -0.15075684 | -0.19590406 |
| H | -2.51881208 | -1.63221774 | -0.49140935 |
| H | -2.00504135 | -1.22733827 | 1.16241740 |
| C | 1.43750003 | -0.88656005 | 1.31734744 |
| N | 0.61820319 | -1.39955416 | 0.68857718 |
| C | -0.43051671 | -2.00797299 | -0.13783148 |
| C | 0.03678137 | -1.91031359 | -1.60533079 |
| H | -0.76640190 | -2.29531243 | -2.24038604 |
| H | 0.93185685 | -2.51512653 | -1.76799501 |
| C | -0.58001789 | -3.47172294 | 0.32243438 |
| H | 0.24968158 | -0.87429980 | -1.87787521 |
| F | 1.68534604 | 1.93300643 | -3.81083641 |
| H | 2.35053702 | 2.91326211 | -0.70813532 |
| H | 2.56674255 | 0.78008274 | -0.31590846 |
| C | 0.29561859 | 2.56026464 | -1.16883699 |
| F | 3.18252563 | -3.05199924 | -0.42942731 |

| | | | |
|---|---|---|---|
| F | 5.13626461 | -3.76145438 | -1.14957810 |
| F | 3.55651282 | -3.22885895 | -2.59069627 |
| C | 4.60653077 | -1.39539169 | -1.38241537 |
| O | 5.10773955 | -1.07362927 | -0.11688560 |
| C | 5.68612848 | -1.08733002 | -2.45076543 |
| F | 5.26416948 | -1.39021018 | -3.71695320 |
| F | 5.97817912 | 0.24576124 | -2.43016809 |
| F | 6.85190818 | -1.76289298 | -2.23426345 |
| H | 3.72916886 | -0.79118978 | -1.65955544 |
| C | 4.13439973 | -2.87491770 | -1.40273172 |
| H | 4.40394760 | -0.47406687 | 0.35691185 |

**2HFIP-INT2**
***E*** = -5701.13
***H*** = -5492.52
***G*** = -5550.61
***N*imag** = -91.6081

| | | | |
|---|---|---|---|
| F | -0.33782443 | 2.46124361 | -0.11618587 |
| F | -0.41360527 | 1.97372425 | -2.25908188 |
| F | -0.24164692 | 4.07220389 | -1.61278722 |
| C | 1.70948976 | 2.66430432 | -1.31519250 |
| C | 2.81871979 | -0.03412549 | 1.80467171 |
| O | 3.69033739 | 0.70363755 | 0.93366409 |
| H | 1.21466519 | 1.43656379 | 1.78885172 |
| H | 3.40408316 | -0.60859013 | 2.53365864 |
| C | 1.82852480 | 0.89491483 | 2.51334732 |
| H | 1.17866245 | 0.31978763 | 3.17910761 |
| H | 2.40211322 | 1.60980647 | 3.11019372 |
| O | 2.01912031 | 1.31233645 | -1.09271893 |
| C | 2.39907210 | 3.15405825 | -2.61900202 |
| F | 2.16112233 | 4.47694168 | -2.86507295 |
| F | 3.74897486 | 2.99889714 | -2.49615820 |
| H | 0.59760913 | -4.13022504 | 0.02835663 |
| H | -1.16045233 | -3.90022601 | -0.11175656 |
| H | -0.34042839 | -3.53563944 | 1.42349049 |
| C | -1.20756616 | -1.13930904 | 0.31815793 |
| H | -1.04387284 | -0.10924804 | -0.00896013 |
| H | -2.14254026 | -1.50352340 | -0.11793960 |
| H | -1.29672349 | -1.16185528 | 1.40829461 |
| C | 2.07779567 | -1.02365309 | 0.96085322 |
| N | 1.16691961 | -1.53355904 | 0.45543476 |
| C | -0.05666936 | -2.05416799 | -0.15623597 |
| C | 0.11808796 | -1.98380191 | -1.68742607 |
| H | -0.82273460 | -2.28897987 | -2.15468008 |
| H | 0.90633421 | -2.66282522 | -2.02209907 |
| C | -0.24348887 | -3.50378714 | 0.33421112 |
| H | 0.35569160 | -0.96408779 | -2.00310881 |

| | | | |
|---|---|---|---|
| F | 2.00301540 | 2.45042077 | -3.71730510 |
| H | 2.06315985 | 3.32544919 | -0.51443853 |
| H | 2.71058358 | 1.23732513 | -0.37444361 |
| C | 0.16628915 | 2.80016140 | -1.34364330 |
| F | 3.04561893 | -4.06128612 | -0.65192210 |
| F | 5.22228349 | -4.03682125 | -0.95873989 |
| F | 3.86242918 | -3.94175810 | -2.69091662 |
| C | 3.98827019 | -1.95086888 | -1.26715166 |
| O | 4.13743429 | -1.58227941 | 0.04013069 |
| C | 5.04382945 | -1.25034892 | -2.17888388 |
| F | 4.86157697 | -1.51112337 | -3.51341343 |
| F | 4.94594607 | 0.10587492 | -2.02509855 |
| F | 6.33058632 | -1.59522298 | -1.87172312 |
| H | 3.01254059 | -1.67038024 | -1.71574043 |
| C | 4.04877466 | -3.50265088 | -1.40405014 |
| H | 4.13825604 | -0.07544214 | 0.42033459 |

**2HFIP-INT3**

***E*** = -5714.11

***H*** = -5502.96

***G*** = -5562.34

***N*imag** = 0

| | | | |
|---|---|---|---|
| F | -0.49576895 | 3.28528113 | 0.13506071 |
| F | -0.97926431 | 3.79352004 | -1.94964015 |
| F | -0.09551610 | 5.31248753 | -0.62182413 |
| C | 1.35266727 | 3.50865442 | -1.35003033 |
| C | 2.70832607 | 0.17675997 | 1.06956760 |
| O | 3.06426400 | 0.96483930 | -0.11787302 |
| H | 0.82557424 | 1.23857476 | 1.37721721 |
| H | 3.62674593 | 0.01459505 | 1.64429012 |
| C | 1.75119516 | 1.00498102 | 1.90461481 |
| H | 1.51279800 | 0.47800805 | 2.83155688 |
| H | 2.24932769 | 1.94370556 | 2.16629722 |
| O | 1.27615457 | 2.15796633 | -1.73566095 |
| C | 1.99244475 | 4.33871845 | -2.49602296 |
| F | 2.13884516 | 5.65481486 | -2.16444294 |
| F | 3.23747356 | 3.84555484 | -2.76538494 |
| H | -0.56315990 | -3.13816817 | -1.08105932 |
| H | -1.96612597 | -2.87917539 | -0.01683437 |
| H | -0.56461031 | -3.81088076 | 0.56390449 |
| C | -0.65849348 | -1.45294829 | 1.95566108 |
| H | -0.33039460 | -0.49312020 | 2.35488203 |
| H | -1.75150323 | -1.49082008 | 2.01802637 |
| H | -0.25141047 | -2.25331586 | 2.58328807 |
| C | 2.26668063 | -1.21392668 | 0.58419109 |
| N | 1.23066854 | -1.87019657 | 0.37641009 |
| C | -0.22730253 | -1.65362393 | 0.48625248 |

| | | | |
|---|---|---|---|
| C | -0.66523732 | -0.47562605 | -0.41015305 |
| H | -1.75828198 | -0.40263206 | -0.40854327 |
| H | -0.33276609 | -0.63783787 | -1.44085760 |
| C | -0.87371531 | -2.95531457 | -0.04656055 |
| H | -0.26031493 | 0.47833092 | -0.06995291 |
| F | 1.27053126 | 4.27911163 | -3.65125467 |
| H | 1.98722340 | 3.66257507 | -0.46881500 |
| H | 1.91247267 | 1.64069612 | -1.17126546 |
| C | -0.06810755 | 3.99008704 | -0.95642678 |
| F | 3.61445898 | -4.21173866 | 1.66253653 |
| F | 5.52796260 | -3.79248526 | 0.66339959 |
| F | 4.19706766 | -5.35996743 | -0.12533870 |
| C | 3.47730742 | -3.08328880 | -0.42077670 |
| O | 3.53867082 | -1.82973310 | 0.23661446 |
| C | 4.03386284 | -2.91066879 | -1.85043867 |
| F | 3.94584031 | -4.06415678 | -2.57037870 |
| F | 3.30261673 | -1.95761294 | -2.50167438 |
| F | 5.33640287 | -2.50557504 | -1.87071624 |
| H | 2.43044919 | -3.40765362 | -0.50347219 |
| C | 4.22243018 | -4.12074727 | 0.44414406 |
| H | 3.79332619 | 0.50807155 | -0.57833158 |

**2HFIP-INT3***
***E*** = -5719.22
***H*** = -5508.05
***G*** = -5566.14
***N*imag** = 0

| | | | |
|---|---|---|---|
| H | 2.85024251 | -0.85878158 | -0.30110315 |
| H | 3.34425808 | -0.67660351 | -2.00094339 |
| H | -0.55223614 | 1.90217422 | 0.77250070 |
| H | 3.51933006 | -2.24538536 | -1.18706869 |
| C | 2.88539143 | -1.35596880 | -1.27399436 |
| H | 2.18314209 | -3.42048378 | -2.98829667 |
| H | 0.57900073 | -2.80292492 | -3.44902150 |
| H | -0.40442048 | 0.33410109 | -3.24174775 |
| C | -0.50593931 | 1.51708912 | -1.72073095 |
| O | -0.91125302 | 1.17565770 | -3.06642176 |
| H | -0.25180466 | 3.61501224 | -2.17068471 |
| H | -1.42343408 | 1.65473493 | -1.13616067 |
| C | 0.32961218 | 2.80759818 | -1.71668589 |
| H | 1.24906129 | 2.66077081 | -2.29055443 |
| H | 0.60120237 | 3.11344901 | -0.70218692 |
| C | 0.78583163 | -2.68401052 | -0.70266866 |
| H | -0.23905804 | -2.91465659 | -1.01408731 |
| H | 1.33985462 | -3.62578659 | -0.62162800 |
| H | 0.75729287 | -2.21557039 | 0.28346788 |
| C | 0.29285035 | 0.32023121 | -1.18688554 |

| | | | |
|---|---|---|---|
| N | 0.65153639 | -0.56402190 | -2.00961726 |
| C | 1.47391300 | -1.77425969 | -1.74580559 |
| C | 1.57540033 | -2.51530983 | -3.09513505 |
| H | 2.03559526 | -1.86949872 | -3.85086203 |
| C | 1.01377369 | 1.51133499 | 2.17469314 |
| F | 1.76710809 | 0.49830426 | 2.67546340 |
| F | 0.42264406 | 2.15702908 | 3.21529267 |
| F | 1.85540151 | 2.38940219 | 1.55850921 |
| C | -0.04077003 | 1.01585956 | 1.15342779 |
| O | 0.68113732 | 0.29761247 | 0.15640737 |
| C | -1.13172584 | 0.09068933 | 1.76052875 |
| F | -1.90583876 | 0.76018895 | 2.65500723 |
| F | -1.94181653 | -0.35186456 | 0.75375733 |
| F | -0.61002978 | -1.00003467 | 2.37949176 |
| C | -0.19116199 | 1.10361711 | -6.76340488 |
| F | -1.44981021 | 1.29262216 | -7.25306626 |
| F | -0.25777752 | 0.07844684 | -5.85926649 |
| F | 0.59343444 | 0.68784039 | -7.79861053 |
| C | 0.31980679 | 2.42566561 | -6.12391164 |
| O | -0.55346788 | 2.88435083 | -5.12646261 |
| C | 1.78325219 | 2.32881452 | -5.60527977 |
| F | 2.66438655 | 2.01078148 | -6.59762375 |
| F | 2.16342877 | 3.53496513 | -5.09255221 |
| F | 1.92791792 | 1.40202786 | -4.61154893 |
| H | 0.33599207 | 3.16528248 | -6.93160513 |
| H | -0.62369863 | 2.22933336 | -4.36998084 |

**AcOH-HFIP-INT1**

***E*** = -3390.39

***H*** = -3267.81

***G*** = -3314.48

***N*imag** = 0

| | | | |
|---|---|---|---|
| F | 2.01057900 | 3.62014577 | 0.29357991 |
| F | 0.32333785 | 4.33422933 | -0.92445508 |
| F | 2.37974892 | 4.46203775 | -1.70604270 |
| C | 1.39803937 | 2.24695408 | -1.55187073 |
| C | 2.47176372 | -0.01200007 | 1.83983692 |
| O | 2.21887118 | -0.15029074 | 0.63493618 |
| H | 1.18524933 | 1.68681238 | 2.16633923 |
| H | 3.18712008 | -0.71442876 | 2.29988338 |
| C | 1.87720567 | 1.03765188 | 2.70651468 |
| H | 1.36972942 | 0.54564469 | 3.54852482 |
| H | 2.69375335 | 1.62564089 | 3.14969030 |
| O | 0.53471692 | 1.48810893 | -0.73782891 |
| C | 0.86547638 | 2.19848752 | -3.00658924 |
| F | 1.62983846 | 2.93989303 | -3.86002213 |
| F | 0.88728062 | 0.90846539 | -3.45297486 |

| | | | |
|---|---|---|---|
| H | 2.41720653 | 1.84244327 | -1.58232976 |
| H | 1.09259547 | 0.87051765 | -0.19337476 |
| C | 1.52124489 | 3.68728310 | -0.98223479 |
| C | 5.07943181 | -3.95637378 | -0.60890844 |
| C | 4.35225792 | -2.84804180 | 0.11392942 |
| O | 4.48921247 | -2.57407369 | 1.30338520 |
| O | 3.51002666 | -2.16781575 | -0.70097023 |
| H | 5.75198556 | -4.47237908 | 0.07762747 |
| H | 5.64743914 | -3.53939714 | -1.44825887 |
| H | 4.35238213 | -4.66408393 | -1.02335699 |
| F | -0.41900440 | 2.64381305 | -3.10613849 |
| H | 3.05409202 | -1.44756143 | -0.17209339 |

**AcOH-HFIP-TS1**
***E*** = -5285.87
***H*** = -5078.84
***G*** = -5135.67
***N*imag** = -365.9188

| | | | |
|---|---|---|---|
| F | 2.05269718 | 4.09307014 | -0.30476375 |
| F | 0.24195904 | 4.03163642 | -1.55287675 |
| F | 2.22721778 | 4.20168700 | -2.49513164 |
| C | 1.66403683 | 2.06219204 | -1.48832279 |
| C | 2.40816464 | -0.08749527 | 2.26066598 |
| O | 2.78525416 | 0.38171668 | 1.10916839 |
| H | 0.95282013 | 1.44017688 | 2.76511965 |
| H | 3.05983727 | -0.84466532 | 2.71641216 |
| C | 1.74062549 | 0.85341208 | 3.24710561 |
| H | 1.32703608 | 0.30414547 | 4.09812294 |
| H | 2.51334336 | 1.54274551 | 3.61069948 |
| O | 0.97750707 | 1.52490023 | -0.39051386 |
| C | 1.11796771 | 1.41401459 | -2.78742922 |
| F | 1.70282857 | 1.91985682 | -3.91293182 |
| F | 1.37529868 | 0.07175861 | -2.76508800 |
| H | -0.51296053 | -4.40003986 | 1.08421383 |
| H | -1.77942298 | -4.17098417 | -0.14409117 |
| H | -1.87823654 | -3.29831753 | 1.40300572 |
| C | -1.35164282 | -1.44576165 | -0.60661521 |
| H | -0.78009549 | -0.59752763 | -0.99201818 |
| H | -1.91596605 | -1.90051532 | -1.42639526 |
| H | -2.05664823 | -1.08669279 | 0.14932240 |
| C | 1.02485045 | -1.31292510 | 1.82946712 |
| N | 0.36309354 | -1.85687433 | 1.04461408 |
| C | -0.40856822 | -2.51099622 | -0.00783281 |
| C | 0.60465814 | -3.02455156 | -1.05396176 |
| H | 0.05255379 | -3.51150351 | -1.86333503 |
| H | 1.28743767 | -3.75060592 | -0.60402324 |
| C | -1.19471680 | -3.67177206 | 0.63451675 |

| | | | |
|---|---|---|---|
| H | 1.18316108 | -2.19583821 | -1.47028689 |
| F | -0.23142980 | 1.56531859 | -2.91997074 |
| H | 2.73993922 | 1.84428104 | -1.46711714 |
| H | 1.66442182 | 1.13279722 | 0.24401021 |
| C | 1.53791301 | 3.61032149 | -1.47409580 |
| C | 4.59301724 | -3.40993784 | -1.23794844 |
| C | 4.05179541 | -2.48672109 | -0.16810342 |
| O | 3.69981063 | -2.85649172 | 0.95183089 |
| O | 3.97382057 | -1.20846775 | -0.57919542 |
| H | 4.73280087 | -4.41385591 | -0.83394284 |
| H | 5.54109775 | -3.02320904 | -1.62667738 |
| H | 3.88631588 | -3.44562517 | -2.07606415 |
| H | 3.54548390 | -0.62587914 | 0.15383595 |

**AcOH-HFIP-INT2**

***E*** = -5290.12

***H*** = -5082.68

***G*** = -5139.83

***N*imag** = 0

| | | | |
|---|---|---|---|
| F | -1.84490512 | -1.09121374 | -4.22519963 |
| H | -4.50272495 | -0.63078032 | -2.29629762 |
| H | -1.66527534 | -0.06861380 | -1.74995800 |
| C | -3.24338744 | -2.24086164 | -1.70574472 |
| F | -3.29688183 | -2.09246189 | -0.34848766 |
| F | -2.04349034 | -2.83259829 | -1.99426868 |
| F | -4.22456402 | -3.12791706 | -2.05255645 |
| C | -3.43450347 | -0.85969284 | -2.39960221 |
| C | 0.10027478 | -0.31123855 | -0.16531727 |
| O | -0.20203069 | -0.23429197 | -1.51823854 |
| H | 2.24451645 | 0.00496741 | -0.30818939 |
| H | -0.72990792 | 0.07506861 | 0.45618213 |
| C | 1.39856433 | 0.41113645 | 0.25051709 |
| H | 1.27199887 | 1.47154605 | 0.01208280 |
| H | 1.58277985 | 0.30720858 | 1.32625892 |
| H | -1.38533451 | -4.25040418 | 2.22217053 |
| H | -1.18406400 | -5.83453020 | 1.43889024 |
| H | -2.02001638 | -4.52247731 | 0.57870031 |
| C | 0.41147702 | -4.98671053 | -0.69988628 |
| H | 1.35622014 | -4.63319384 | -1.11965815 |
| H | 0.46342246 | -6.06986845 | -0.55481255 |
| H | -0.40083281 | -4.76323354 | -1.39609847 |
| C | 0.17010132 | -1.75723140 | 0.22603657 |
| N | 0.10703199 | -2.88509163 | 0.45765179 |
| C | 0.15455214 | -4.33183761 | 0.67400978 |
| C | 1.31722536 | -4.59747137 | 1.65321108 |
| H | 1.37808586 | -5.67549923 | 1.82943289 |
| H | 1.14446799 | -4.09351458 | 2.60869494 |

| | | | |
|---|---|---|---|
| C | -1.20386535 | -4.75421201 | 1.26819840 |
| H | 2.26444600 | -4.25391537 | 1.22793371 |
| O | -2.68997205 | 0.15430931 | -1.80358599 |
| C | -3.16579215 | -0.93097037 | -3.93165877 |
| F | -3.85058412 | -1.94915546 | -4.54059745 |
| F | -3.57222446 | 0.23598028 | -4.51705628 |
| C | 3.08813502 | -2.85323113 | -3.72787079 |
| C | 2.24954000 | -2.14353223 | -2.68213283 |
| O | 2.44002984 | -2.26565774 | -1.46581148 |
| O | 1.27841444 | -1.39667276 | -3.20478163 |
| H | 3.93794734 | -3.35142390 | -3.25768733 |
| H | 3.43780308 | -2.13989488 | -4.48182277 |
| H | 2.46768975 | -3.59688283 | -4.24334714 |
| H | 0.69130303 | -0.93137041 | -2.44369621 |

**AcOH-HFIP-TS2-AcOH**

***E*** = -5289.09
***H*** = -5084.65
***G*** = -5137.88
***N*imag** = -875.5279

| | | | |
|---|---|---|---|
| F | -1.84027871 | -0.52160125 | -4.25590115 |
| H | -4.47681595 | -1.10907871 | -2.33102188 |
| H | -1.77277909 | -0.26393821 | -1.62472706 |
| C | -2.95304799 | -2.59685572 | -2.27352396 |
| F | -3.13427506 | -2.94398592 | -0.96220857 |
| F | -1.63636918 | -2.80878590 | -2.56475270 |
| F | -3.68681394 | -3.46988999 | -3.02514062 |
| C | -3.39149958 | -1.12027464 | -2.48506714 |
| C | 0.22168855 | -0.32598826 | -0.15634334 |
| O | -0.20712533 | -0.14504652 | -1.47834764 |
| H | 2.35528481 | -0.07211716 | -0.47017617 |
| H | -0.54428076 | 0.06812541 | 0.53323399 |
| C | 1.57226449 | 0.33506129 | 0.17220490 |
| H | 1.46513749 | 1.40903432 | -0.00673860 |
| H | 1.84173333 | 0.17478406 | 1.22221129 |
| H | -1.15962338 | -4.07314299 | 2.62711829 |
| H | -1.14357804 | -5.70178940 | 1.91294335 |
| H | -2.07008996 | -4.40243044 | 1.12969281 |
| C | 0.09917189 | -5.03500215 | -0.50309904 |
| H | 0.95537454 | -4.71926742 | -1.10418619 |
| H | 0.16951080 | -6.10814070 | -0.30124740 |
| H | -0.82182858 | -4.84067918 | -1.05768978 |
| C | 0.24889282 | -1.78233300 | 0.18239548 |
| N | 0.05844106 | -2.85917476 | 0.55596544 |
| C | 0.09253381 | -4.29395291 | 0.85066722 |
| C | 1.39653940 | -4.54614030 | 1.63767244 |
| H | 1.45356019 | -5.61281746 | 1.87439777 |

| | | | |
|---|---|---|---|
| H | 1.40371795 | -3.97755651 | 2.57238303 |
| C | -1.15699034 | -4.63161073 | 1.68620002 |
| H | 2.26595142 | -4.26732997 | 1.03582314 |
| O | -2.79684206 | -0.25930172 | -1.55881981 |
| C | -3.16261271 | -0.62543038 | -3.94214263 |
| F | -3.74651930 | -1.44078401 | -4.87173739 |
| F | -3.71417616 | 0.61639152 | -4.08860401 |
| C | 2.89252517 | -2.77037119 | -3.76134884 |
| C | 2.04220770 | -2.06870828 | -2.71008876 |
| O | 2.09921660 | -2.40598186 | -1.50611757 |
| O | 1.26392520 | -1.12478558 | -3.17048459 |
| H | 3.61085295 | -3.44483959 | -3.29030028 |
| H | 3.41706546 | -2.03253054 | -4.37816650 |
| H | 2.23597001 | -3.34621467 | -4.42562038 |
| H | 0.54965057 | -0.65295499 | -2.29370026 |

**AcOH-HFIP-TS2-HFIP**

***E*** = -5283.44

***H*** = -5075.68

***G*** = -5131.76

***N*imag** = -91.8996

| | | | |
|---|---|---|---|
| F | -3.21887359 | -0.00605176 | -3.66304186 |
| H | -3.06247753 | -2.00703874 | -1.01406102 |
| H | -0.50141780 | -0.59627568 | -2.42996337 |
| C | -3.44481498 | -2.94660631 | -2.88309136 |
| F | -2.89749983 | -4.14849484 | -2.51419658 |
| F | -3.26032026 | -2.82070013 | -4.23044515 |
| F | -4.79648276 | -3.05314747 | -2.66688718 |
| C | -2.77243941 | -1.79925575 | -2.06557337 |
| C | 0.17735030 | -0.17052000 | -0.68157327 |
| O | 0.29145377 | -0.01673839 | -2.09706060 |
| H | 2.30141086 | -0.36754259 | -0.27922106 |
| H | -0.67332081 | 0.41893618 | -0.30879647 |
| C | 1.46302094 | 0.25679101 | 0.03614375 |
| H | 1.65837683 | 1.30201056 | -0.22118196 |
| H | 1.33422797 | 0.17791014 | 1.11965583 |
| H | -2.03330547 | -3.50353220 | 1.94730365 |
| H | -1.89420322 | -5.21002883 | 1.46783441 |
| H | -2.47434506 | -4.01014960 | 0.29410902 |
| C | 0.02274590 | -4.83543568 | -0.56460717 |
| H | 1.04997882 | -4.64527922 | -0.88568215 |
| H | -0.08322488 | -5.88828938 | -0.28507410 |
| H | -0.65645505 | -4.61022666 | -1.39001096 |
| C | -0.16220105 | -1.58921058 | -0.35426940 |
| N | -0.18087043 | -2.57761238 | 0.25450048 |
| C | -0.32199455 | -3.97152443 | 0.66863054 |
| C | 0.66999282 | -4.21176041 | 1.82482685 |

| | | | |
|---|---|---|---|
| H | 0.59466111 | -5.25688095 | 2.13975913 |
| H | 0.43554069 | -3.56953569 | 2.67938288 |
| C | -1.78168356 | -4.17775237 | 1.12314664 |
| H | 1.69537198 | -4.01546692 | 1.49794050 |
| O | -1.42530914 | -1.79330297 | -2.29206858 |
| C | -3.42836067 | -0.41710556 | -2.37729697 |
| F | -4.78031453 | -0.38211970 | -2.15435594 |
| F | -2.87426774 | 0.53883684 | -1.55959114 |
| C | 3.85036202 | -3.09045383 | -3.44105390 |
| C | 2.81964220 | -2.30857497 | -2.65948233 |
| O | 2.46896565 | -2.56738323 | -1.51150191 |
| O | 2.30557019 | -1.27817078 | -3.36940544 |
| H | 4.30910138 | -3.84646145 | -2.80200090 |
| H | 4.61557585 | -2.41421186 | -3.83719726 |
| H | 3.36514526 | -3.57523956 | -4.29670927 |
| H | 1.59135789 | -0.81384015 | -2.82222610 |

**AcOH-HFIP-INT3**
***E*** = -5298.58
***H*** = -5090.02
***G*** = -5139.92
***N*imag** = 0

| | | | |
|---|---|---|---|
| F | -13.02315270 | -10.00178943 | -8.03989696 |
| H | -15.50774294 | -10.94250179 | -6.04821282 |
| H | -12.78204767 | -10.19683795 | -5.42601551 |
| C | -13.98830691 | -12.39626574 | -6.41082672 |
| F | -14.15091277 | -13.02341101 | -5.21098460 |
| F | -12.67093160 | -12.51485617 | -6.75612879 |
| F | -14.71930773 | -13.08797330 | -7.33378316 |
| C | -14.43936278 | -10.91378172 | -6.28829244 |
| C | -10.35085607 | -10.22062018 | -4.35259865 |
| O | -11.13012376 | -9.91501948 | -5.54943670 |
| H | -8.42542737 | -9.57481152 | -5.15802827 |
| H | -11.00424417 | -9.96775252 | -3.51511023 |
| C | -9.07153829 | -9.38510995 | -4.29537660 |
| H | -9.33777369 | -8.32389629 | -4.28799863 |
| H | -8.51022507 | -9.61205118 | -3.38163039 |
| H | -11.92249406 | -13.79822066 | -1.52027979 |
| H | -11.86311817 | -15.42354317 | -2.24501588 |
| H | -12.83337190 | -14.14046583 | -3.00648153 |
| C | -10.69834337 | -14.70749165 | -4.64766469 |
| H | -9.78707190 | -14.54406568 | -5.22635046 |
| H | -10.77686128 | -15.77957135 | -4.43633520 |
| H | -11.56032593 | -14.41403984 | -5.25390232 |
| C | -10.11621443 | -11.72521563 | -4.24231380 |
| N | -10.82851066 | -12.46029400 | -3.51877931 |
| C | -10.68371776 | -13.92920186 | -3.31153058 |

| | | | |
|---|---|---|---|
| C | -9.38325959 | -14.20569672 | -2.52161379 |
| H | -9.33290259 | -15.27016264 | -2.26648991 |
| H | -9.37180450 | -13.62845463 | -1.59030589 |
| C | -11.90456092 | -14.35118308 | -2.46581289 |
| H | -8.49849591 | -13.94525599 | -3.10856439 |
| O | -13.77009344 | -10.25604924 | -5.24720879 |
| C | -14.31963830 | -10.12671457 | -7.62414693 |
| F | -15.02460686 | -10.70629947 | -8.63863275 |
| F | -14.81258753 | -8.86571970 | -7.45299580 |
| C | -7.47496947 | -12.78854588 | -6.70214035 |
| C | -8.74212619 | -12.10463263 | -6.26833373 |
| O | -8.93435607 | -12.24294777 | -4.92374190 |
| O | -9.51571748 | -11.51344649 | -7.01338286 |
| H | -6.62741652 | -12.38923524 | -6.13440319 |
| H | -7.32016996 | -12.63748245 | -7.77086635 |
| H | -7.54271124 | -13.85965477 | -6.48029118 |
| H | -10.69324378 | -10.40731278 | -6.28944807 |

**AcOH-HFIP-INT3***
***E*** = -5299.43
***H*** = -5089.23
***G*** = -5145.47
***N*imag** = 0

| | | | |
|---|---|---|---|
| H | -3.25504410 | -7.70735998 | 2.91748185 |
| H | 0.80516366 | -7.66488359 | 1.37931671 |
| H | -0.32312266 | -8.97413814 | 0.95275060 |
| H | -0.55491977 | -7.34218161 | 0.28239436 |
| C | -4.52749072 | -5.90246692 | 5.52103209 |
| C | -3.74681896 | -5.40453639 | 4.33672327 |
| O | -2.41635620 | -5.80610198 | 4.43413296 |
| O | -4.16625779 | -4.76414338 | 3.39477868 |
| H | -5.57564927 | -5.62092609 | 5.41453663 |
| H | -4.11307209 | -5.47339105 | 6.44019851 |
| H | -4.43373873 | -6.99151999 | 5.59556370 |
| H | -0.04293896 | -4.43618378 | 2.06369406 |
| C | -1.12671522 | -3.87728756 | 3.57338451 |
| O | -0.21095166 | -3.55913589 | 2.49602922 |
| H | -0.20609971 | -2.50115693 | 4.95611542 |
| H | -2.03472424 | -3.27734009 | 3.44050351 |
| C | -0.48524255 | -3.55822815 | 4.93036314 |
| H | -1.19027276 | -3.75351213 | 5.74458507 |
| H | 0.41217751 | -4.16811109 | 5.08051034 |
| C | -0.66426410 | -8.31628932 | 3.62806278 |
| H | -1.29205485 | -8.09010852 | 4.49430322 |
| H | 0.37455599 | -8.06620087 | 3.87067338 |
| H | -0.72120143 | -9.39185873 | 3.42718348 |
| C | -1.50141468 | -5.34790900 | 3.44858462 |

| | | | |
|---|---|---|---|
| N | -0.92558599 | -6.07595364 | 2.60200081 |
| C | -1.12925077 | -7.53459063 | 2.37846751 |
| C | -2.60714005 | -7.84284589 | 2.04834542 |
| H | -2.69419737 | -8.88474133 | 1.72156846 |
| H | -2.96349576 | -7.19697011 | 1.23883313 |
| C | -0.24354385 | -7.90348159 | 1.17054421 |
| C | 0.41160748 | -0.14690424 | 0.77372263 |
| F | 0.97992181 | -0.57418722 | -0.39171440 |
| F | 1.12675068 | -0.69523673 | 1.79905030 |
| F | 0.57334514 | 1.20754804 | 0.83338315 |
| C | -1.08576254 | -0.56458610 | 0.80048469 |
| O | -1.23909638 | -1.94690930 | 0.62113351 |
| C | -1.83874829 | -0.06848847 | 2.06717816 |
| F | -1.79269023 | 1.28770221 | 2.20419380 |
| F | -3.15256232 | -0.42734891 | 1.98366775 |
| F | -1.34321905 | -0.61731074 | 3.21802201 |
| H | -1.55610857 | -0.05455890 | -0.04725573 |
| H | -0.81216106 | -2.47364096 | 1.36403192 |

**3AcOH-TS1**

***E*** = -5916.50

***H*** = -5669.19

***G*** = -5730.61

***N*imag** = -358.1304

| | | | |
|---|---|---|---|
| C | 4.80922324 | -3.51639242 | -0.29545217 |
| H | 5.79855301 | -3.23377735 | -0.67296539 |
| H | 4.15786880 | -3.65570662 | -1.16592668 |
| C | 0.51554715 | 2.02993461 | -0.64652306 |
| C | 2.41333829 | 0.31238582 | 2.45595601 |
| O | 3.01387250 | 0.68834430 | 1.36345174 |
| H | 0.90593539 | 1.85288300 | 2.54538023 |
| H | 2.93489327 | -0.43539184 | 3.06399616 |
| C | 1.57961859 | 1.31389311 | 3.21608929 |
| H | 1.00868337 | 0.82656963 | 4.01157320 |
| H | 2.28471261 | 2.02338091 | 3.66824033 |
| O | 1.86200564 | 2.11305930 | -0.56853330 |
| C | -0.03681938 | 2.76287945 | -1.84875148 |
| H | -1.12646988 | 2.70868742 | -1.85173786 |
| H | 0.28795425 | 3.80905481 | -1.83004899 |
| H | 1.59634871 | -3.99383677 | 0.09021972 |
| H | 0.11853477 | -4.49032430 | -0.76564461 |
| H | 0.09813680 | -4.29225606 | 1.00259088 |
| C | -1.44370114 | -2.25838018 | -0.10804280 |
| H | -1.71321664 | -1.20269560 | -0.20504377 |
| H | -1.87116511 | -2.81388424 | -0.94840107 |
| H | -1.86134267 | -2.65217382 | 0.82356002 |
| C | 1.08806292 | -0.98973777 | 1.81449634 |

| | | | |
|---|---|---|---|
| N | 0.63424418 | -1.65803376 | 0.98034649 |
| C | 0.08951590 | -2.42607126 | -0.13294679 |
| C | 0.69739399 | -1.83468581 | -1.42416780 |
| H | 0.32575455 | -2.40802213 | -2.27875296 |
| H | 1.78924575 | -1.89172400 | -1.40438606 |
| C | 0.50693594 | -3.89738372 | 0.06773330 |
| H | 0.39858787 | -0.78989497 | -1.54130469 |
| H | 0.36026163 | 2.31375766 | -2.76691322 |
| O | 4.11594844 | -1.24895953 | -0.08788763 |
| H | 2.20267555 | 1.59038963 | 0.23290405 |
| O | -0.16563999 | 1.41372422 | 0.16825626 |
| O | 3.98412720 | -2.53952585 | 1.77127832 |
| H | 4.88080127 | -4.44572132 | 0.27140029 |
| H | 3.73515095 | -0.53870033 | 0.53400241 |
| C | 4.26822664 | -2.41099983 | 0.58327923 |
| C | 5.70834802 | 2.21655912 | 2.98828975 |
| O | 5.35933633 | 2.06769244 | 1.68526141 |
| C | 7.00471042 | 2.97488050 | 3.14847769 |
| H | 7.24222082 | 3.08839783 | 4.20715896 |
| H | 7.81179210 | 2.43460224 | 2.64038459 |
| H | 6.91883338 | 3.95945980 | 2.67503337 |
| H | 4.49766916 | 1.55973988 | 1.63775334 |
| O | 5.02965740 | 1.77438897 | 3.90930611 |

**3AcOH-TS1**
***E*** = -7172.54
***H*** = -6919.83
***G*** = -6995.79
***N*imag** = -327.5874

| | | | |
|---|---|---|---|
| F | 0.87791313 | 2.40711972 | 1.00430418 |
| F | -0.00080278 | 2.55119960 | -1.00730373 |
| F | 1.38960055 | 4.10680580 | -0.29883960 |
| C | 2.34853515 | 1.94205717 | -0.80481096 |
| C | 2.28631515 | -1.08666959 | 2.44697353 |
| O | 3.06999576 | -0.50451977 | 1.58600490 |
| H | 1.15080700 | 0.65033490 | 3.05485048 |
| H | 2.57232055 | -2.09461564 | 2.76893860 |
| C | 1.60166738 | -0.24242411 | 3.49650834 |
| H | 0.84684758 | -0.81710242 | 4.03943743 |
| H | 2.37881569 | 0.07449992 | 4.20510699 |
| O | 2.09094661 | 0.56197588 | -0.66982612 |
| C | 2.69161597 | 2.20283793 | -2.28978779 |
| F | 2.92420382 | 3.52360979 | -2.53118650 |
| F | 3.82555151 | 1.51687441 | -2.61937103 |
| H | -0.98392860 | -4.12210579 | -0.48408053 |
| H | -2.29730607 | -3.35540038 | -1.40640962 |
| H | -2.25084848 | -3.19771097 | 0.36517499 |

| | | | |
|---|---|---|---|
| C | -1.70937082 | -0.70151095 | -0.73536645 |
| H | -1.10383873 | 0.20389570 | -0.82428513 |
| H | -2.37533752 | -0.76717470 | -1.60097485 |
| H | -2.31707065 | -0.64227756 | 0.17251449 |
| C | 0.75117038 | -1.71791828 | 1.38151459 |
| N | 0.06241809 | -1.83568067 | 0.45480432 |
| C | -0.81168852 | -1.95626478 | -0.71318629 |
| C | 0.09550777 | -2.02258810 | -1.95982925 |
| H | -0.54259864 | -2.08491429 | -2.84639345 |
| H | 0.73706464 | -2.90697401 | -1.92633902 |
| C | -1.63809729 | -3.24684343 | -0.53993327 |
| H | 0.71743889 | -1.12664053 | -2.02991714 |
| F | 1.70623165 | 1.78931637 | -3.13474200 |
| H | 3.21385934 | 2.27129657 | -0.21428429 |
| H | 2.39949336 | 0.25842676 | 0.22786218 |
| C | 1.13772573 | 2.76801333 | -0.29082866 |
| F | 3.02691924 | -3.38264172 | -0.54125796 |
| F | 4.97653877 | -4.14234824 | -1.22061291 |
| F | 3.59951617 | -3.19811455 | -2.65933783 |
| C | 4.66441341 | -1.73396284 | -1.04644307 |
| O | 5.04794744 | -1.67967924 | 0.30701184 |
| C | 5.89566760 | -1.35645977 | -1.90392810 |
| F | 5.61803079 | -1.39628511 | -3.23828288 |
| F | 6.28070114 | -0.07988262 | -1.60307325 |
| F | 6.96444414 | -2.16812505 | -1.67444876 |
| H | 3.87777593 | -1.00681602 | -1.29523665 |
| C | 4.07905904 | -3.13327190 | -1.38286036 |
| H | 4.26640985 | -1.33280726 | 0.83934561 |
| F | 5.83722273 | 0.16200142 | 3.58809759 |
| F | 6.54631148 | 2.23699750 | 3.76798357 |
| F | 7.78059748 | 0.73680309 | 2.72656500 |
| C | 5.76310861 | 1.46007368 | 1.59839026 |
| O | 4.41678575 | 1.80354883 | 1.84994748 |
| C | 6.39203972 | 2.61597260 | 0.78560825 |
| F | 7.70565778 | 2.38514707 | 0.51031688 |
| F | 5.73717645 | 2.73719967 | -0.40931441 |
| F | 6.30010992 | 3.81574455 | 1.42177728 |
| H | 5.86341936 | 0.55302896 | 0.98574977 |
| C | 6.50182648 | 1.16282884 | 2.93271156 |
| H | 3.87900795 | 0.96491481 | 1.88059402 |

**P**
***E*** = -3857.11
***H*** = -3690.30
***G*** = -3729.05
***N*imag** = 0

| | | | |
|---|---|---|---|
| O | 0.59069380 | 0.43453832 | -2.73434694 |

| | | | |
|---|---|---|---|
| H | 3.61673724 | 1.06577308 | -2.31020397 |
| H | 0.89600543 | -0.50012466 | 0.55327501 |
| H | 3.33729297 | -0.46913172 | -1.48114926 |
| H | 3.01422836 | -0.34169193 | -3.23885900 |
| C | 2.97014662 | 0.18154039 | -2.28270298 |
| C | 1.54945119 | 0.60262783 | -1.99833030 |
| O | -1.99525313 | 0.75217794 | 0.22093980 |
| C | 0.12866892 | 1.70518554 | -0.37513180 |
| O | 1.45817564 | 1.21353482 | -0.77241838 |
| H | -0.61199983 | 3.18015916 | 1.00551757 |
| H | -0.37348518 | 2.09870451 | -1.26150323 |
| C | 0.35589926 | 2.79576962 | 0.66975843 |
| H | 0.89733576 | 2.39803665 | 1.53507748 |
| H | 0.93067923 | 3.61946377 | 0.23554444 |
| H | -2.25516679 | -0.65505006 | 2.35719039 |
| H | -0.73905222 | -0.89663429 | 3.25780589 |
| H | -1.85930449 | -2.25707626 | 3.02063035 |
| C | -1.69551333 | -2.39789638 | 0.24632784 |
| H | -1.17514367 | -2.65733091 | -0.68245225 |
| H | -2.10325510 | -3.31572927 | 0.68433284 |
| H | -2.52291867 | -1.72465449 | 0.00945017 |
| C | -0.76636554 | 0.57527859 | 0.17964762 |
| N | -0.11584325 | -0.51946020 | 0.62824018 |
| C | -0.72000941 | -1.73928561 | 1.24441570 |
| C | 0.45379120 | -2.69078663 | 1.54191021 |
| H | 0.98092404 | -2.96247876 | 0.61929318 |
| H | 1.16797104 | -2.22823917 | 2.23390907 |
| C | -1.44157140 | -1.35778109 | 2.55434038 |
| H | 0.07826025 | -3.60953180 | 2.00282831 |

**MeNC**
***E*** = -797.04
***H*** = -766.55
***G*** = -784.70
***N*imag** = 0

| | | | |
|---|---|---|---|
| H | 3.78914907 | -3.95226089 | -0.81842591 |
| H | 4.48240493 | -2.41540857 | -0.22473593 |
| C | 2.61084531 | -1.67949573 | -2.63318960 |
| H | 5.17502910 | -3.20945732 | -1.66869016 |
| N | 3.35001078 | -2.27746898 | -1.95008640 |
| C | 4.25396790 | -3.00794811 | -1.11490629 |

**Formaldehyde**
***E*** = -498.46
***H*** = -479.89
***G*** = -495.49
***N*imag** = 0

| | | | |
|---|---|---|---|
| C | 0.00000000 | 0.00000000 | 1.05872327 |
| O | 0.00000000 | -0.00000000 | -0.16080789 |
| H | 0.00000000 | -0.94343859 | 1.64126731 |
| H | -0.00000000 | 0.94343859 | 1.64126731 |

**MeNC-formaldehyde-AcOH-imidate**

***E*** = -2362.19
***H*** = -2268.14
***G*** = -2299.05
***N*imag** = 0

| | | | |
|---|---|---|---|
| H | 0.94341152 | -1.83325584 | -1.26960064 |
| O | -0.32253548 | 0.17446951 | -0.45199625 |
| O | 0.67562575 | -0.37746412 | 1.52860215 |
| C | -1.65667854 | -0.97744734 | 1.14582137 |
| H | 2.35767495 | -1.68218092 | -2.35313633 |
| C | 0.89021702 | 0.74527356 | -0.93399615 |
| N | 1.87275295 | 0.10220479 | -1.38530180 |
| H | 2.59462955 | 2.02736057 | -1.73713740 |
| C | 0.85469980 | 2.25153020 | -0.92009209 |
| O | 2.03770503 | 2.80490137 | -1.50846543 |
| C | -0.31078837 | -0.38998793 | 0.82025556 |
| H | -0.03630876 | 2.59184096 | -1.46745664 |
| H | 0.75246162 | 2.58819632 | 0.12345936 |
| C | 1.92340056 | -1.35949839 | -1.40206175 |
| H | 2.59116570 | -1.69448740 | -0.59949748 |
| H | -1.63049269 | -1.42417216 | 2.14039970 |
| H | -2.41986860 | -0.19250389 | 1.10524918 |
| H | -1.92078604 | -1.73393050 | 0.39861791 |

**MeNC-formaldehyde-HFIP-imidate**

***E*** = -2781.38
***H*** = -2685.23
***G*** = -2723.21
***N*imag** = 0

| | | | |
|---|---|---|---|
| H | 2.09113712 | -1.97536375 | -0.88178754 |
| F | 0.56342757 | -0.15041102 | 2.59071084 |
| H | -0.73552129 | 1.36473875 | 0.92164412 |
| C | -2.24177818 | 0.10169247 | 0.07761421 |
| H | 1.02636092 | -1.69479775 | -2.27321338 |
| F | -0.82407195 | -1.75985407 | 2.02259963 |
| C | -0.68668442 | -0.41094303 | 2.11060836 |
| H | 2.62094862 | 1.92349811 | -1.64533308 |
| C | 1.11765014 | 2.04929541 | -0.44703544 |
| O | 2.18962671 | 2.66755139 | -1.16046115 |
| F | -3.23803004 | 0.59814786 | 0.85920297 |

| | | | |
|---|---|---|---|
| H | 0.16094731 | 2.49843965 | -0.75288884 |
| H | 1.24506357 | 2.21956869 | 0.63270616 |
| F | -2.51752154 | -1.20225786 | -0.18555717 |
| F | -2.26147026 | 0.77821311 | -1.10746344 |
| H | 2.79504539 | -1.60484577 | -2.47746591 |
| C | -0.85307547 | 0.29475099 | 0.74015943 |
| F | -1.58843571 | 0.04984768 | 3.01869993 |
| O | 0.12656094 | -0.24941227 | -0.14060028 |
| C | 1.09870234 | 0.55723965 | -0.73276976 |
| N | 1.96181660 | 0.05312455 | -1.50607705 |
| C | 1.96717601 | -1.38639266 | -1.79954280 |

# 6. Copies of NMR spectra

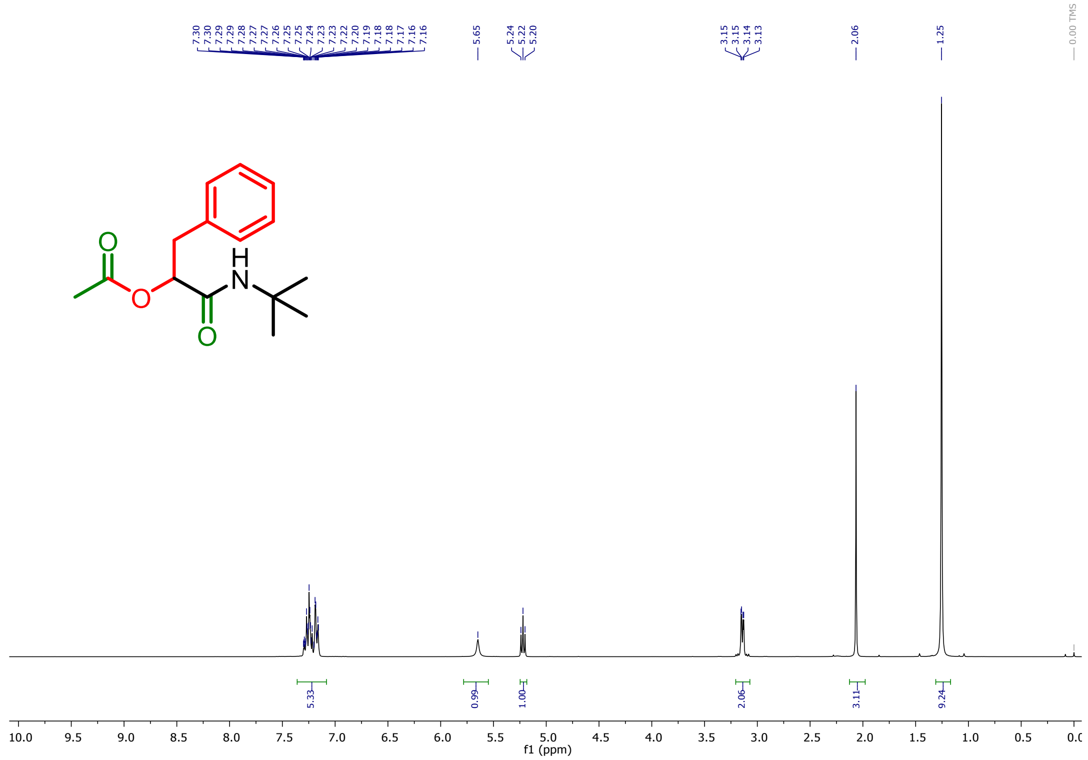


$^{1}H$ NMR spectrum of **6a** (300 MHz, $CDCl_3$).

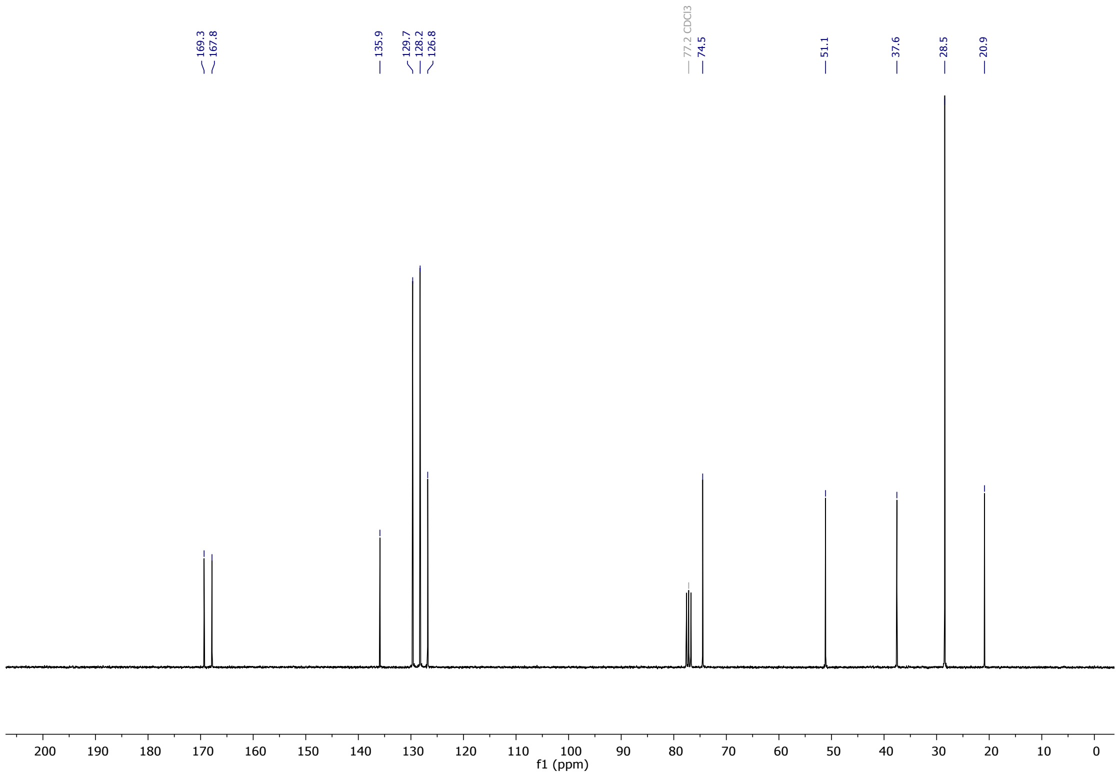


$^{13}C\{^{1}H\}$ NMR spectrum of **6a** (75 MHz, $CDCl_3$).

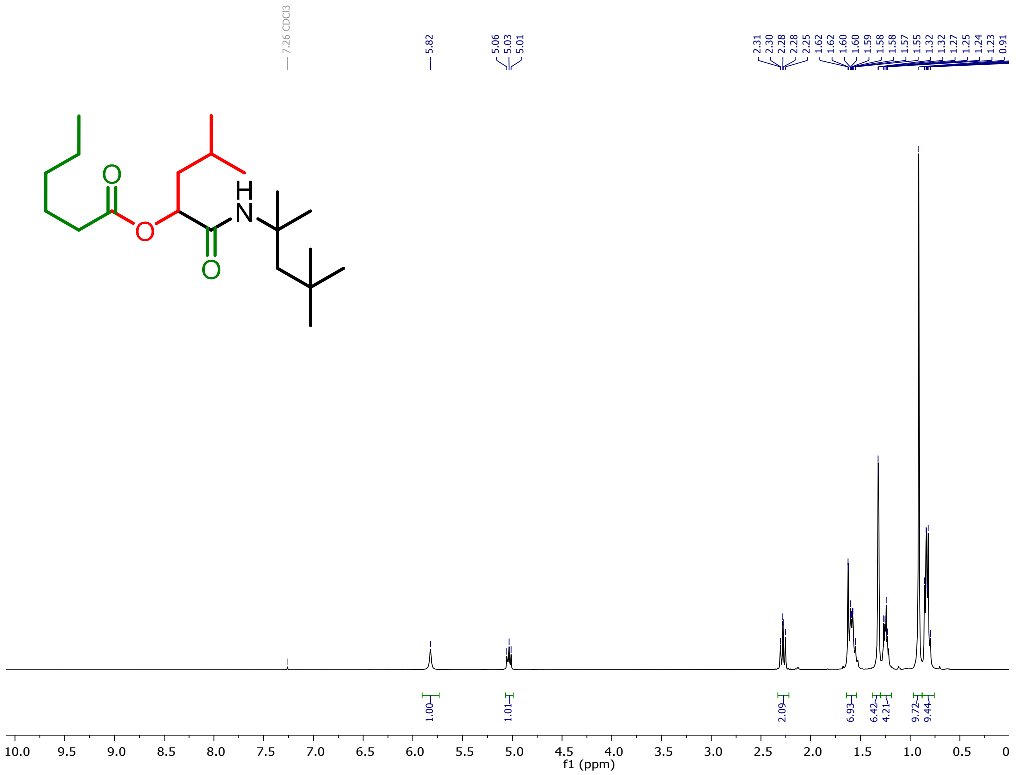

$^{1}H$ NMR spectrum of **6b** (300 MHz, $CDCl_3$).

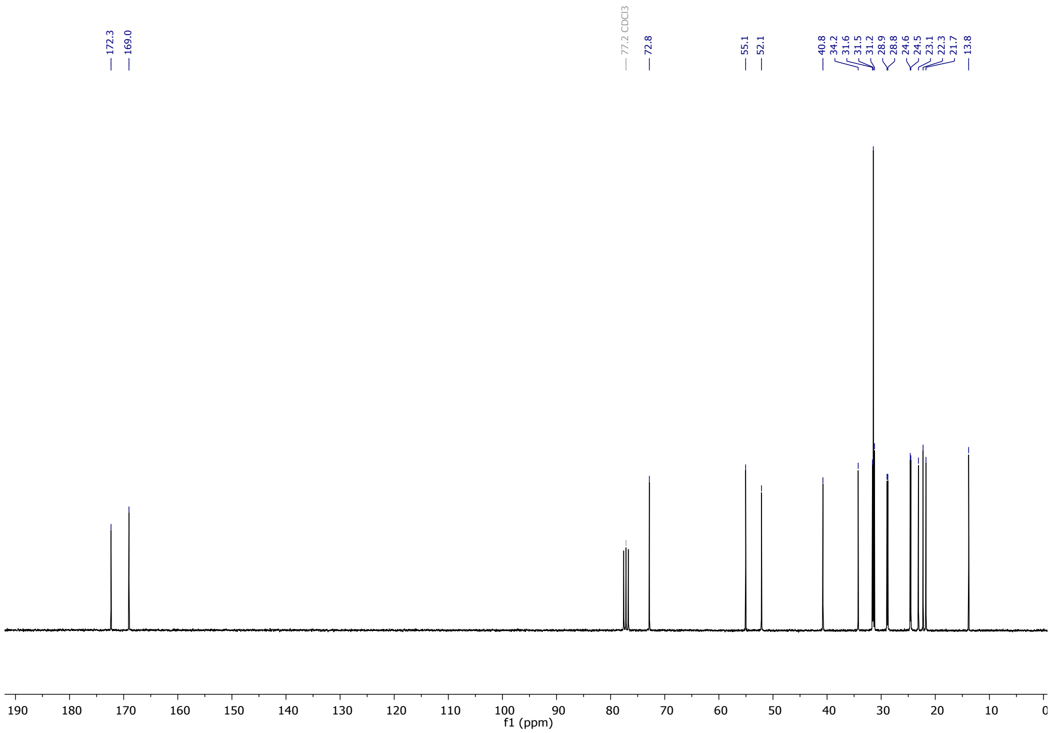

$^{13}C\{^{1}H\}$ NMR spectrum of **6b** (75 MHz, $CDCl_3$).

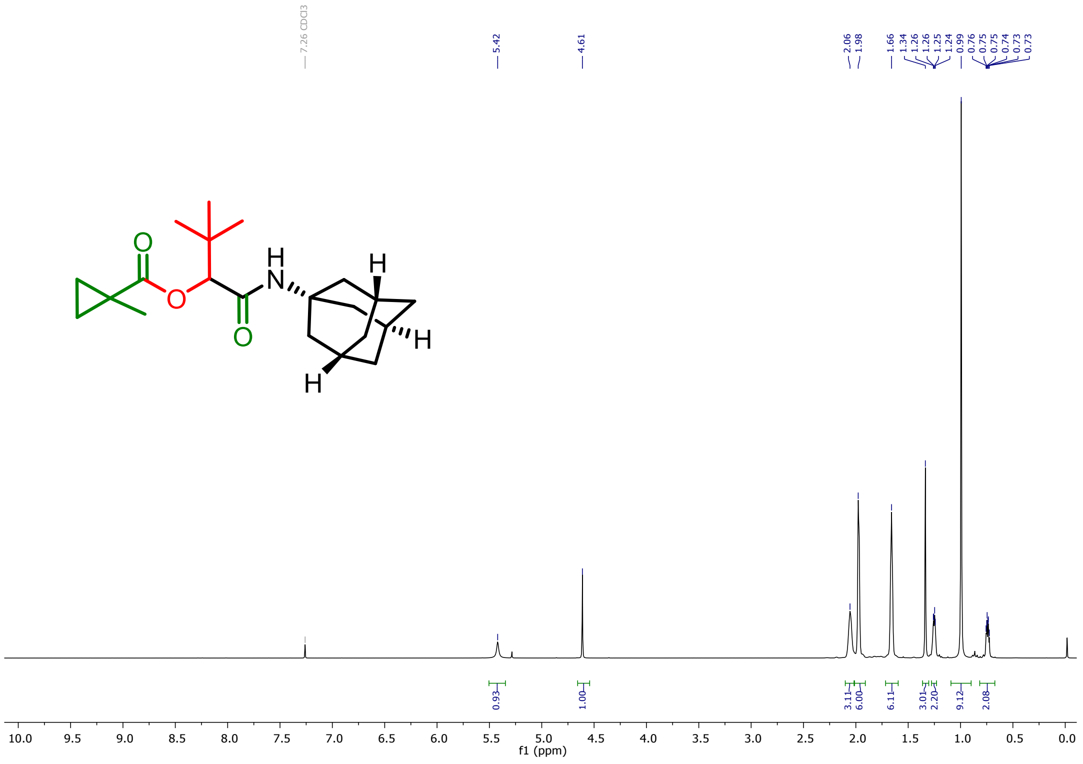

$^1H$ NMR spectrum of **6c** (300 MHz, $CDCl_3$).

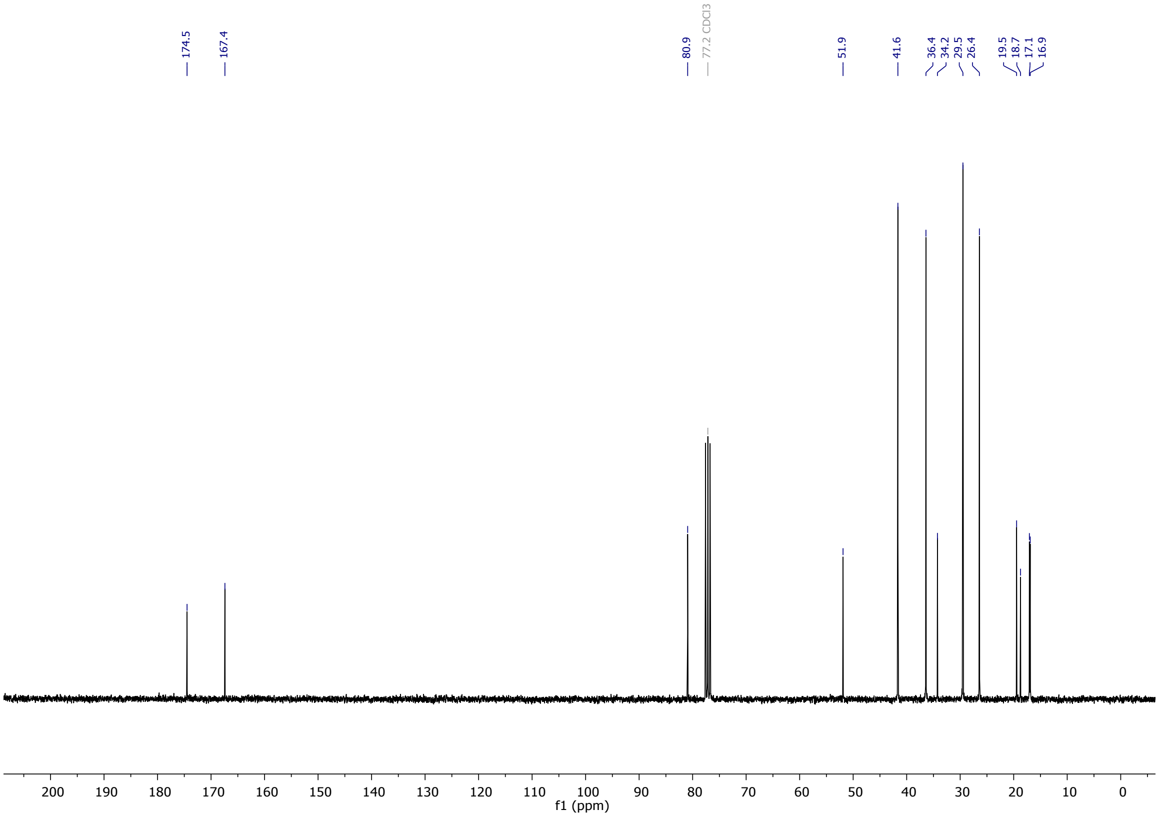

$^{13}C\{^1H\}$ NMR spectrum of **6c** (75 MHz, $CDCl_3$).

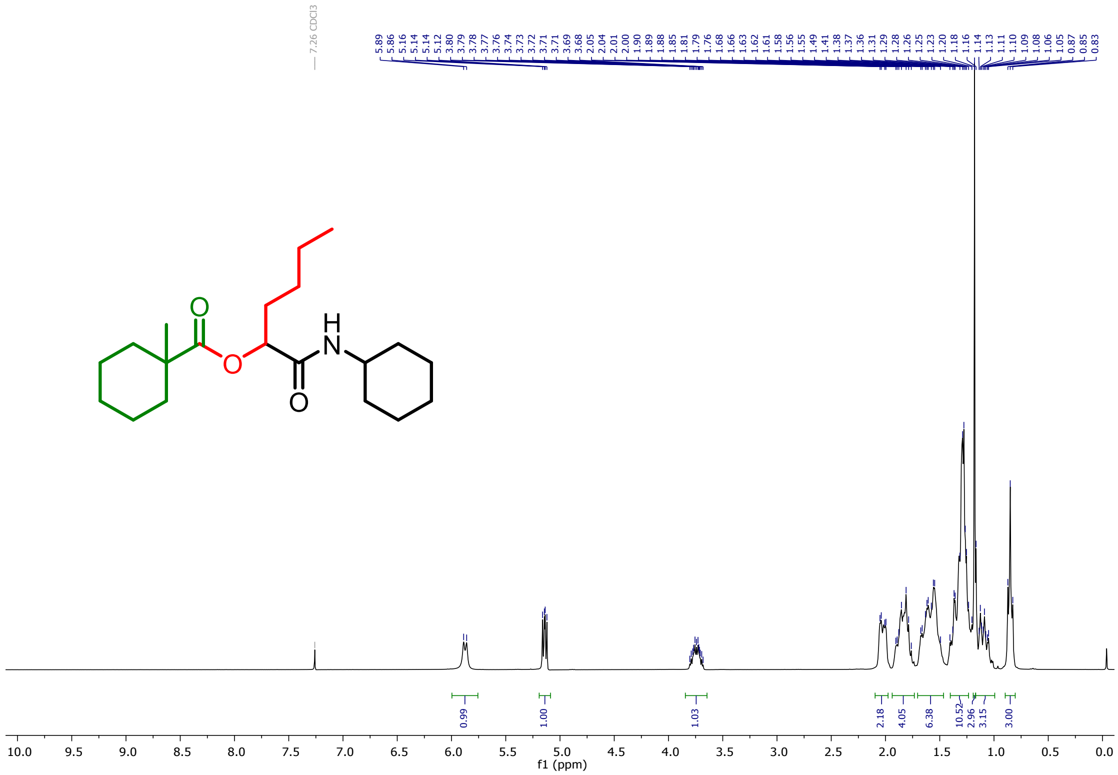

$^{1}H$ NMR spectrum of **6d** (300 MHz, $CDCl_3$).

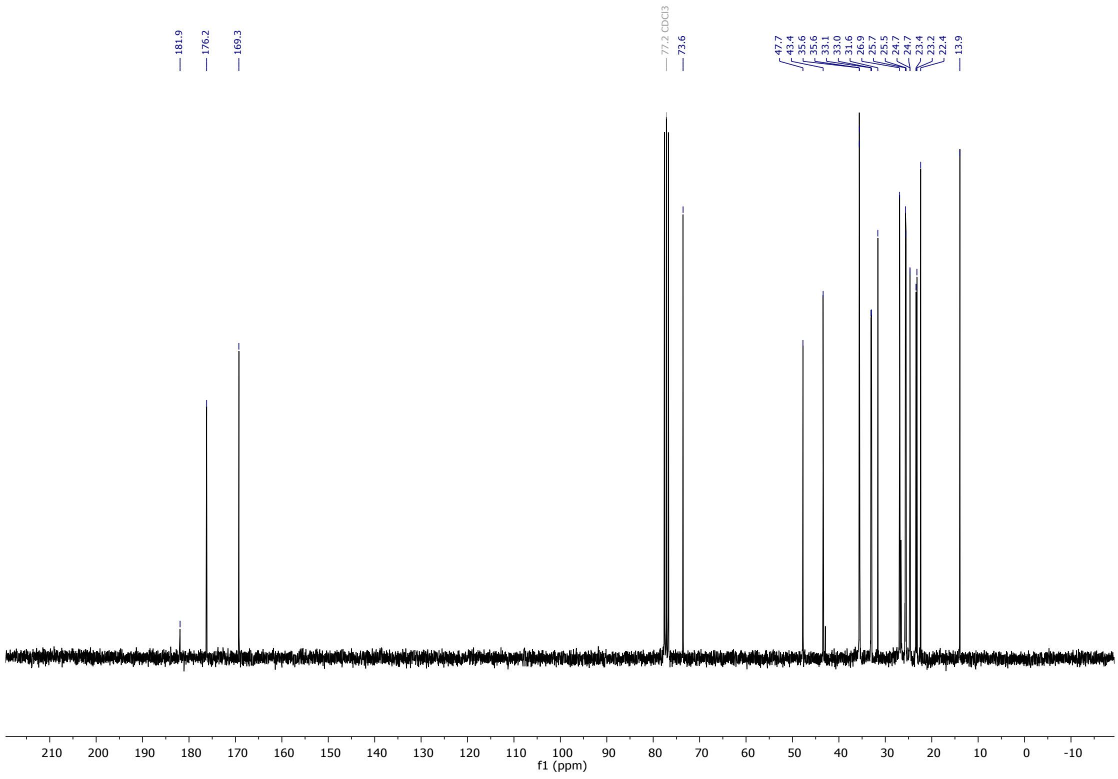

$^{13}C\{^{1}H\}$ NMR spectrum of **6d** (75 MHz, $CDCl_3$).

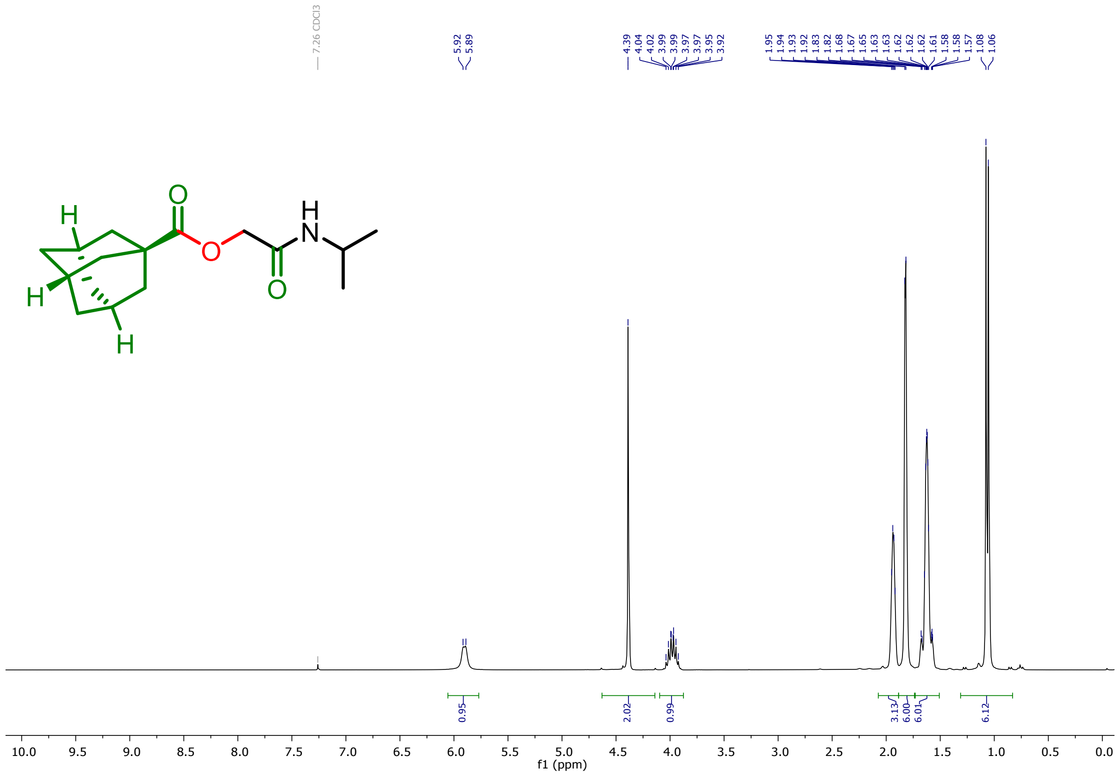

$^{1}H$ NMR spectrum of **6e** (300 MHz, $CDCl_3$).

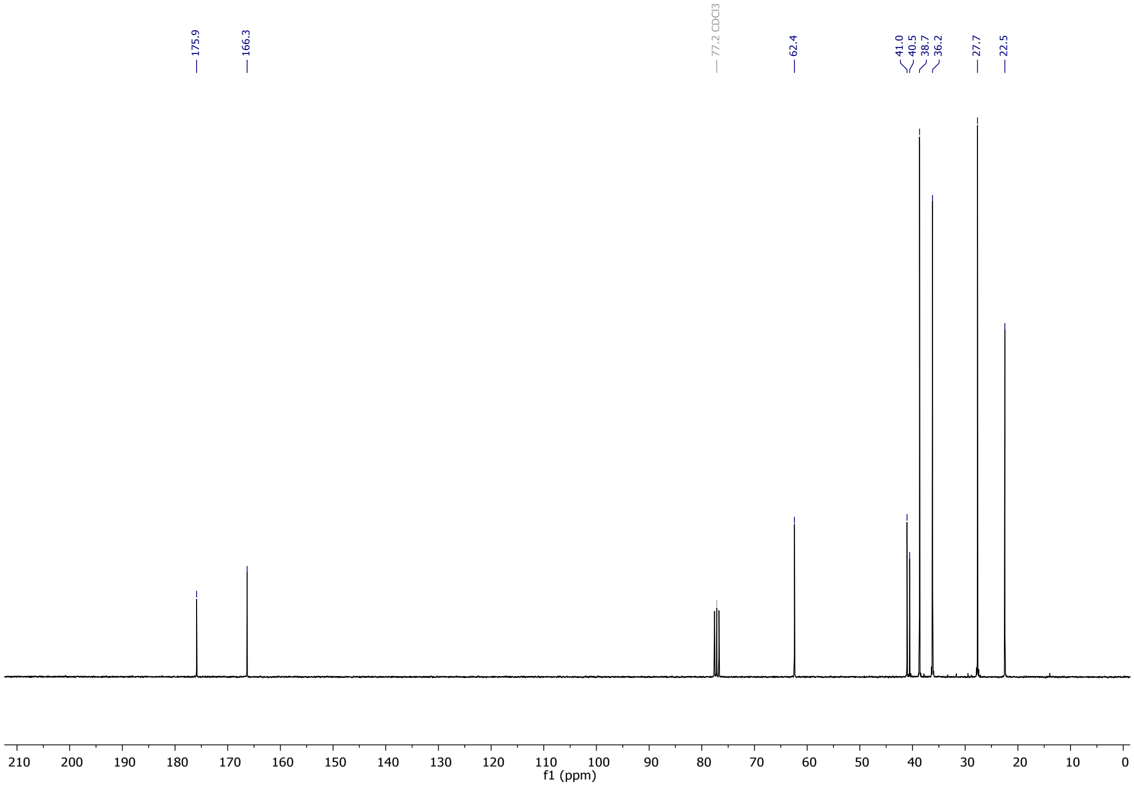

$^{13}C\{^{1}H\}$ NMR spectrum of **6e** (75 MHz, $CDCl_3$).

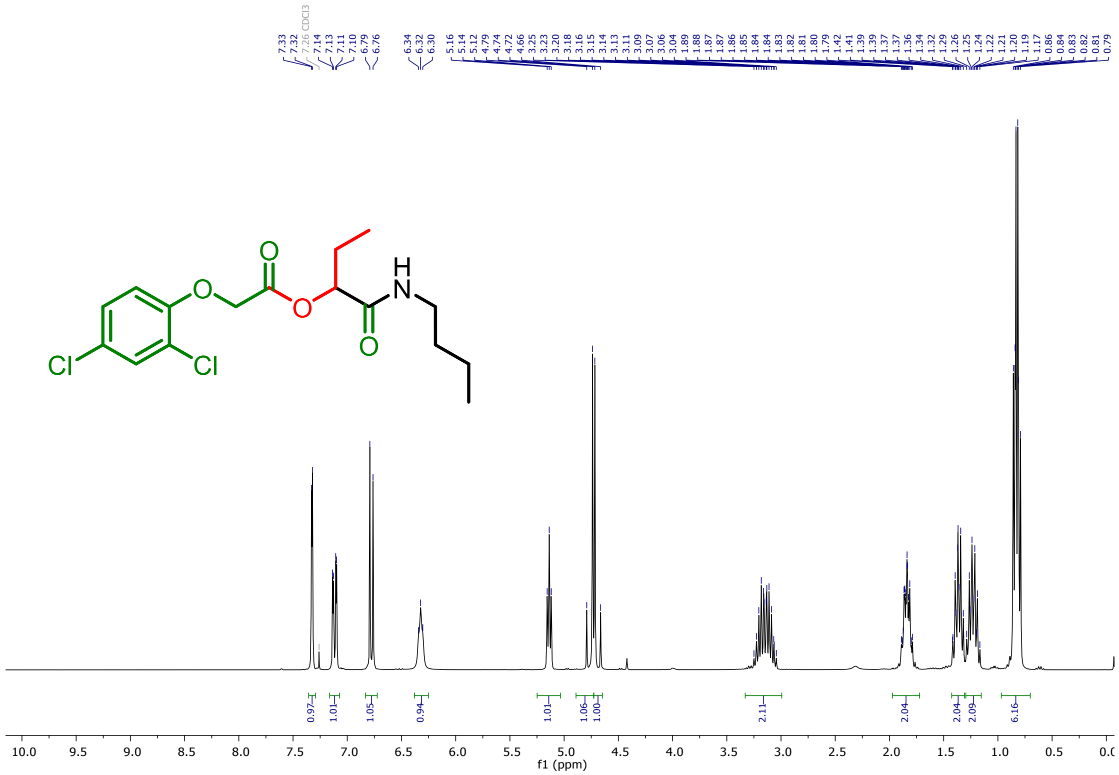

$^1H$ NMR spectrum of **6f** (300 MHz, $CDCl_3$).

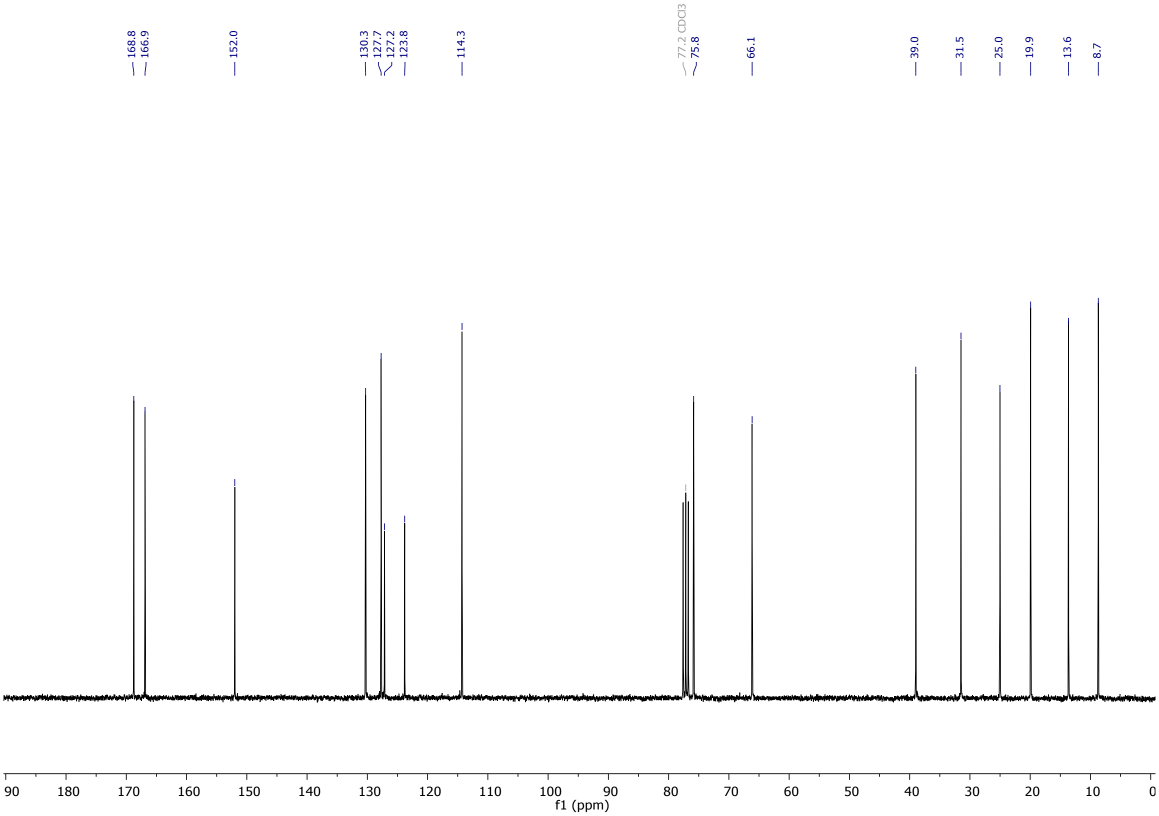

$^{13}C\{^1H\}$ NMR spectrum of **6f** (75 MHz, $CDCl_3$).

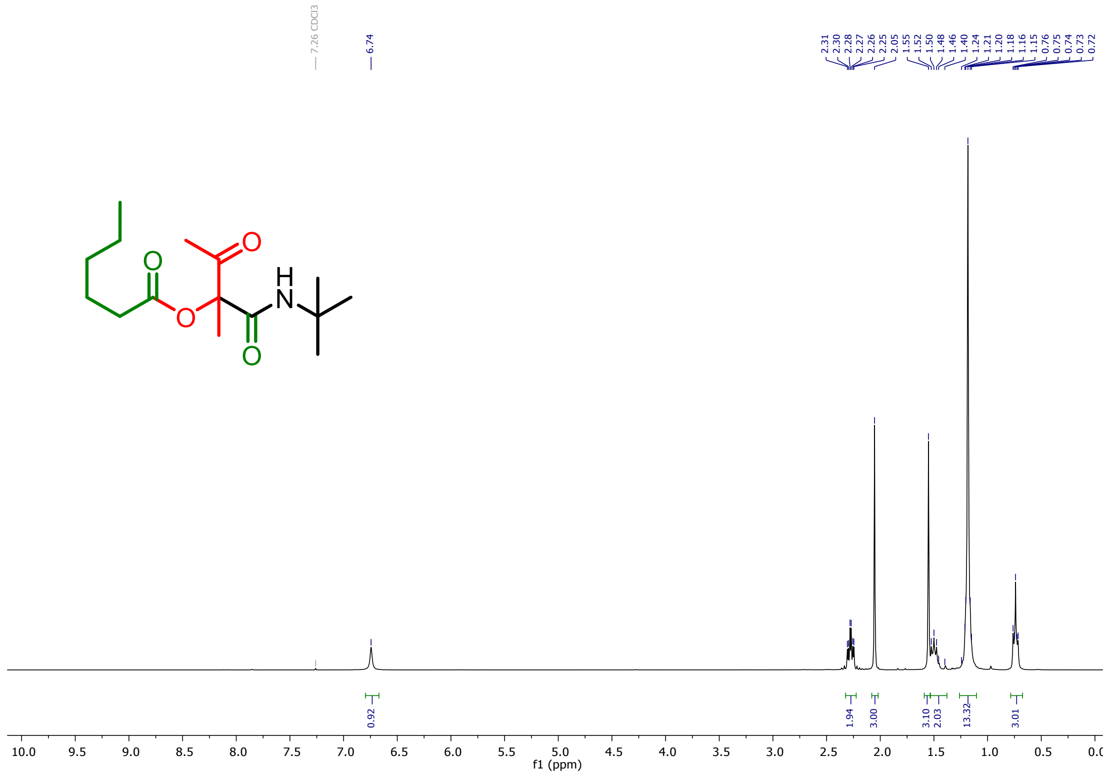


$^{1}$H NMR spectrum of **6g** (300 MHz, $CDCl_3$).

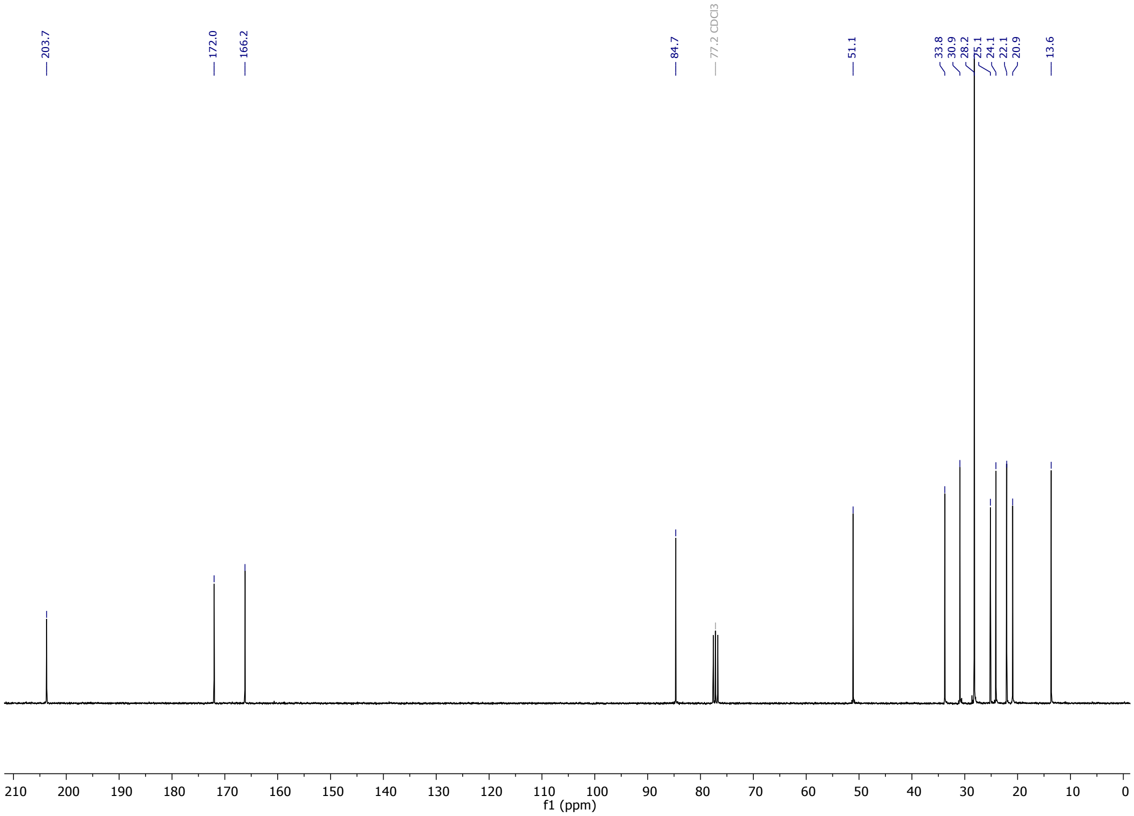


$^{13}$C{$^{1}$H} NMR spectrum of **6g** (75 MHz, $CDCl_3$).

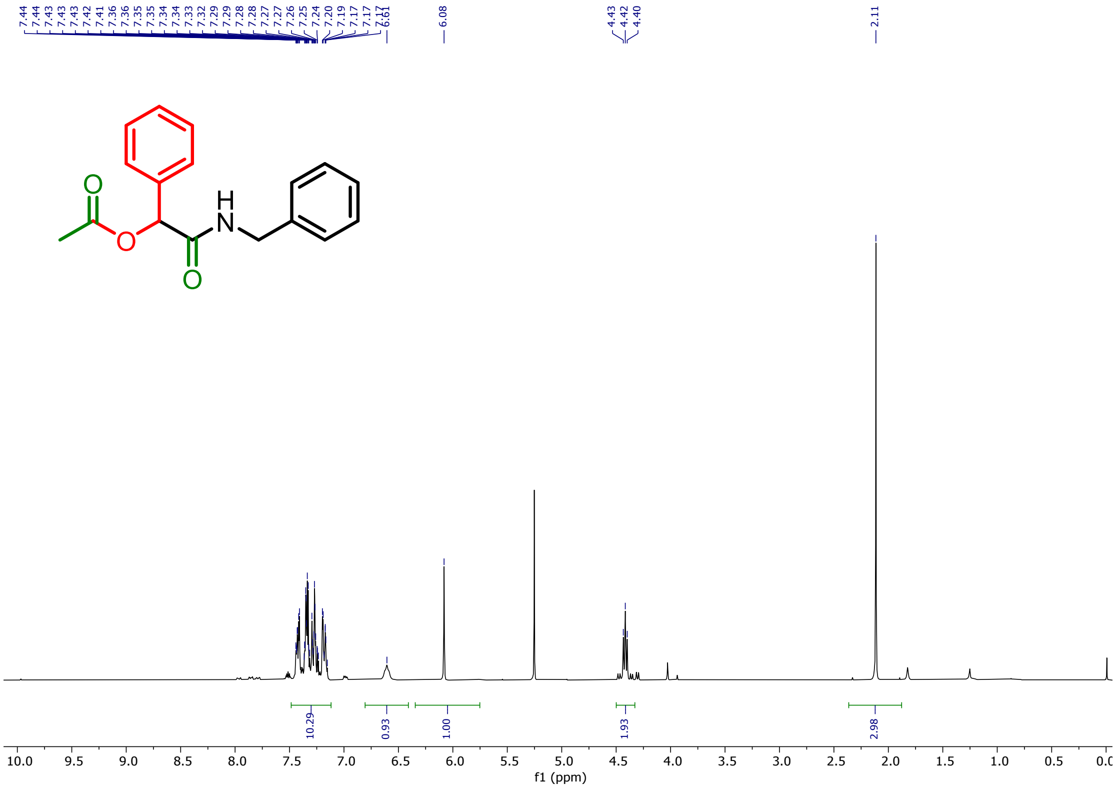


$^{1}H$ NMR spectrum of **6h** (300 MHz, $CDCl_3$).

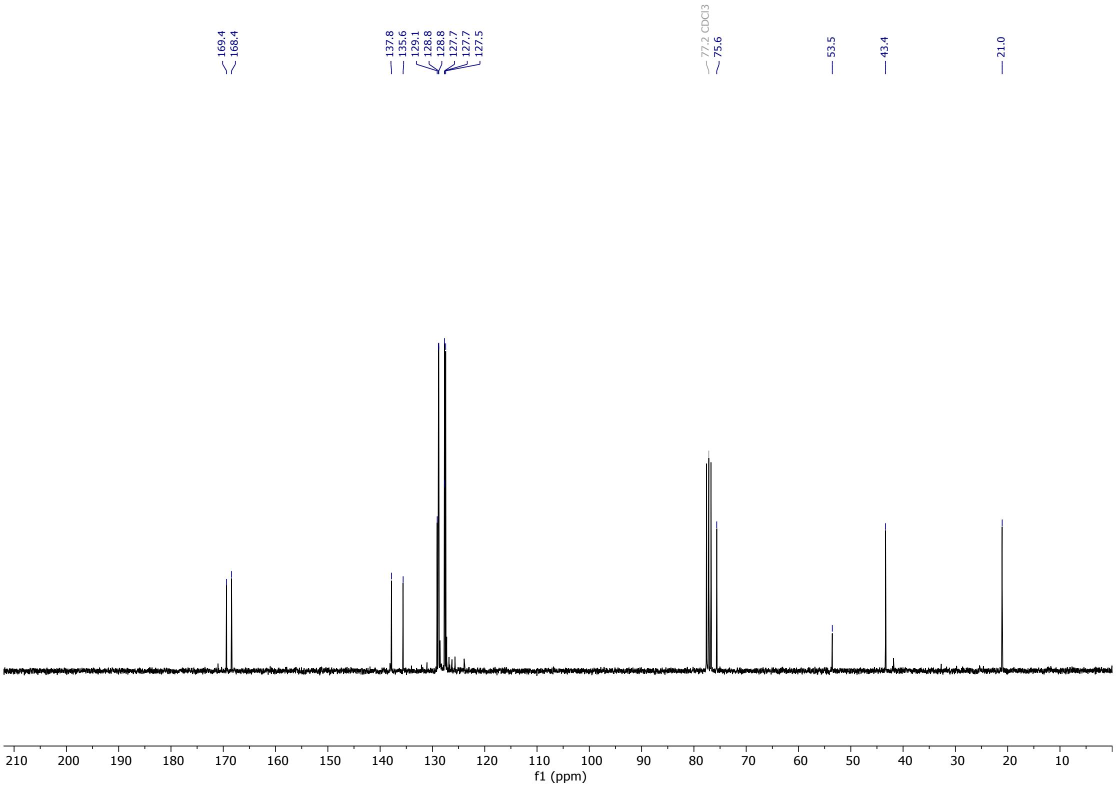


$^{13}C\{^{1}H\}$ NMR spectrum of **6h** (75 MHz, $CDCl_3$).

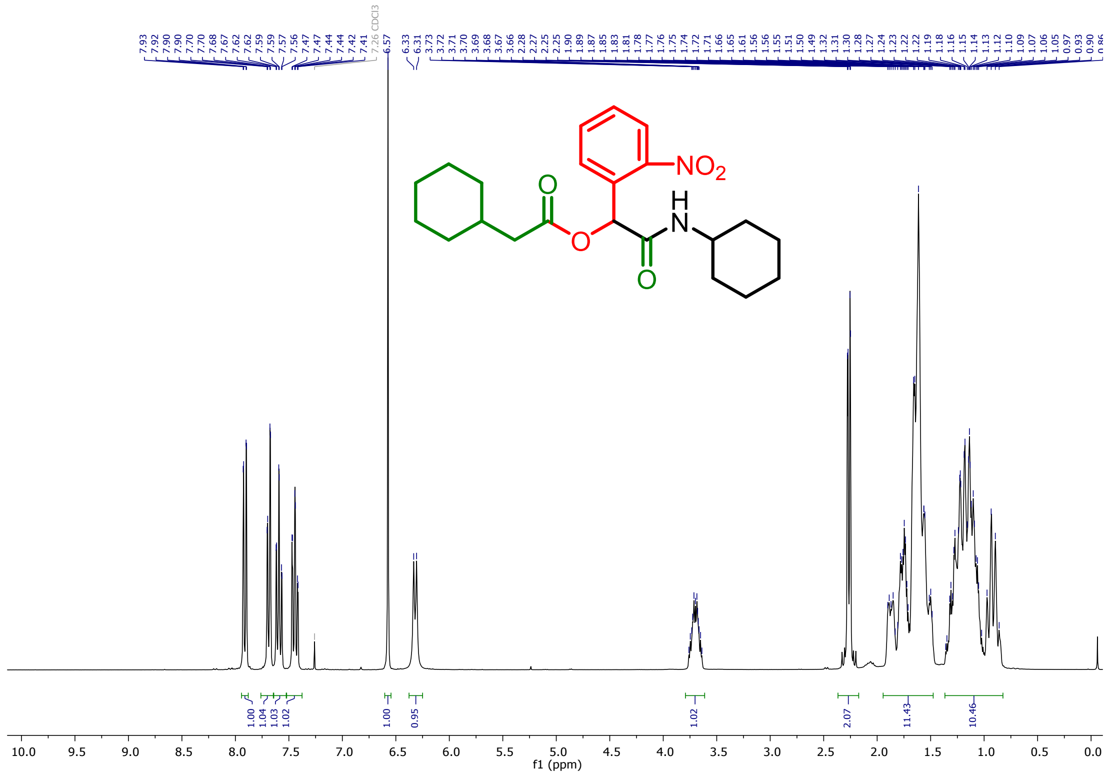


$^{1}$H NMR spectrum of **6i** (300 MHz, $CDCl_3$).

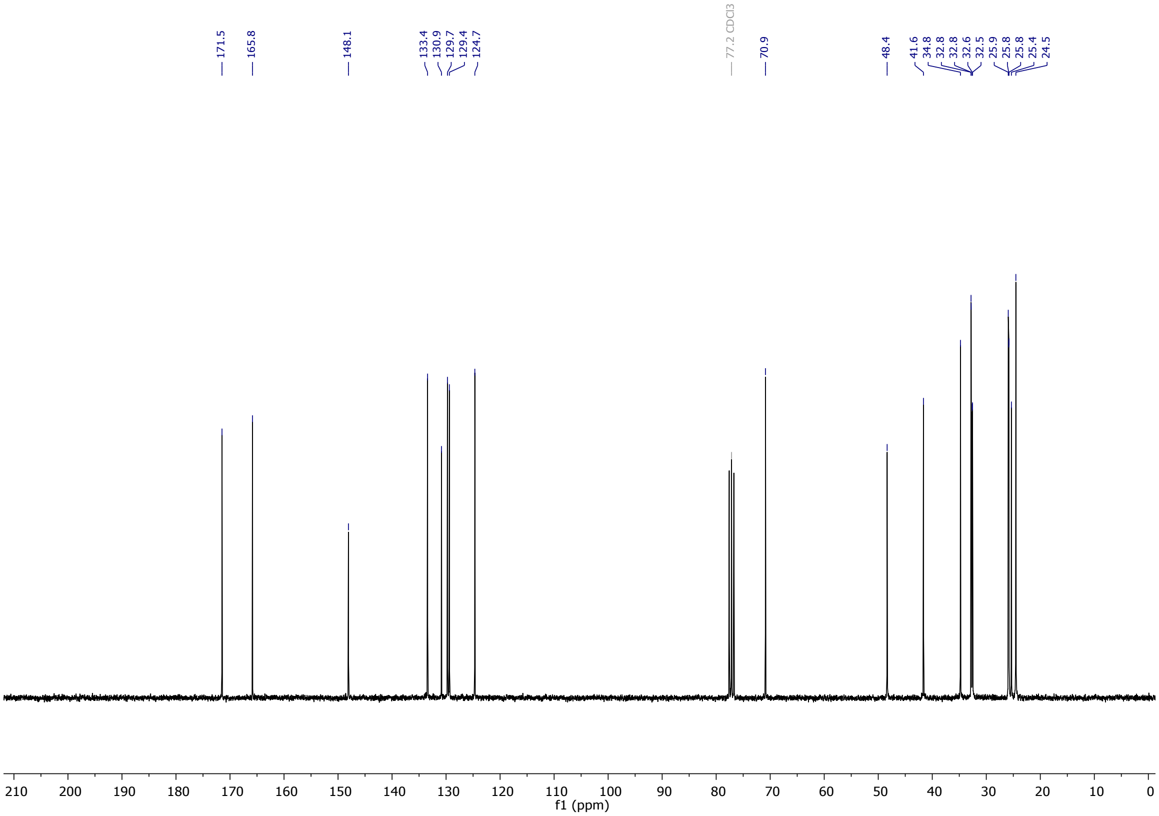


$^{13}$C{$^{1}$H} NMR spectrum of **6i** (75 MHz, $CDCl_3$).

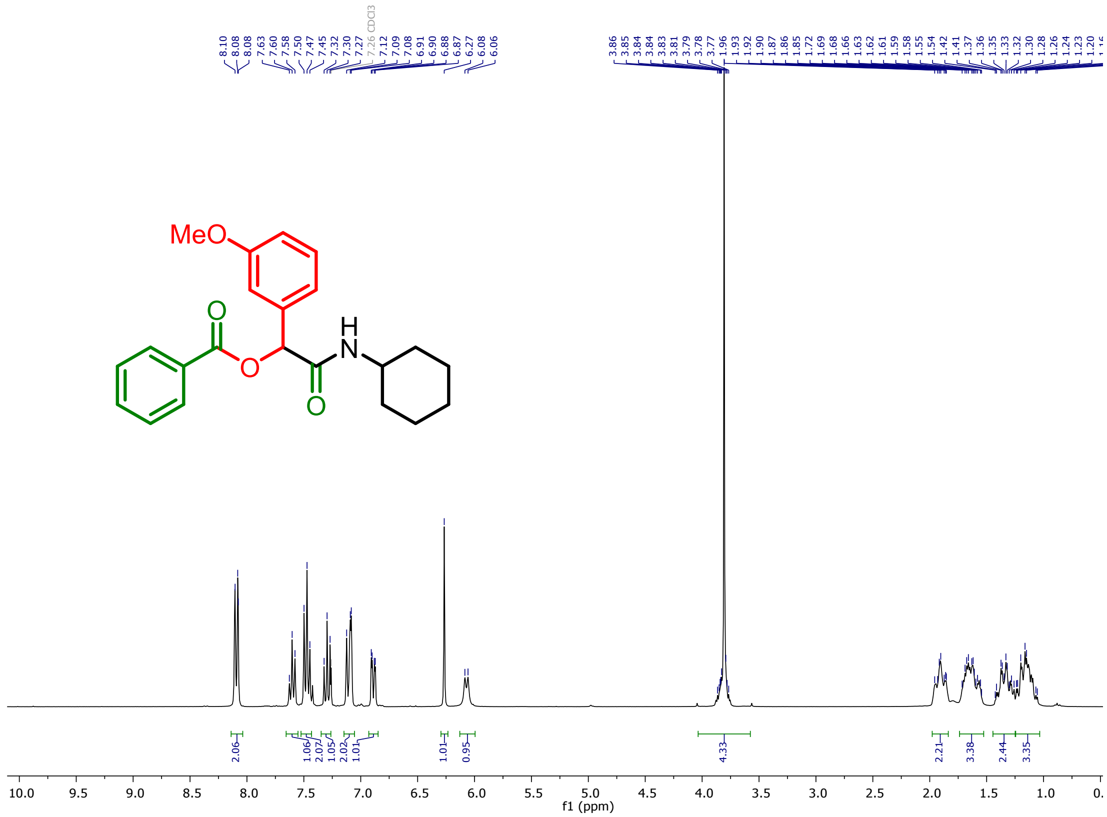


$^{1}$H NMR spectrum of **6j** (300 MHz, $CDCl_3$).

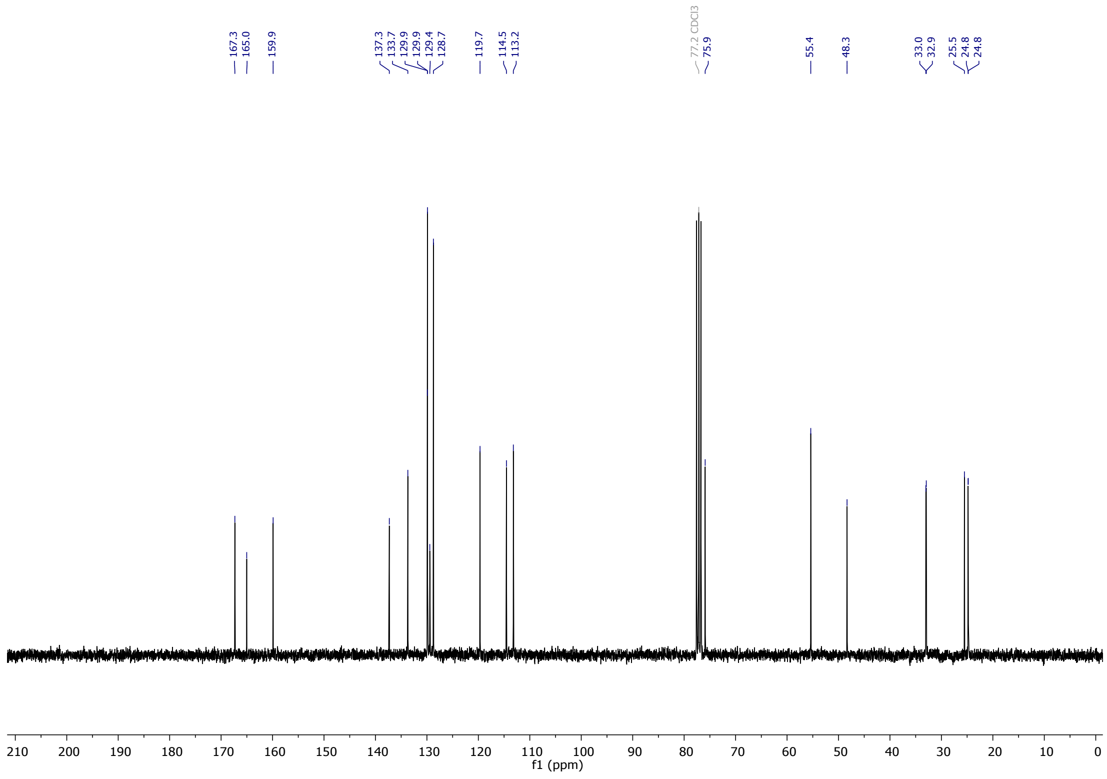


$^{13}$C{$^{1}$H} NMR spectrum of **6j** (75 MHz, $CDCl_3$).

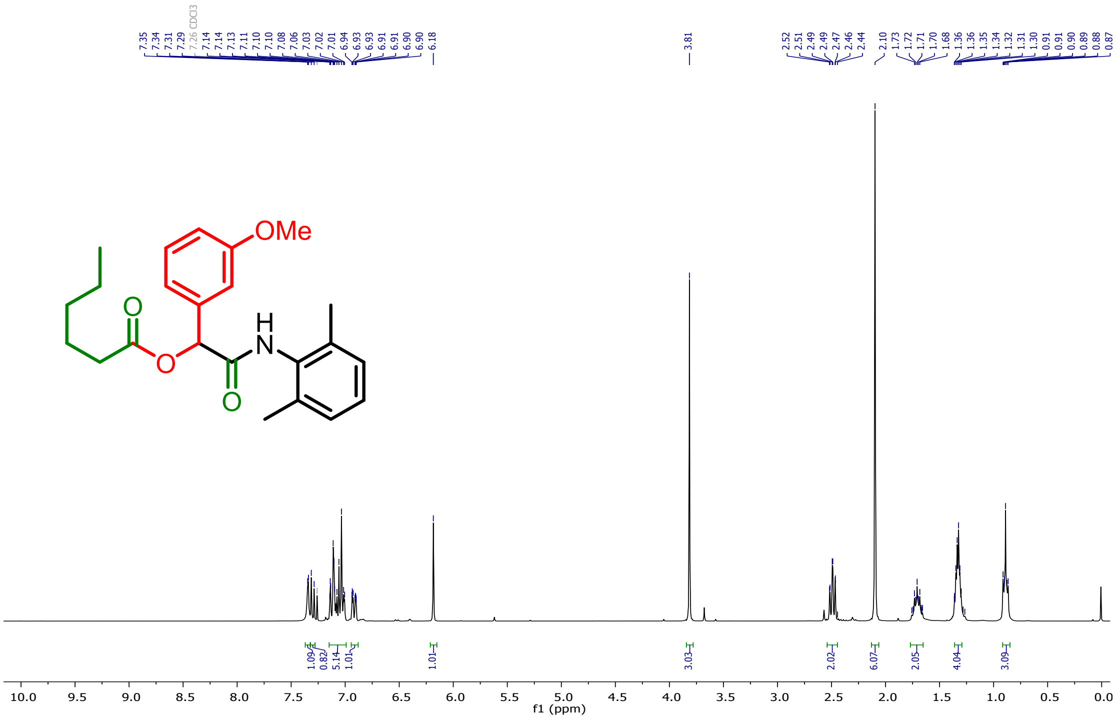


$^{1}H$ NMR spectrum of **6k** (300 MHz, $CDCl_3$).

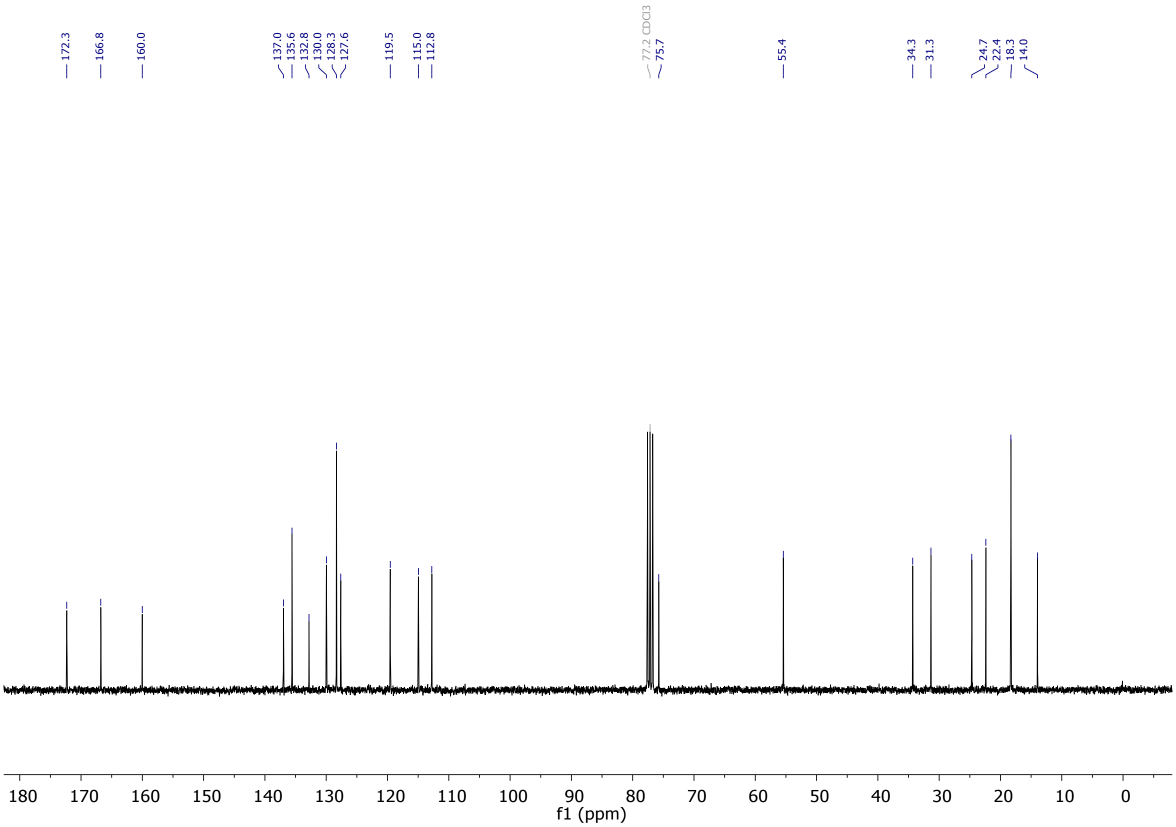


$^{13}C\{^{1}H\}$ NMR spectrum of **6k** (75 MHz, $CDCl_3$).

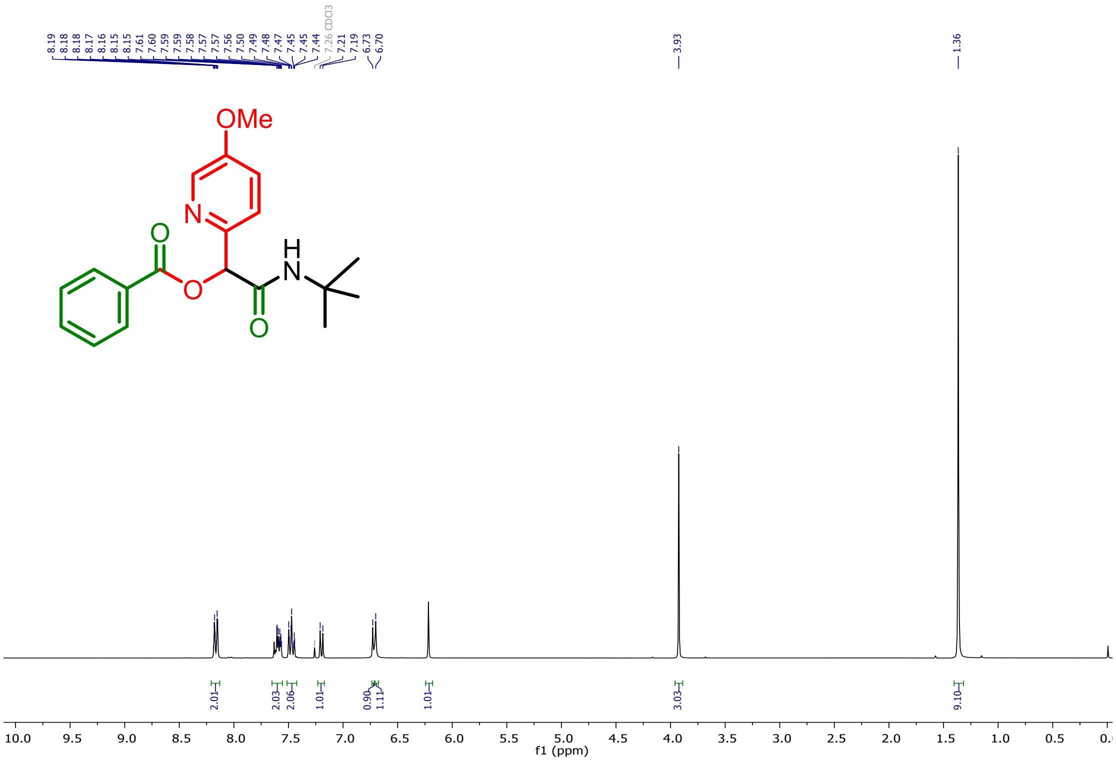


$^{1}H$ NMR spectrum of **6l** (300 MHz, $CDCl_3$).

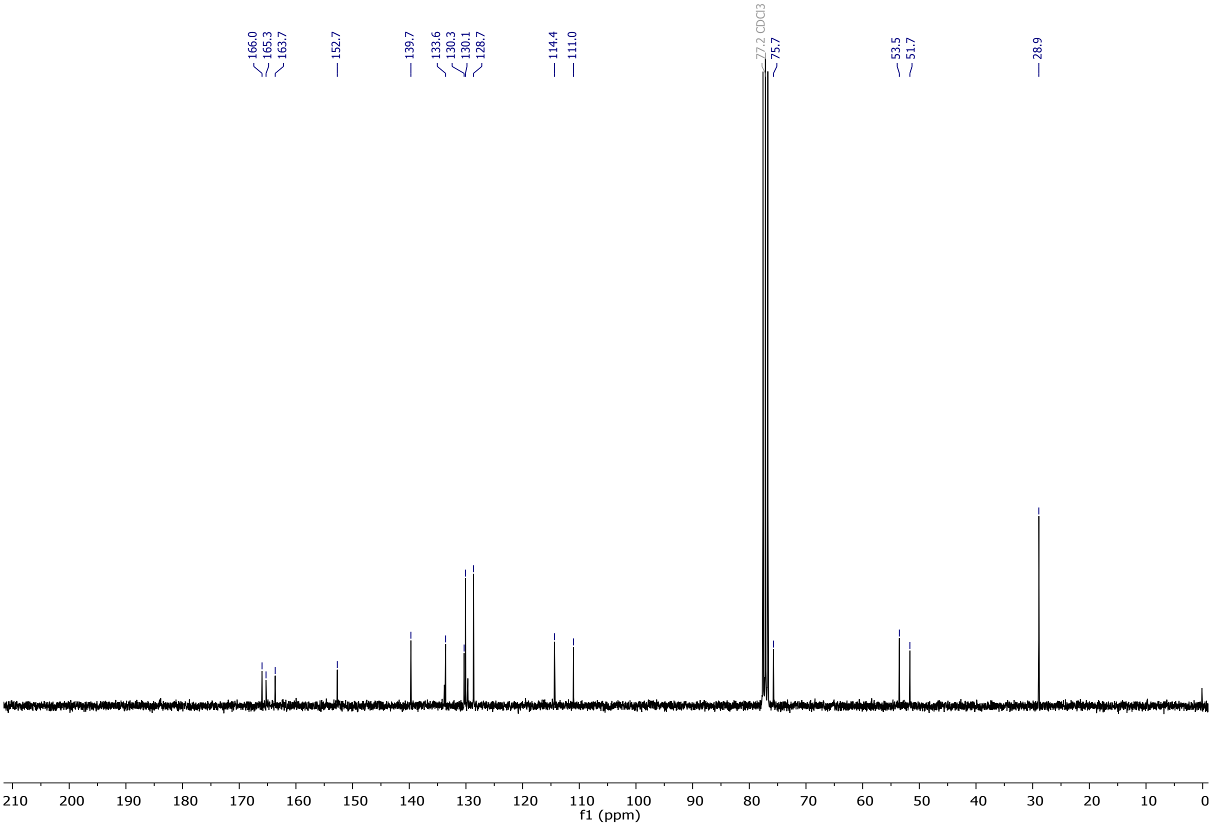


$^{13}C\{^{1}H\}$ NMR spectrum of **6l** (75 MHz, $CDCl_3$).

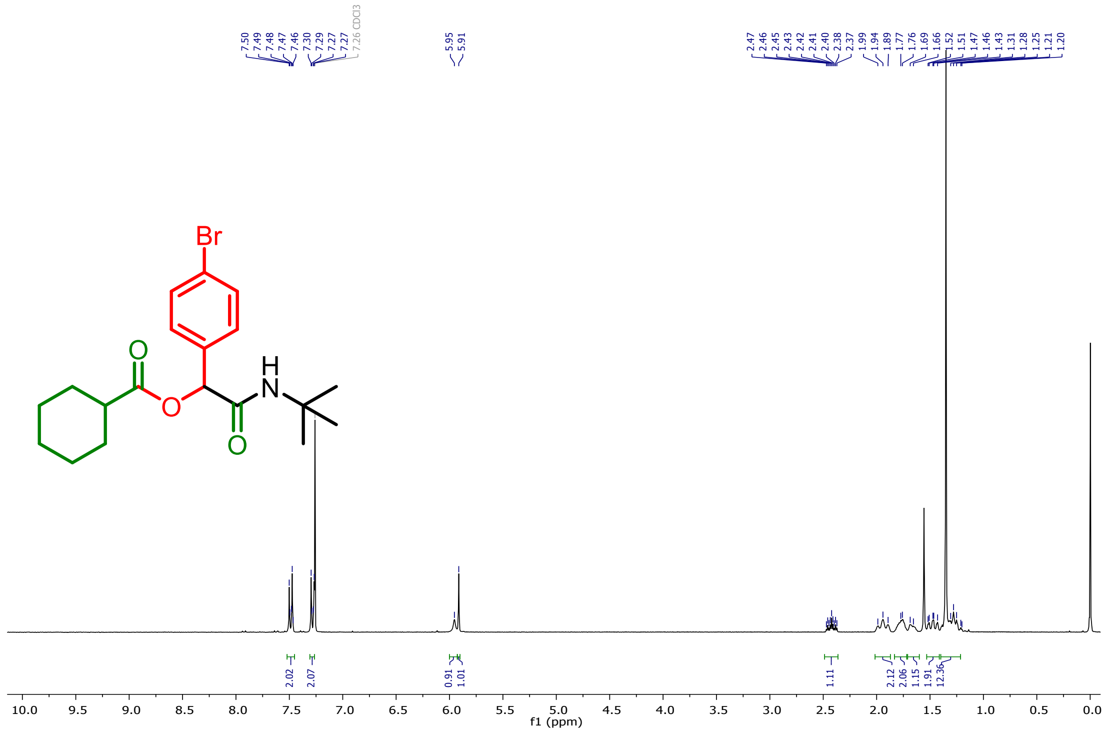


$^{1}H$ NMR spectrum of **6m** (300 MHz, $CDCl_3$).

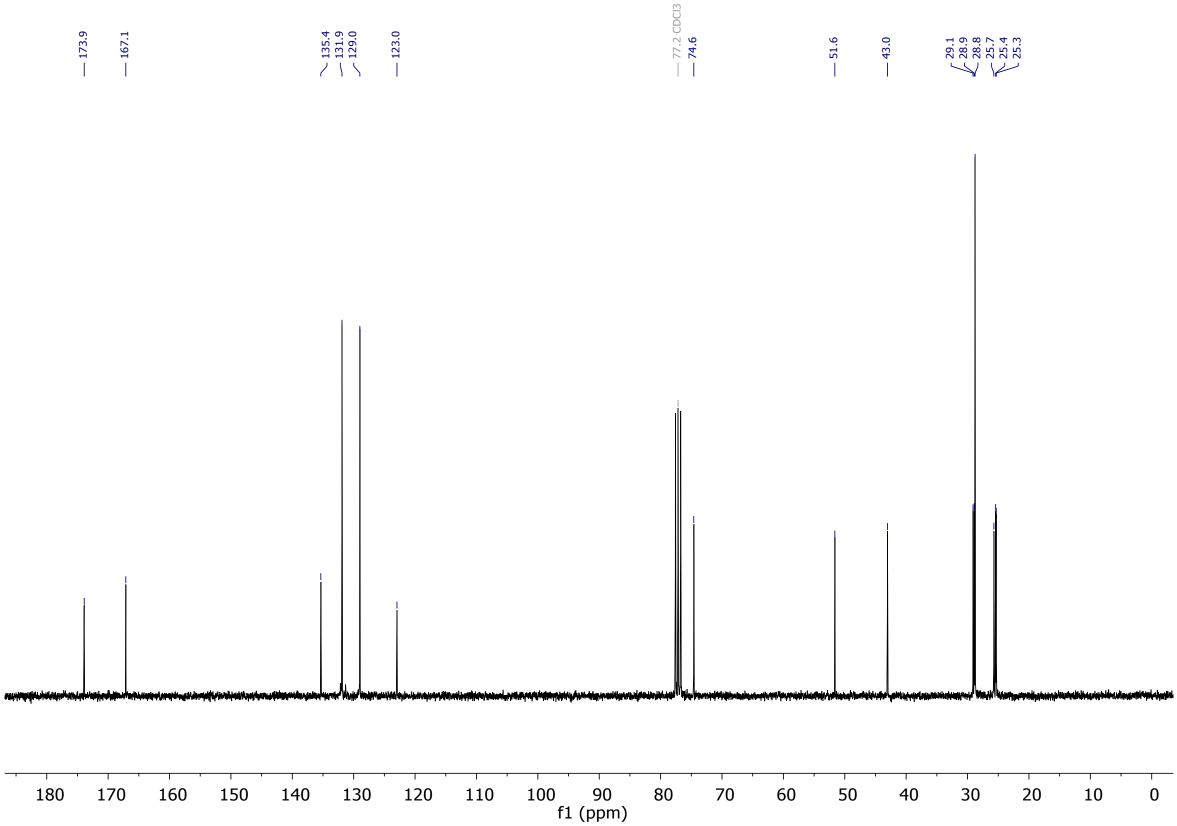


$^{13}C\{^{1}H\}$ NMR spectrum of **6m** (75 MHz, $CDCl_3$).

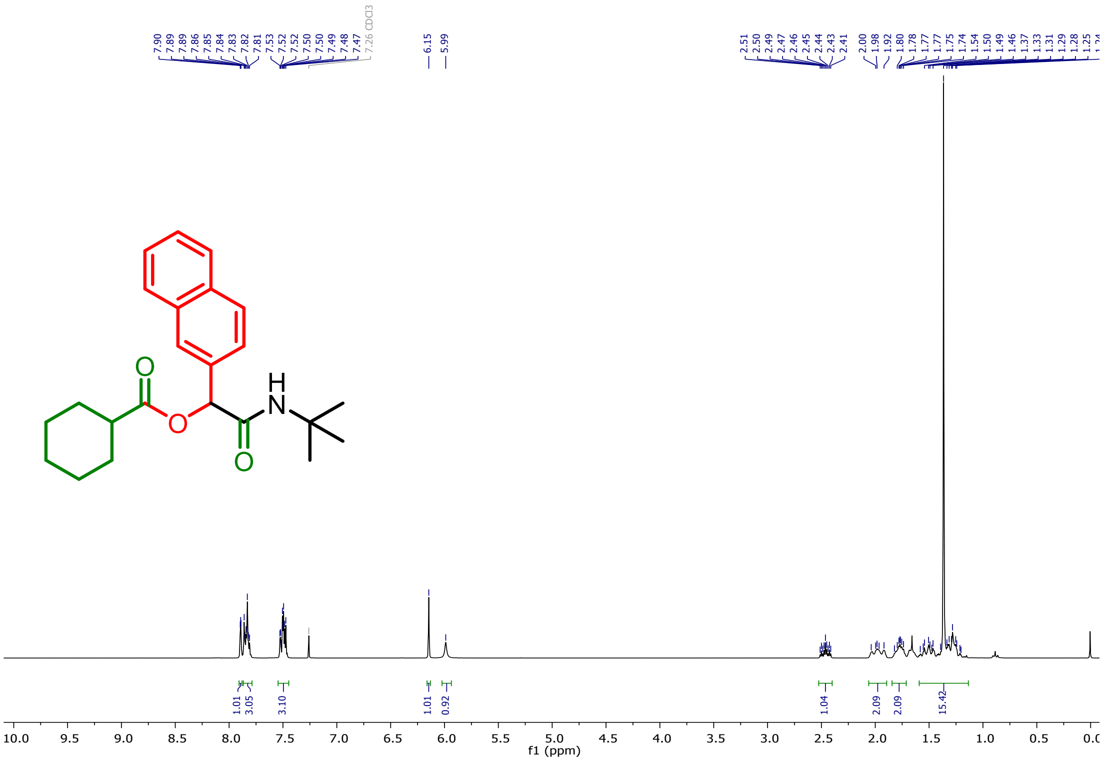


$^{1}H$ NMR spectrum of **6n** (300 MHz, $CDCl_3$).

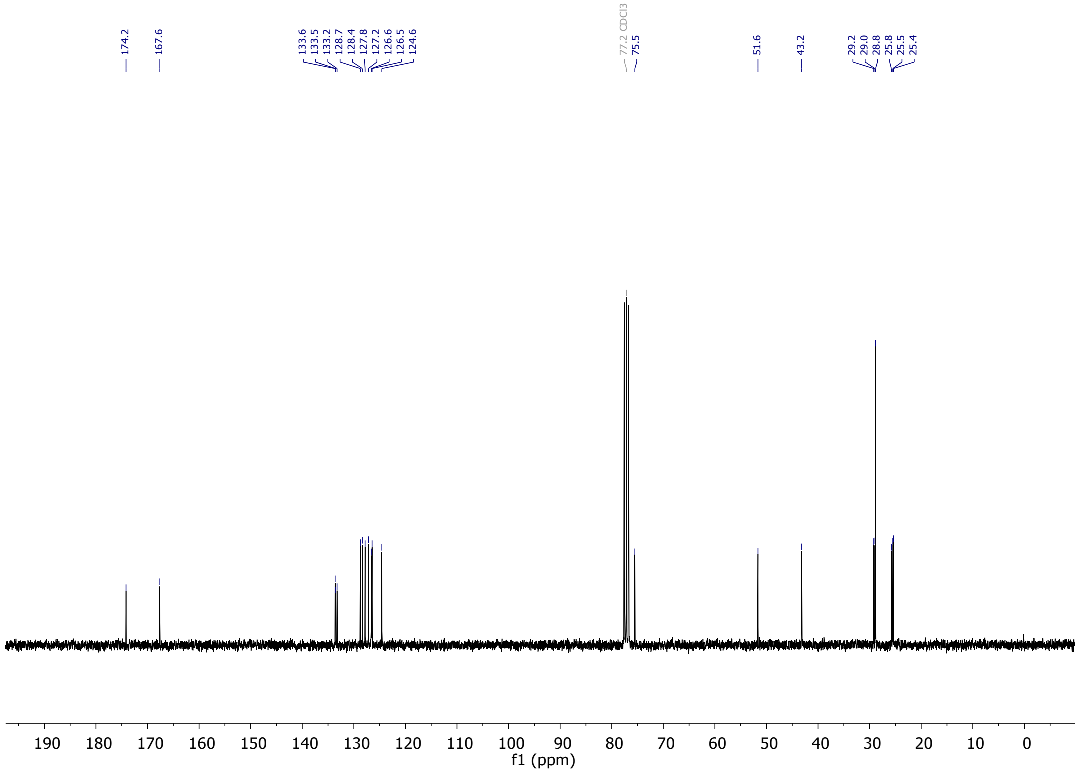


$^{13}C\{^{1}H\}$ NMR spectrum of **6n** (75 MHz, $CDCl_3$).

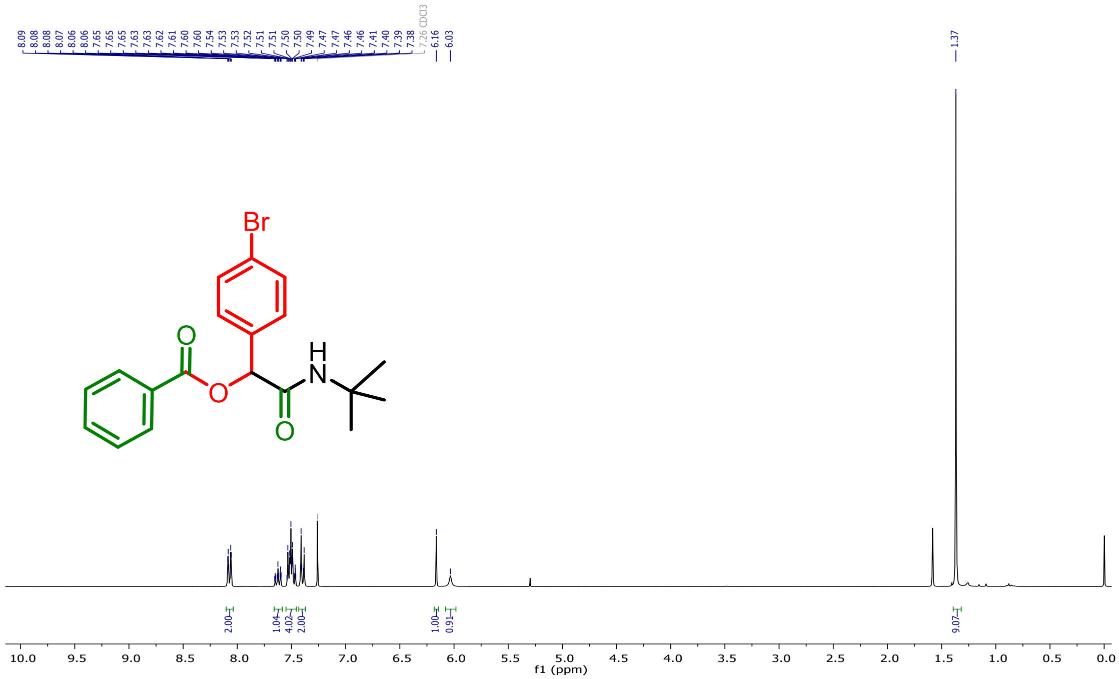


$^{1}$H NMR spectrum of **6o** (300 MHz, $CDCl_3$).

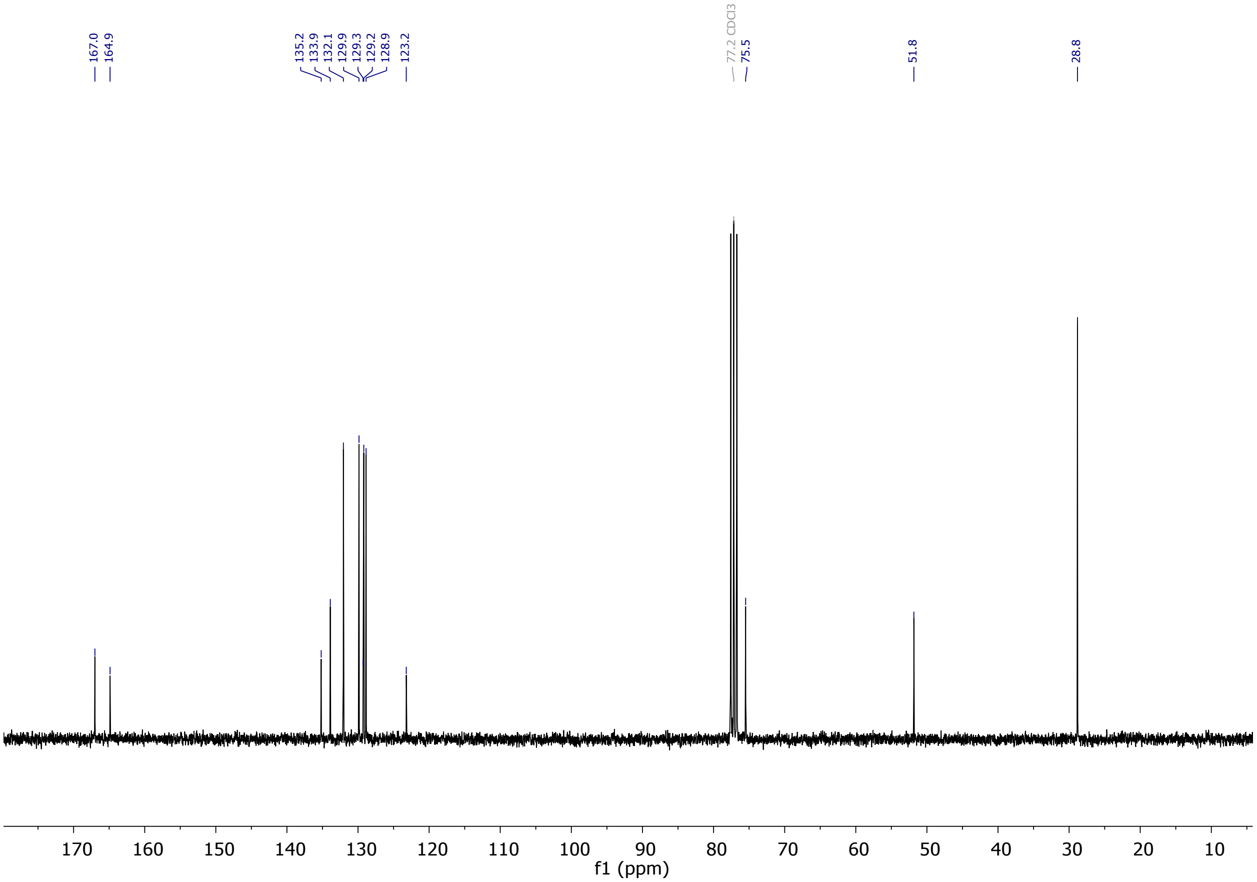


$^{13}$C{$^{1}$H} NMR spectrum of **6o** (75 MHz, $CDCl_3$).

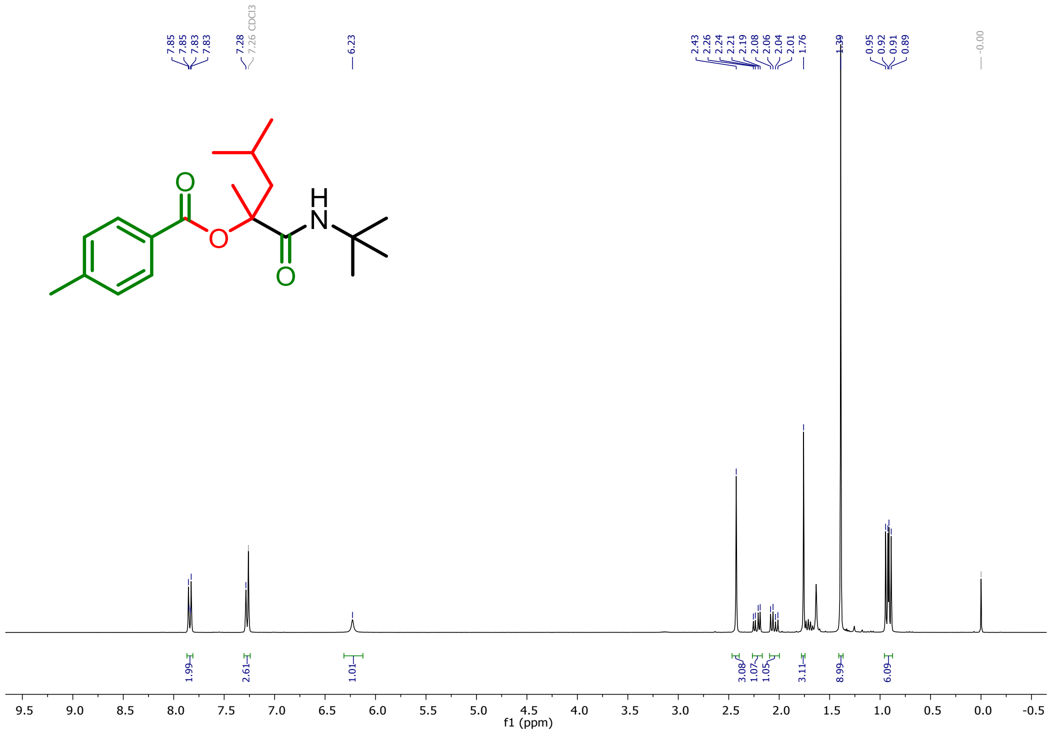


$^{1}H$ NMR spectrum of **6p** (300 MHz, $CDCl_3$).

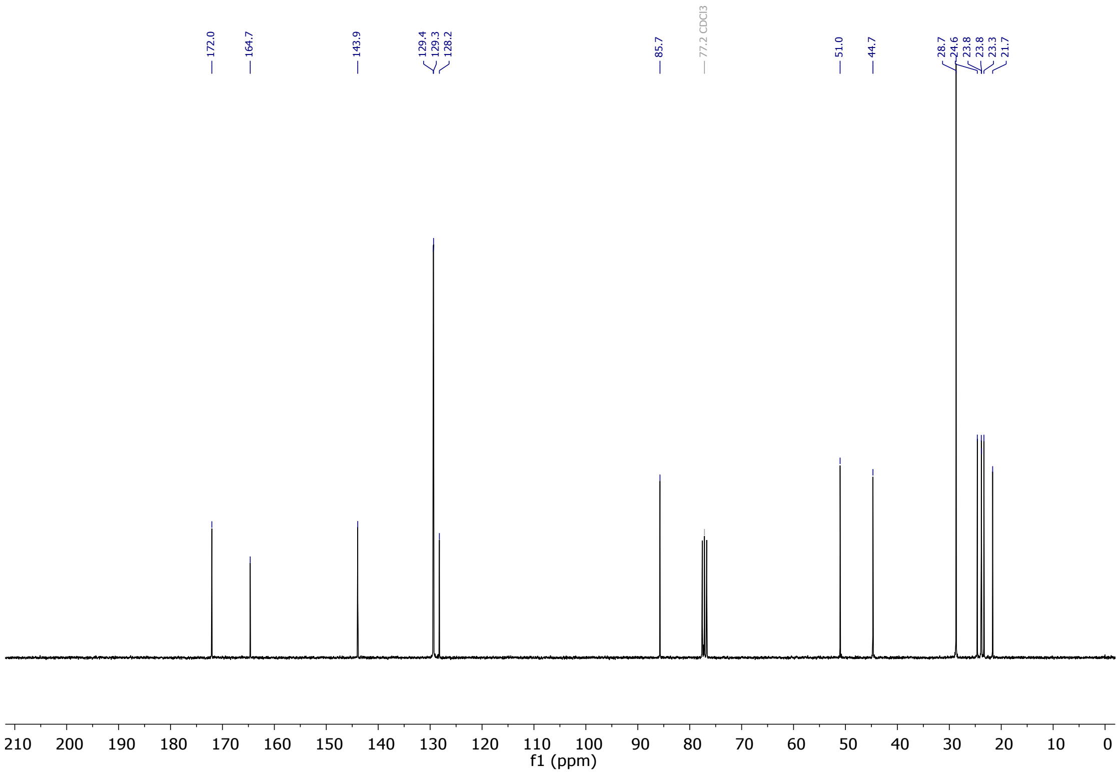


$^{13}C\{^{1}H\}$ NMR spectrum of **6p** (75 MHz, $CDCl_3$).

# 7. References


1 Burés, J. Variable Time Normalization Analysis: General Graphical Elucidation of Reaction Orders from Concentration Profiles. *Angew. Chem., Int. Ed.* **2016**, *55,* 16084-16087.

2 a) G. te Velde, F. M. Bickelhaupt, E. J. Baerends, C. Fonseca Guerra, S. J. A. van Gisbergen, J. G. Snijders, T. Ziegler, Chemistry with ADF. *J. Comput. Chem*. **2001**, *22*, 931–967; b) C. Fonseca Guerra, J. G. Snijders, G. te Velde, E. J. Baerends, Towards an Order-*N* DFT Method. *Theor. Chem. Acc*. **1998**, *99*, 391–403; c) AMS2023.101, SCM Theoretical Chemistry; Vrije Universiteit, Amsterdam, The Netherlands, http://www.scm.com.

3 a) A. D. Becke, *Phys. Rev. A* **1988**, *38*, 3098–3100; b) B. G. Johnson, P. M. W. Gill, J. A. Pople, *J. Chem. Phys.* **1993**, *98*, 5612–5626; c) C. Lee, W. Yang, R. G. Parr, *Phys. Rev. B* **1988**, *37*, 785–789; d) T. V. Russo, R. L. Martin, P. J. Hay, *J. Chem. Phys.* **1994**, *101*, 7729–7737.

4 S. Grimme, S. Ehrlich, L. Goerigk, *J. Comput. Chem.* **2011**, *32*, 1456–1465.

5 E. van Lenthe, E. J. Baerends, J. G. Snijders, *J. Chem. Phys.* **1994**, 101, 9783–9792.

6 E. van Lenthe, E. J. Baerends, *J. Comput. Chem.* **2003**, *24*, 1142–1156.

7 a) M. Franchini, P. H. T. Philipsen, E. van Lenthe, L. Visscher, *J. Chem. Theory Comput.* **2014**, *10*, 1994–2004; b) M. Franchini, P. H. T. Philipsen, L. Visscher, *J. Comput. Chem.* **2013**, *34*, 1819–1827.

8 a) A. Klamt, G. Schüürmann, *J. Chem. Soc. Perkin Trans. 2* **1993**, 799; b) A. Klamt, *J. Phys. Chem.* **1995**, 2224; c) A. Klamt, V. Jonas, *J. Chem. Phys.* **1996**, *105*, 9972; d) C. C. Pye, T. Ziegler, *Theor. Chem. Acc.* **1999**, *101*, 396.

9 a) A. Bérces, R. M. Dickson, L. Fan, H. Jacobsen, D. Swerhone, T. Ziegler, *Comput. Phys. Commun.* **1997**, *100*, 247–262; b) Jacobsen, H.; A. Bérces, D. P. Swerhone, T. Ziegler, *Comput. Phys. Commun.* **1997**, *100*, 263–276. c) S. K. Wolff, *Int. J. Quantum Chem.* **2005**, *104***,** 645–659.

10 C. Y. Legault, CYLview, 1.0b; Université de Sherbrooke, Canada, Sherbrooke, QC, 2009, http://www. cylview.org.

11 RDKit: Open-source cheminformatics. https://www.rdkit.org. **DOI**: 10.5281/zenodo.591637.

12 a) R. L. Martin, P. J. Hay, L. R. Pratt, *J. Phys. Chem. A*, **1998**, *102*, 3565–3573; b) J. Ariai, U. Gellrich, Phys. *Chem. Chem. Phys.*, **2023**, *25*, 14005–14015; c) J. González-Fabra, F. Castro-Gómez, W. M. C. Sameera, G. Nyman, A. W. Kleij, C. Bo, *Catal.Sci.Technol.*, **2019**, *9*, 5433–5440.
